# The Interaction Effects of Age, Sex, APOE and Common Health Risk Factors on Human Brain Functions

**DOI:** 10.1101/2024.08.05.24311482

**Published:** 2024-08-05

**Authors:** Tengfei Li, Jie Chen, Bingxin Zhao, Hui Chen, Changzheng Yuan, Gwenn A. Garden, Kelly S. Giovanello, Guorong Wu, Hongtu Zhu

**Affiliations:** 1 Biomedical Research Imaging Center, School of Medicine, University of North Carolina at Chapel Hill, Chapel Hill, NC, USA; 2 Department of Radiology, School of Medicine, University of North Carolina at Chapel Hill, Chapel Hill, NC, USA; 3 Department of Biostatistics, University of North Carolina at Chapel Hill, Chapel Hill, NC, USA; 4 Department of Statistics and Data Science, the Wharton School, University of Pennsylvania, Philadelphia, PA, USA; 5 School of Public Health, Zhejiang University School of Medicine, Hangzhou, China; 6 Department of Nutrition, Harvard T H Chan School of Public Health, Boston, MA, USA; 7 Department of Neurology, School of Medicine, University of North Carolina at Chapel Hill, Chapel Hill, NC, USA; 8 Department of Psychiatry, School of Medicine, University of North Carolina at Chapel Hill, Chapel Hill, NC, USA; 9 Departments of Statistics and Computer Science, University of North Carolina at Chapel Hill, Chapel Hill, NC, USA; 10 UNC Neuroscience Center, University of North Carolina at Chapel Hill, Chapel Hill, NC, USA; 11 Carolina Insititute for Developmental Disabilities, Chapel Hill, NC, USA; 12 Departments of Genetics, University of North Carolina at Chapel Hill, Chapel Hill, NC, USA

**Keywords:** functional connectivity, lifestyle, cardiovascular risk factors, *APOE*, interaction effects

## Abstract

Recent studies have shed light on the complex nonlinear changes in brain functions across the lifespan, demonstrating the variability in the individual cognitive and neural development during aging. This variability is influenced by factors such as sex, age, genetics, and modifiable health risk factors (MHRFs), which collectively shape unique patterns of brain functional connectivities (FCs) across different regions. However, their joint effects and underlying mechanisms remain unclear. We conduct a comprehensive analysis to jointly examine the association of common risk factors with brain functional measures, using data from 36,630 UK Biobank participants aged 44–81. Participants were assessed for age, sex, Apolipoprotein E (*APOE*) genotypes, ten common MHRFs, and brain FCs measured via resting-state functional magnetic resonance imaging. Using the fine-grained HCP-MMP parcellation and Ji-12 network atlases, we identified 91 associations with network functional connectivity (NFC) and 102 associations with network edge strength (NES) measures. Hypertension, BMI, and education emerged as the top three influential factors across networks. Notably, a negative interaction between sex and *APOE-ε4* (*APOE4*) genotype was observed, with male *APOE4* carriers showing greater reductions in NFC between the cingulo-opercular (CON) and posterior multimodal (PMN) networks. Additionally, a negative age-BMI interaction on NES between the visual and dorsal attention (DAN) networks suggested that higher BMI accelerates the decline in visual-DAN connectivity. A positive age-hypertension interaction between the frontoparietal (FPN) and default mode (DMN) networks indicated a more rapid decrease in functional segregation associated with hypertension. We also identified sex-education interactions, showing more pronounced positive effects on CON-FPN networks in females and PMN-DMN networks in males. Further interactions involving sex and other MHRFs, such as smoking, alcohol consumption, diabetes, and BMI, revealed that smoking, alcohol, and BMI had more detrimental effects in males, while diabetes had a more pronounced negative impact in females within specific networks. These findings underscore the necessity of jointly considering sex, age, genetic factors, and MHRFs to accurately delineate the multifactorial alterations in the FCs during brain aging.

## Introduction

Recent investigations into the normative lifespan trajectories of brain morphology and the functional connectome ^1,2^ have highlighted large variability of brain structural and functional aging. This variability is shaped by a diverse array of factors, including sex, age, genetics, and modifiable health risk factors (MHRFs), such as socioeconomic status (SES), lifestyle and cardiovascular risk factors (CVRFs). In our previous work ^3^, we conducted a comprehensive analysis of how these health risk factors and their interactions influence brain structure during aging. Building on this foundation, it is essential to examine how these elements collectively affect brain functionality.

Age, sex, and Apolipoprotein E (*APOE*) genotypes are three major non-modifiable risk factors extensively studied for their impact on brain functionality during aging ^4^. Cognitive changes are a well-documented aspect of the normal aging process, varying across different cognitive subdomains ^5^. For instance, vocabulary often remains resilient to brain aging ^6^ while conceptual reasoning, memory, and processing speed tend to decline gradually over time ^7^. These patterns can be attributed to aging-related alterations in brain functional networks, including a general decrease in both within- and between-network connectivity ^8–11^, the posterior-anterior shift in aging (PASA) ^10,12,13^ characterized by compensatory increases in task-positive network connectivity, reduced default mode network (DMN) connectivity ^14^, and a decrease in the segregation of network activities ^15,16^. *APOE-ε4* (*APOE4*), a major genetic risk factor for Alzheimer’s disease, has been associated with reduced DMN connectivity ^17^, altered connectivity in memory and cognitive networks ^18,19^, accelerated age-related reduction of local interconnectivity ^20^ and occasionally increased hyperconnectivity ^21^. Associations of *APOE4* have displayed sex-dependent ^22^ and age-dependent ^23^ variations. Some studies suggest that *APOE4* has more pronounced effects on attention functions in women and on memory and executive functions in men ^24^. However, most existing literature focuses on the vulnerability of female *APOE4* carriers ^3,22,25^, with limited research on brain functional atrophy in males. Furthermore, the combined impact of age, sex, *APOE*, and other MHRFs on brain functions across various networks remains unclear.

A range of MHRFs, particularly CVRFs, lifestyle, and socioeconomic status (SES) factors, have been extensively studied concerning brain and cardiovascular health. Key CVRFs, initially identified in the Framingham Heart Study ^26^, include hypertension, smoking, cholesterol levels, diabetes, obesity, left ventricular hypertrophy, family history of premature coronary heart disease, and estrogen replacement therapy ^27–30^. The American Heart Association’s Life’s Essential 8 outlines 8 critical lifestyle measures for cardiovascular health, including three additional factors, the diet, physical activity and sleep ^31^. Recent studies ^32^ demonstrate a strong link between the structural and genetic attributes of the heart and brain. Disruptions in cardiovascular function due to risk factors can impair brain health, influencing both cardiovascular and brain aging ^33^ or neurodegenerative diseases ^34^. The 2020 Lancet Commission report ^35^ suggests that modifying 12 major dementia risk factors (DRFs), including hypertension, diabetes, obesity, smoking, alcohol use, physical inactivity, depression, hearing loss, brain injury, air pollution, education and social isolation could potentially prevent or delay around 40% of dementia cases.

**Figure 1.**
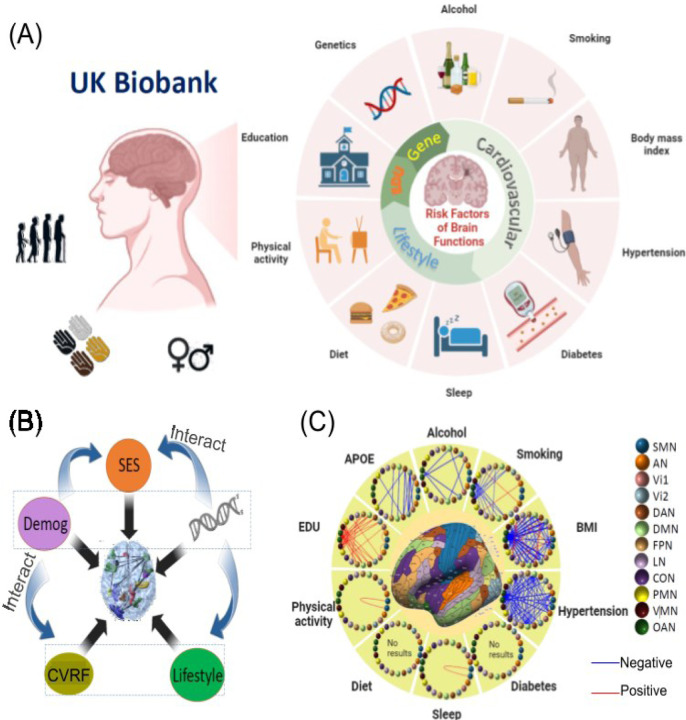
The study design. **(A)** Multi-omics data in the UKB. **(B)** A schematic diagram illustrating the associations we analyzed between genetics, demographics, cardiovascular, lifestyle, and SES factors, and brain structural traits in this study. **(C)** Identified brain network pairs based on the Ji-12 atlas with network edge strength (NES) measures associated with APOE, alcohol, smoking, BMI, physical activity, sleep, education, and hypertension (main effects only; no interactions considered). SMN: somatomotor network; AN: auditory network; Vi1, Vi2: visual networks; DAN: dorsal attention network; DMN: default mode network; FPN: frontoparietal network; LN: language network; CON: cingulo-opercular network; PMN/VMN: posterior/ventral multimodal network; OAN: orbito-affective network; SES: socioeconomic status (such as education); Demog: demographics; EDU: education; APOE: apolipoprotein E gene; CVRF: cardiovascular risk factors.

The aim of this study is to perform a comprehensive association analysis between brain FCs and various risk factors, including *APOE* genotype, age, sex, ten MHRFs, as well as their interactions. The ten primary MHRFs include six adverse factors—hypertension ^36–40^, diabetes ^41–43^, smoking ^44–46^, obesity ^47–49^, excessive alcohol consumption ^50,51^, and social deprivation ^52^, that have been extensively studied for their association with disrupted brain functional network connectivity, leading to cognitive impairments. Additionally, four beneficial MHRFs—higher education ^53–55^, physical activity ^56,57^, healthy diet ^58,59^ and adequate sleep ^60^ are included as they have been shown to enhance functional connectivity, promote more segregated and resilient functional networks ^61,62^ and improve the neuroplasticity ^63^. We utilized resting-state functional magnetic resonance imaging (rfMRI) data to assess brain functional network connectivity and coherence in 36,630 UKB subjects. Figure 1 illustrates the study design to integrate the diverse data sources. The large sample size provides robust statistical power, enhancing the replicability of our findings. This study is expected to offer new insights into the dynamics of normal aging and preclinical dementia risks, particularly concerning health disparities, by examining complex FC networks within the brain.

## Methods

### Study Population

The UKB study has provided an extensive dataset encompassing half a million participants, with over 40,000 of these individuals having undergone MRI scans. Upon processing this data, we were able to extract a subset of 39,354 subjects with both the T1-weighted and resting-state functional MR scans. These subjects, including 36,339 and 3015 subjects with British and non-British ancestry, respectively, drawn from phase 1 (released in 2016), phase 2 (newly released in 2018), and 3 (newly released in 2020) of the UKB study, contributed data that included genetic factors, lifestyle choices, and CVRFs. Our brain functional analyses were conducted on a cohort of 36,630 unrelated individuals, each of whom had complete data on brain functional imaging metrics along with genetic and lifestyle information. The primary findings of our study were derived from 33,824 white British subjects, and were subsequently extended to the remaining 2806 subjects of non-British ancestry for validations.

### Imaging Data Processing

The image acquisition and preprocessing procedures were detailed in the UKB Brain Imaging Documentation (https://biobank.ctsu.ox.ac.uk/crystal/crystal/docs/brain_mri.pdf). All UKB brain imaging datasets, including the resting-state fMRI (rsfMRI) and the T1-weighted structural MRI data were acquired from standard Siemens Skyra 3T scanners. The rsfMRI data were acquired using a blood oxygen level-dependent (BOLD) sequence and an echo-planar imaging (EPI) sequence (TR = 0.735 s, TE = 39 ms, FoV = 88 × 88 × 64, voxel resolution 2.4 mm × 2.4 mm × 2.4 mm, a multiband factor of 8, no iPAT, flip angle 52◦, and fat saturation), lasted approximately 6 minutes. In addition, the T1-weighted sMRI scans were acquired at the isotropic resolution of 1 mm and a dimension of 208×256×256 matrix, with a TR of 2000 ms, an inversion time of 880 ms, a TE of 2 ms, an in-plane acceleration of 2, and a scan time of 5 min using straight sagittal orientation.

Our imaging analyses followed the rsfMRI preprocessing workflow provided by the UKB ^64^ ( https://git.fmrib.ox.ac.uk/falmagro/UK_biobank_pipeline_v_1), which includes motion correction, grand-mean intensity normalization, high-pass temporal filtering, EPI unwarping, gradient distortion correction (GDC), Independent Component Analysis (ICA)-based X-noiseifier (FIX) artifact removal and image registration. Structural artifacts were removed using FMRIB’s ICA-FIX processing ^65–67^. The GDC-corrected rsfMRI data were co-registered with the high-resolution T1 MRI image, which were registered to the MNI-152-2mm standard space. Subjects with unusable, low-quality T1 MRI scans, as identified by the UKB brain imaging team ^64^ were excluded in our analyses. Meanwhile, the head motion parameters (UKB data field 25741) were calculated by averaging the framewise displacement (FD) across the brain for each consecutive pair of time points for each rsfMRI scan, which were then averaged across all time points, which were included as confounding covariates in our association analyses.

The regions of interest (ROIs) used to construct the network imaging-derived phenotypes (IDPs) were selected as Glasser 360 (HCP-MMP) atlas ^68^, corresponding to 360 cortical regions classified into twelve resting-state networks following the Ji-12 network atlas ^69^, namely, the somatomotor (SMN), auditory (AN), visual1 (Vi1), visual2 (Vi2), dorsal attention (DAN), default mode (DMN), frontoparietal (FPN), language (LAN), cingulo-opercular (CON), posterior multimodal (PMN), ventral multimodal (VMN), and orbito-Affective (OAN) networks. For each rsfMRI scan, the mean time series from each of 360 ROIs were extracted and the correlation between each pair of regional time series was transformed from Pearson correlations (r-values) to z-statistics using Fisher transformation. This yielded a 360 × 360 FC matrix for each subject in the datasets, representing 64,620 connectivity traits, using the FSLNets (http://fsl.fmrib.ox.ac.uk/fsl/fslwiki/FSLNets) toolbox. Next, for each pair of the 12 networks we calculated the between- and within-network functional connectivity (NFC) and edge strength (NES) measures as follows,

$$Between-network\;NFC\left(I1,I2\right)=\sum_{i1\in I1,i2\in I2}FC\left(i1,i2\right)/\left[\#I1\#I2\right],$$

$$Between-network\;NES\left(I1,I2\right)=\sum_{i1\in I1,i2\in I2}\left|FC\left(i1,i2\right)\right|/\left[\#I1\#I2\right],$$

$$Within-network\;NFC\left(I\right)=\sum_{i1,i2\in I,i1\neq i2}FC\left(i1,i2\right)*2/\left[\#I\left(\#I-1\right)\right],$$

$$Within-network\;NES\left(I\right)=\sum_{i1,i2\in I,i1\neq i2}\left|FC\left(i1,i2\right)\right|*2/\left[\#I\left(\#I-1\right)\right],$$

where I, I1 and I2 are the networks of interest, $FC\left(i1,i2\right)$ is the regional FC calculated between two ROIs i1 and i2, and #I denotes the number of ROIs in the network I. We also generated 100 × 100 FC matrices based on Schaefer 100 parcellation atlas and network-level traits for the Yeo-7 and Yeo-17 atlases ^70^. For each of the FC, NFC and NES traits, subjects with extreme deviations—defined as values exceeding five times the median absolute deviation from the population median—were flagged as outliers. These outlier data points were excluded from the subsequent association analyses.

### APOE Genotyping

The *APOE* gene, particularly the ε4 allele, is acknowledged as a significant risk factor for Alzheimer’s disease (AD). Determination of the *APOE* genotype relies on two single nucleotide polymorphisms (SNPs), rs429358 and rs7412, which define three alleles—*APOE-ε2*, *APOE*-*ε3*, and *APOE*-*ε4*—and result in six possible genotypes. These genotypes are: *APOE*-*ε2*/*ε2*, *APOE*-*ε2/ε3*, *APOE*-*ε2/ε4*, *APOE*-*ε3/ε3*, *APOE*-*ε3/ε4*, and *APOE*-*ε4/ε4*. *APOE*-*ε2* is associated with a decreased risk of AD and is the rarest allele, whereas *APOE*-ε3, the most common allele, is considered neutral in terms of risk. *APOE*-*ε4*, conversely, is associated with an increased risk of AD ^71^.

Utilizing imputed SNP data from UKB resources, *APOE* genotyping was conducted on the 36,630 individuals. The genotype distribution is as follows: *APOE*-*ε4/ε4* is present in 806 subjects (2.2%), *APOE*-*ε2/ε2* in 211 subjects (0.6%), and *APOE*-*ε3/ε3* in 21,688 subjects (59.2%). Additionally, 4,545 subjects (12.4%) possess the *APOE*-*ε2/ε3* genotype, 877 subjects (2.4%) have *APOE*-*ε2/ε4*, and 8,503 subjects (23.2%) are *APOE*-*ε3/ε4*. Gender-wise distribution among these genotypes is detailed, with allele frequencies for *ε2*, *ε3*, and *ε4* being 8.0%, 77.1%, and 14.9%, respectively. Demographics for each genotype group of British and Non British participants are outlined in Supplementary Table S4 and S5. Our analysis incorporated the count of the APOE-ε4 and APOE-ε2 alleles as individual covariates in statistical models. This approach allows for the assessment of the additive effects attributed to each allele. It differs from that of some prior studies which grouped individuals with the *APOE-ε4* allele into homozygotes and all others, or classified individuals as *APOE-ε2* carriers if they were homozygous for *APOE-ε2* or had *APOE-ε2/ε3* genotype, considering the remaining as controls.

### Lifestyle and SES Factors

In our study, the selection of lifestyle variables followed the criteria established in the research on healthy lifestyle and dementia by Lourida et al. (2019) ^72^. These variables, collected via touchscreen questionnaires during the UKB baseline assessment, include smoking status, physical activity, diet, and alcohol consumption—factors known to be associated with brain health. Specifically, we opted to use ever-smoking status (encompassing both ever-smokers and never-smokers) instead of current smoking status. This approach allows us to include a larger proportion of cases, thereby enhancing our statistical power. Regular physical activity, as per the American Heart Association (AHA) guidelines, is defined as engaging in at least 150 minutes of moderate exercise or 75 minutes of vigorous activity weekly. The diet was considered healthy if participants met at least four of the seven cardiometabolic health dietary recommendations outlined by Mozaffarian (2016) ^73^, which include the intake of vegetables, fruits, fish, unprocessed meat, processed meat, whole grains, and refined grains. Alcohol consumption was stratified into abstinence, moderate consumption, and excessive consumption. This categorization is due to the mixed evidence regarding the effects of moderate drinking, as highlighted by studies from Daviet et al. (2022) ^74^ and Sabia et al. (2018) ^75^. Moderate alcohol consumption is capped at 14 grams per day for women and 28 grams per day for men, while abstinence encompasses individuals who do not drink or only consume alcohol on special occasions, amounting to zero units per day. Additionally, we have considered sleep duration as a lifestyle variable, given its association with dementia risk as described by Palpatzis et al. (2022) ^76^, categorizing short and long sleep duration as less than 6 hours or more than 8 hours, respectively. Beyond these lifestyle factors, our analysis also incorporated SES elements such as education level and social deprivation. Education level was divided into individuals with or without a college or university degree or higher. Social deprivation was measured using the Townsend deprivation (SoDep) index based on each participant’s postcode, which we dichotomized at the median of the index into two groups: those above and those below the median. The methodology for generating these lifestyle and SES factors, along with a more detailed discussion, can be found in the Supplementary Table S1.

### CVRFs

Cardiovascular diseases (CVDs) are the leading cause of mortality worldwide, highlighting the critical need for a deeper understanding of risk factors to alleviate the growing health burden. Several lifestyle factors, including ever-smoking and alcohol consumption, are also recognized as CVRFs. In addition to the above lifestyle factors, diabetes, body mass index (BMI), and hypertension are three large CVRFs that contribute to the development and progression of CVD, which are the primary focus of our analyses. Diabetes mellitus, characterized by chronic hyperglycemia, not only damages the vascular endothelium but also intensifies the complexity of atherosclerotic plaque, thereby significantly increasing cardiovascular risk; obesity, measured by the a high body mass index (BMI), is another critical risk factor that frequently coexists with metabolic abnormalities, leading to atherosclerotic cardiovascular disease. Furthermore, hypertension is a silent yet formidable risk factor that plays a substantial role in most cardiovascular events. It imposes an excessive burden on arterial walls, leading to structural and functional impairments of the vasculature. In our analysis, BMI was treated as a continuous variable (UKB data field 21001), whereas diabetes and hypertension were categorized as binary disease status variables, based on their presence in medical records according to the International Classification of Diseases, 10th Revision (ICD-10; UKB data-Field 41270).

### Confounding Covariates

In our analysis of the effects of the risk factors, including the APOE gene, lifestyle factors, SES, and CVRFs, we controlled for the following confounding variables: study phase of participants, age at the time of imaging, age squared, sex, interactions between sex and age, interactions between sex and age squared, partnership status (whether living with a partner; UKB data field 6141), head motion, brain volume scaling (UKB data field 25000), and brain positioning within the scanning field (UKB data fields 25756, 25757, and 25758).

### Statistical Analysis

We carried out four levels of multivariate linear regression models on the white British UKB subjects to answer the following questions: (1) Is there the *APOE* genotype and age interaction effect on brain FCs? (2) Are there environmental (healthy lifestyle factors, SES, and CVRFs) and age interaction effects on brain FCs? (3) Does the age interaction effect vary between males and females? (4) Are there *APOE* genotype and environmental factor interaction effects on brain FCs and are they sex-dependent?

To answer questions (1), we used the between- and within-network NFC and NES measures across three network atlases, the Ji-12, Yeo-7 and Yeo-17 as the dependent variables, while taking the *APOE*4 and *APOE*-ε2 counts, sex, age, CVRFs, lifestyle factors, UKB study phase, partnership status, sex and age as predictors. To investigate the effect of *APOE* genotype, environmental factors, and age interaction on FC measures in question (1) and (2), we added the two-way interaction terms of age with *APOE4*, lifestyle, SES and CVRFs, separately, to our models. For identifying the difference of above interaction effect on FC measures in different sex groups in question (2), the three-way interaction term of age, sex and *APOE* and environmental factors, and three two-way interaction terms between them were included in the regression models. To address question (4), we included *APOE*-environmental interactions and sex-*APOE*-environmental interactions, separately. When considering each risk factor and its interactions, we controlled for the main effects of all other risk factors to assess its conditional effects. Detailed statistical models we considered for the association between main covariates, two-way and three-way interactions and brain function were displayed in the Supplementary Table S2. Type II ANOVA F-test ^77^ were used to determine the significance of main effects and two-way interaction effects.

Our main findings were derived from white British UKB subjects. The associations in our main findings were considered significant if they met the Bonferroni-corrected significance level of *0.05/m*, where the number of tests *m* was detailed in the Supplementary Table S3. We validated our main findings using results in the non-British UKB populations, by demonstrating consistent association directions across the two populations. Additionally, after network-level analyses, we conducted post hoc analyses on specific ROIs within the identified networks for each risk factor to investigate associated connectivity between ROI pairs. The same confounding factors controlled in the network-level analyses were accounted for in the ROI-level analyses, and a Bonferroni-corrected significance level was applied to control for the number of ROI pairs within each specific network pair.

## Results

The demographic information for the 36,630 UK Biobank subjects, are summarized in Table 1. Correlation plots for genetic factors and environmental (lifestyle, CVRFs and SES) factors (Supplementary Figure S1) indicate weak correlations (*r < 0.3*) except for between moderate and excessive alcohol consumption. Supplementary Figures S43–S45 depict the population-mean NFC and NES matrices for 36,630 subjects using Ji-12, Yeo-7, and Yeo-17 atlases, respectively.

These figures reveal that anti-correlations exist between the VMN and all the other networks except DMN and OAN networks (Ji-12), between the DMN and the AN, CON, DAN, PMN, SMN, Vi1, and Vi2 networks (Ji-12), and between the limbic (LN) and FPN, DAN, and Vi networks (Yeo-7). These anti-correlations, which may signify competitive or complementary functions within the brain, are essential for efficient cognitive processing. They may facilitate the segregation of various cognitive functions across distinct neural networks.

Our primary findings were derived from multiple linear regression analyses of various IDPs in white British subjects, and validated in non-British cohorts. Demographic characteristics, including 13 variables, are detailed in Supplementary Tables S4 and S5 for the two populations. The differences of each variable between the populations were assessed using the two-sided two-sample t-test for continuous variables (BMI and age) and two-proportions Z-test for other categorical variables. Relative to the white British cohort, the non-British population is, on average, two years younger and has a lower proportion of ever-smokers (−3.3%) and individuals living with partners (−6.9%). Additionally, the non-British group exhibits a higher percentage of individuals with an advanced education (14.1%), a larger SoDep index (17.1%), excessive alcohol consumption (10.8%), and diabetes (1.5%). No other covariate differences passed the Bonferroni-corrected significance level of *p < 0.0038*.

In summary, our study delineates the effects of CVRFs, lifestyle, and SES factors on brain functions, along with their interactions with sex, age, and the *APOE* gene. The numbers of findings for each category—*APOE4*, cardiovascular, lifestyle, socioeconomic, and their interactions—are summarized across the three atlases (Ji-12, Yeo-7, Yeo-17) and the NFC and NES measures in Supplementary Table S6, and depicted in Supplementary Figures S37–S42. Specifically, the main effects of demographic, *APOE4*, lifestyle factors, SES, and CVRFs are presented in Supplementary Figures S2–S7. The two-way and three-way interaction effects are illustrated in Supplementary Figures S8–S13 and S14–S19, respectively. In the white British population, we identified 113, 54, and 123 associations between NES measures and health factors, along with their interactions with age, sex, and the APOE gene (excluding age, sex, and age*sex effects), for the Ji-12, Yeo-7, and Yeo-17 atlases, respectively (Supplementary Tables S3 and S6). Of these, 102 (90.3%), 49 (90.7%), and 110 (89.4%) are validated in non-British populations in the same directions; for NFCs measures, 113, 68, and 185 associations are identified, with 91 (80.5%), 55 (80.9%), and 154 (83.2%) validated, respectively, in the same directions. Additionally, the effect sizes of identified associations for NFCs and NESs in the main discovery cohort are largely consistent with those in the non-British dataset (Supplementary Figures S31–S36), indicating that the associations are robust across ethnicities.

### Age, Sex and APOE Associations with Brain Functional Architecture

**Figure 2.**
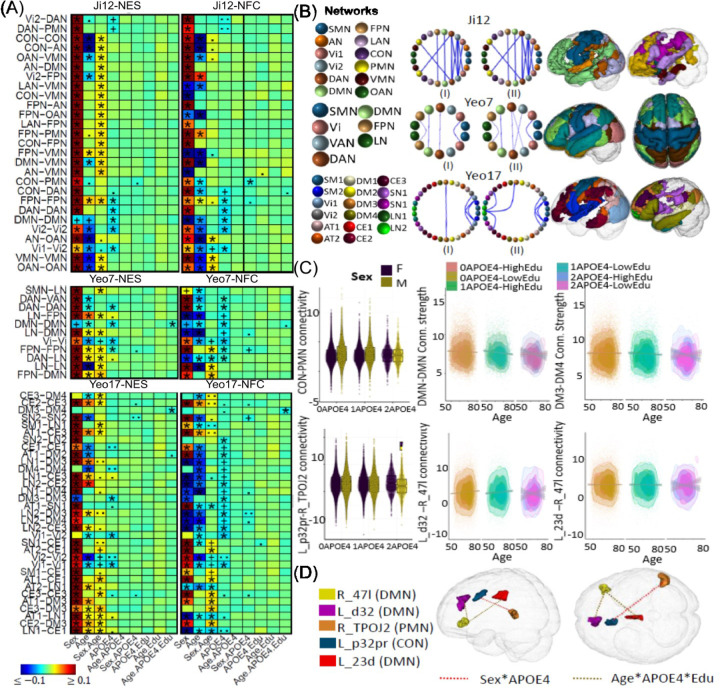
Selected association results on *APOE4* gene and its interactions with age, sex and environmental factors on brain network functional connectivity (NFC) and edge strength (NES) measures. (A) Heatmaps display association results from the white British population using three network atlases: Ji-12, Yeo-7, and Yeo-17, for NES (Panel I) and NFC (Panel II). Results that passed the Bonferroni-corrected significance threshold and were confirmed by the non-British population are marked with (*); significant results not validated by the non-British are indicated by (+). (.) and (..) denote non-significance with p-values less than *1e-4* and *1e-3*, respectively. (B) Circular plots showing association results based on three network atlases Ji-12, Yeo-7 and Yeo-17 in three rows for the NES in (I) and NFC in (II), respectively. Network spatial locations are also displayed on the right panel, where different colors were used to visualize different networks. (C) Boxplots and scatterplots to illustrate *APOE-ε4* interaction effects on both the network-level and regional-level connectivities. Row 1: sex-*APOE* effect on the CON-PMN between-network NFC based on Ji-12 atlas, and age-*APOE*-education interaction effects on the within-DMN NES from Yeo-7 atlas and on DM3-DM4 between-network NES from Yeo-17 atlas are shown in column 1–3, respectively, Row 2: sex-*APOE* effect on the brain functional connectivity (FC) between brain regions L_P32pr and R_TPOJ2, and age-*APOE*-education interaction effects on the brain FC between Ld32 and R47I, and between L_23d and R_47I were shown in column 1–3, respectively, (D) Spatial locations of the identified brain regions for the sex-*APOE4* and age-*APOE4*-education interactions.

The associations between age, sex, the *APOE* gene, and their interactions with network connectivity measures are depicted in Figure 2A. Consistent with previous research ^78–80^, a general decreasing trend in both of the within- and between-network NES measures is observed with aging, except for increasing trends observed within the FPN and between the FPN and the DAN and VMN networks (Supplementary Figure S2). Aging has been associated with decreased intra- and inter-network connectivity during rest and task ^8^ except the FPN. The exception of FPN-DAN connectivity aligns with previous study ^12^, which demonstrates increased between-network connections in older adults compared to younger adults within the FPN and DAN networks. Research findings regarding within-FPN connectivity are mixed ^81^. Some of them indicate weak negative changes with aging ^82,83^, whereas others report positive ^84^ or insignificant ^85^ associations. The altered FC associations between the FPN and other networks may indicate adaptive reorganization of brain networks to sustain cognitive function during neural decline ^9,86,87^. In addition, results from Yeo-7 atlas exhibited increased between-network connectivity strength with aging, including DMN-DAN, DAN-LN, and LN-DMN.

Regarding sex differences, males exhibited higher NES measures across all networks, except for the DMN, where females demonstrated higher connectivity. This exception aligns with previous literature, which reports higher DMN connectivity in women ^88,89^. Additionally, positive age-sex interaction effects were identified in 18 within- and between-network NESs (Ji-12), indicating a more pronounced increase within and between FPN connections and less reduction between the AN, DMN, VMN, and CON in males with aging. Observations from the Yeo-7 atlas also revealed more pronounced increases in NESs between DMN-LN and DAN-LN networks, as well as more reduction within the Vi network and less reduction within the LN network in males. Overall, hyperconnectivities related to the FPN and LN networks and reductions in the visual network with aging were more evident in males, while reductions in connectivity strength between AN, DMN, VMN, and CON networks with aging were more pronounced in females. This differentiation across networks may explain the severe brain structural atrophy observed in males during normal aging ^3^, whereas connectivity reductions in females may involve more complex neurobiological changes related to neurodegenerative diseases ^90^.

Regarding the effects of the *APOE* gene, no significant effects of the *APOE2* variant were identified. However, findings on *APOE4* carriers with reduced connectivity strength are in line with previous studies ^17^. Specifically, negative effects of the *APOE4* variant were observed on the NES within the Vi2, DAN, DMN, and FPN networks, as well as between the Vi1-Vi2, Vi2-DAN, and DAN-PMN networks. For interactions, we identified the negative sex-*APOE4* effect (Figure 2C) on the NFC between the CON and PMN networks (ES: *−0.020*, *p = 5.2E-4*), indicating reduced CON-PMN connectivity in male *APOE4* carriers. Further ROI-level analyses revealed (Figure 2D) reduced CON-PMN connectivity in male *APOE4* carriers occurs between the L_p32pr and R_TPOJ2 (ES: *−0.023*, *p = 7.02E-05*). As most existing literature exhibit more pronounced signals in female *APOE4* carriers for altered brain connectivity and cognitive decline, these findings in male *APOE4* carriers are novel.

Furthermore, using the Yeo-7 atlas, we identified the negative age-*APOE4*-education effects (Figure 2C) on the NFC within the DMN (ES: *−0.019*, *p = 1.0E-3*). In particular, in *APOE4* carriers with high education, the within-DMN NFC decreases faster during aging, indicating that the protective effects of education may diminish in *APOE4* carriers ^91^. No ROI-level associations passed the Bonferroni significance level *0.05/m*; however, two associations exceeded *p < 1E-4* threshold between R_47l and L_d32 (ES: *−0.026*, *p = 2.0E-5*) and between R_47l and L_23d (ES: *−0.025*, *p = 4.9E-5*) (Figure 2D).

### Associations between CVRFs and Brain Functions

**Figure 3.**
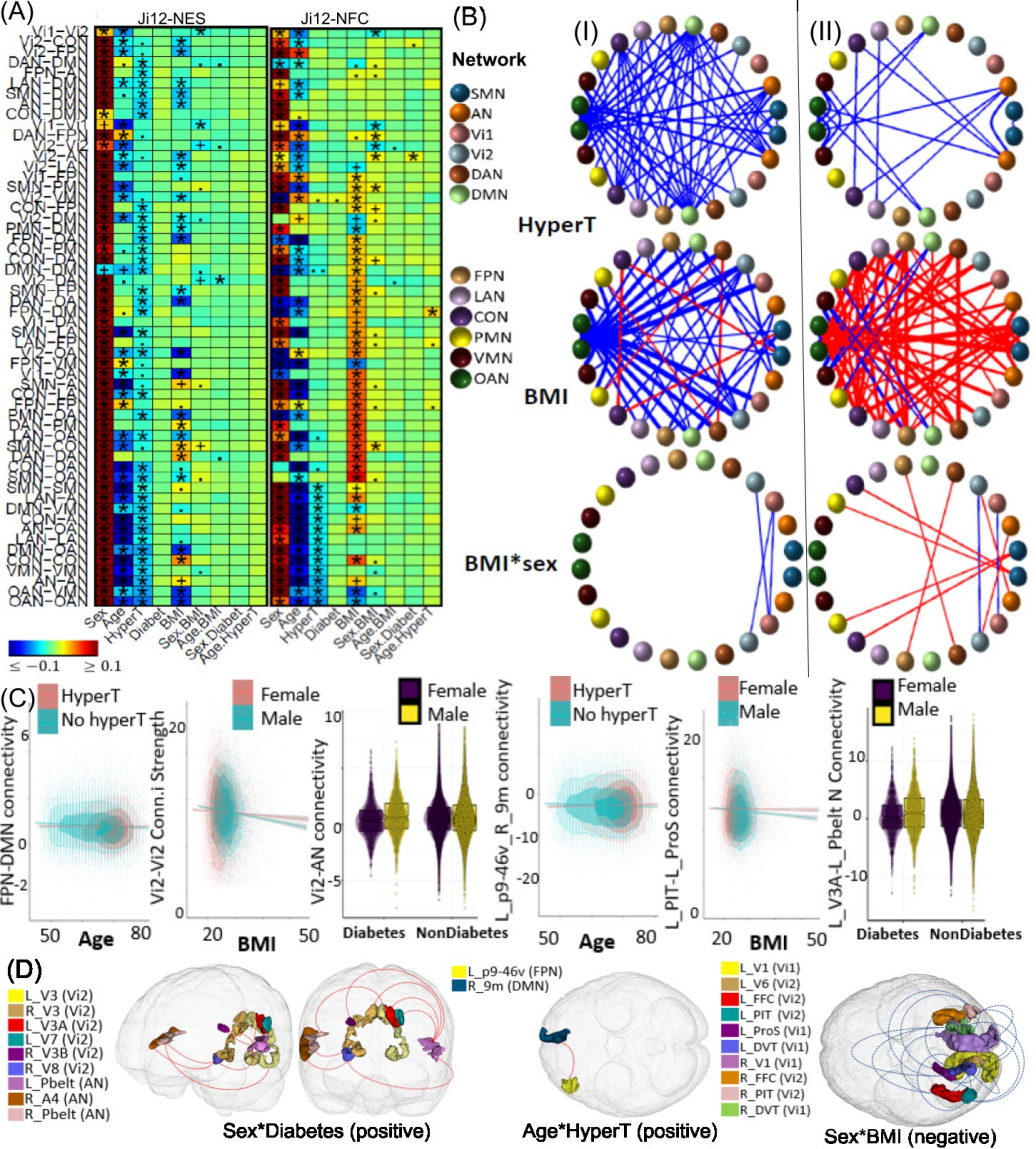
Selected association results on cardiovascular (CVRF) factors (hypertension, BMI and diabetes) and their interactions with age and sex on brain network functional connectivity (NFC) and edge strength (NES) measures. (A) Heatmaps display association results from the white British population using three network atlases: Ji-12, Yeo-7, and Yeo-17, for NES (Panel I) and NFC (Panel II). Results that passed the Bonferroni-corrected significance threshold and were confirmed by the non-British population are marked with (*); significant results not validated by the non-British are indicated by (+). (.) and (..) denote non-significance with p-values less than *1e-4* and *1e-3*, respectively. (B) Circular plots showing association results based on the Ji-12, atlas for the NES in Panel (I) and NFC in Panel (II), respectively. Colors were used to indicate different networks. (C) Scatter plots and boxplots to illustrate CVRF interaction effects. Columns 1–3, age-hypertension interaction effect on FPN-DMN between-network NFC, sex-BMI interaction effect on Vi2-Vi2 with-network NES, and sex-diabetes interaction effect on Vi2-AN between-network NFC, respectively; columns 4–6: age-hypertension interaction effect on the brain functional connectivities (FCs) between brain regions L_p9-46v and R_9m, sex-BMI interaction effect on the FCs between L_PIT and L_ProS, and sex-diabetes interaction effect on the FCs between L_V3A and L_Pbelt connectivity, respectively. (D) Spatial locations of the identified brain regions for the sex-diabetes, age-hypertension, and sex-BMI interactions.

In summary, 33 and 26 main effects of hypertension and BMI on NES measures were identified based on the Ji-12 atlas, with 33 and 24 effects validated by the non-British population, respectively. Additionally, 12 and 40 main effects of hypertension and BMI on NFC measures were observed, with 12 and 32 effects validated by the non-British population. These effects are illustrated in Figures 3A and 3B, with no main effects of diabetes identified. Using the Yeo-7 atlas, 4 and 7 main effects on NES and 7 and 2 on NFC were identified, with 4 and 7 NES effects and 5 and 2 NFC effects validated by the non-British population. Results based on different atlases are listed in Supplementary Table S6, and in Supplementary Figures S2–S7.

Negative effects of hypertension were observed widely across different networks, including within the AN, SMN, OAN, VMN, CON, LAN, and DMN and 26 between-network NES measures (Ji-12), supporting hypertension may accelerate aging ^37,38^. For interactions, we identified the positive age-hypertension effect (Figure 3C and Supplementary Figure S20) on the NFC between the FPN and DMN (ES: *0.029*, *p = 4.9E-6*) networks. In particular, positive age-hypertension effects (Figure 3D and Supplementary Figure S28) were observed on the NFCs between ROIs between L_p9-46v and R_9m (ES: *0.029*, *p = 1.2E-5*), where participants with hypertension exhibited a reduced anti-correlation with aging. This suggests decreased functional segregation of the left dorsolateral and right medial prefrontal cortex in subjects with hypertension. Such reduced anti-correlation may imply a decline in cognitive function, as supported by recent literature indicating that higher blood pressure is causally related to lower brain functional system segregation and worse cognition ^39,40^.

Negative effects of BMI were observed on NES measures within the OAN and 19 between-network pairs. Conversely, positive effects were noted within the DAN and CON, between the SMN and CON, and between the DAN and PMN networks (Figures 3A and 3B, and Supplementary Figures S21–S23). These findings suggest that the impact of BMI on connectivity strength is complex which varies across networks. For interaction effects, we identified the negative sex-BMI interaction effect (Figures 3A and 3C) on the NES within-Vi1 (ES: *−0.023*, *p = 1.2E-4*), and between Vi1 and Vi2 networks (ES: *−0.020*, *p = 6.4E-4*). In particular, negative associations on 19 ROI-level connectivities were identified on the within-Vi1 and between Vi1 and Vi2 networks (Figures 3C and 3D, and Supplementary Figure S29), such as the L_PIT and R_V1. 18 out of 19 (94.7%) associations were validated in nonBritish populations. The sex-BMI interaction effects on visual networks suggested that increased BMI were associated with a more rapid decrease in visual connectivity strength in males compared to females. Besides, positive sex-BMI interaction effects were also observed on the SMN-CON, SMN-PMN, Vi2-AN and DAN-FPN NFC measures. We also identified a negative interaction effect between age and BMI on Vi2-DAN between-network NES, indicating that a higher BMI accelerates the age-related decline in Vi2-DAN network connectivity strength (Supplementary Figure S49).

Although no main effects of diabetes were observed, we identified a positive sex-diabetes interaction effect on the NFCs between the Vi2 and AN networks (ES: *0.021*, *p = 5.7E-4*) (Figure 3C). Specifically, ROI-level analyses revealed positive sex-diabetes interaction effects on FCs between L_PBelt and ROIs L_V3, L_V3A, L_V7, R_V3, R_V8, and R_V3B; between R_PBelt and R_V3, and R_V8; and between R_V8 and R_A4 (Figures 3C and 3D and Supplementary Figure S30). Except for the L_PBelt-L_V3, the positive directions of 88.8% of these associations in the British cohort were validated by the non-British population. These results suggest that females with diabetes experience a greater reduction in visual-auditory connectivity than males, highlighting the greater impact of diabetes on connectivity in females ^41^.

### Associations between Lifestyle Factors and Brain Functions

**Figure 4.**
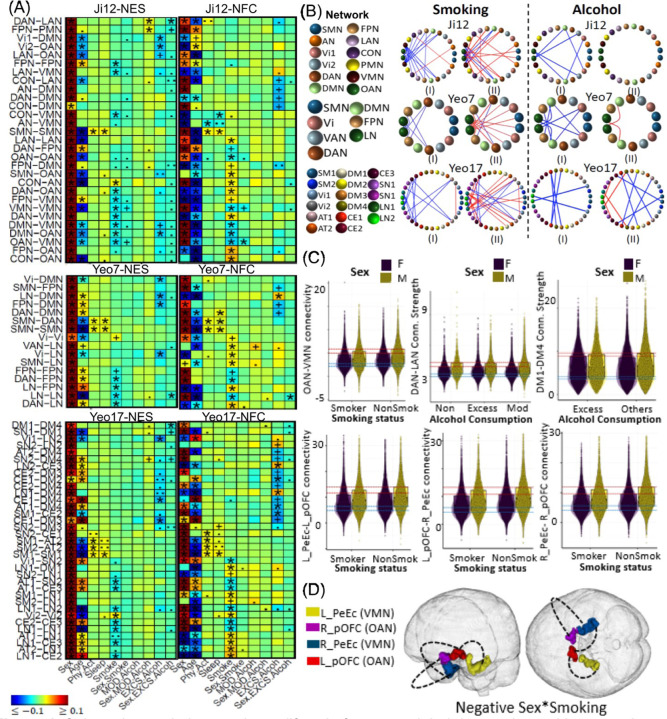
Selected association results on lifestyle factors and their interactions with age and sex on brain network functional connectivity (NFC) and edge strength (NES) measures. (A) Heatmaps display association results from the white British population using three network atlases: Ji-12, Yeo-7, and Yeo-17, for NES (Panel I) and NFC (Panel II). Results that passed the Bonferroni-corrected significance threshold and were confirmed by the non-British population are marked with (*); significant results not validated by the non-British are indicated by (+). (.) and (..) denote non-significance with p-values less than *1e-4* and *1e-3*, respectively. (B) Circular plots showing association results based on three network atlases Ji-12, Yeo-7 and Yeo-17 in three rows for NES in Panel (I) and NFC in (II), respectively. Colors were used to indicate different networks. (C) Boxplots and scatterplots to illustrate the identified smoking- and alcohol-related interaction effects on both the network-level and regional-level connectivity measures. Row 1, columns 1–3: the sex-smoking interaction effect on the OAN-VMN between-network NFC (Ji-12 atlas), sex-excessive alcohol interaction effect on the DAN-LAN between-network NES (Ji-12 atlas), and sex-excessive alcohol interaction effect on the DM1-DM4 between-network NES (Yeo-17 atlas), respectively; row 2, columns 1–3: sex-smoking effects on the brain functional connectivities between brain regions L_PeEc and L_pOFC, between L_pOFC and R_PeEc, and between R_9m and R_pOFC, respectively. (D) Spatial locations of the identified brain regions for the sex*smoking interactions.

In summary, 10, 5, 1 and 1 main effects of smoking, excessive alcohol, physical activity, and sleep on NES measures were identified using the Ji-12 atlas, all validated by the non-British population. Additionally, 15, 4, 2 and 2 main effects of these factors on NFC measures were found, with 12, 0, 2, and 2 validated by the non-British population. Using the Yeo-7 atlas, 6, 4, 2 and 2 main effects on NES and 9, 4, 2, and 3 on NFC were identified, with 4, 4, 2 and 2 on NES and 7, 1, 2 and 3 on NFC validated by the non-British population. Results based on different atlases are illustrated in Figures 4A and 4B, Supplementary Table S6, and in Supplementary Figures S2–S7.

Negative effects of smoking were observed within FPN and VMN, between VMN and FPN, OAN, DAN, DMN, LAN and CON, and between DAN and OAN (Figures 4A and 4B). While the detrimental effects of smoking on reduced brain FCs have been well-documented such as in the DMN, FPN, SAL networks and the lateral orbitofrontal cortex and inferior frontal gyrus ^44–46^, our study was the first to identify the VMN (PeEc and TF regions in inferior medial temporal and lateral temporal cortices) as a network hub significantly affected by smoking. Additionally, a positive effect was found between CON and AN. For interaction effects, we identified the negative sex-smoking effect on the NFC between the VMN and CON (ES: *−0.020*, *p = 2.3E-4*), suggesting males have more serious detrimental effects from smoking (Figures 4A and 4C). Such a negative sex-smoking interaction effect on the NFC between VMN and CON was validated in nonBritish populations (ES: *−0.015*, *p = 0.44*). ROI-level analyses revealed that such sex-disparate smoking detrimental effects occurred on the FCs between the ROIs L_PeEc and L_pOFC, R_PeEc and L_pOFC and R_PeEc and R_pOFC, respectively (Figures 4C and 4D).

Negative effects of excessive alcohol consumption were observed on NES measures between the DMN and OAN, DMN and Vi1, and OAN and Vi2 networks (Figures 4A and 4B). The negative sex-excessive alcohol interaction suggests that excessive alcohol consumption has a more pronounced detrimental effect in males, particularly on the NES between the DAN and LAN (ES: *−0.021*, *p = 1.9E-4*) networks (Figures 4A and 4C and Supplementary Figure S25). In contrast, a positive sex-moderate alcohol interaction was observed on the NES between the DAN and LAN (ES: *0.020*, *p = 4.6E-4*) and between the FPN and PMN (ES: *0.021*, *p = 2.5E-4*) networks. This finding highlights diverging patterns between excessive and moderate alcohol consumption ^92^ in males. No ROI-level associations passed the FDR correction.

Positive effects for both physical activity and sleep were observed within the SMN networks (Figures 4A and 4B). Physical activity is known to facilitate neuroplasticity and enhance brain efficiency, while adequate sleep is essential for memory consolidation and the clearance of brain metabolites, which may help prevent neurodegeneration. The observed benefits on SMN functions align with previous literature, which has linked sensory/somatomotor network connectivity to sleep quality ^60^. No interactions were identified using the Ji-12 and Yeo-7 atlases. However, based on the Yeo-17 atlas, a positive age-sex-sleep three-way interaction term was found between the LN2 and CE3 networks, suggesting that the anticorrelation between LN2 and CE3 networks anticorrelation increases faster during aging in males with < 7 hours of sleep (Supplementary Figure 24).

### Associations between SES and Brain Functions

**Figure 5.**
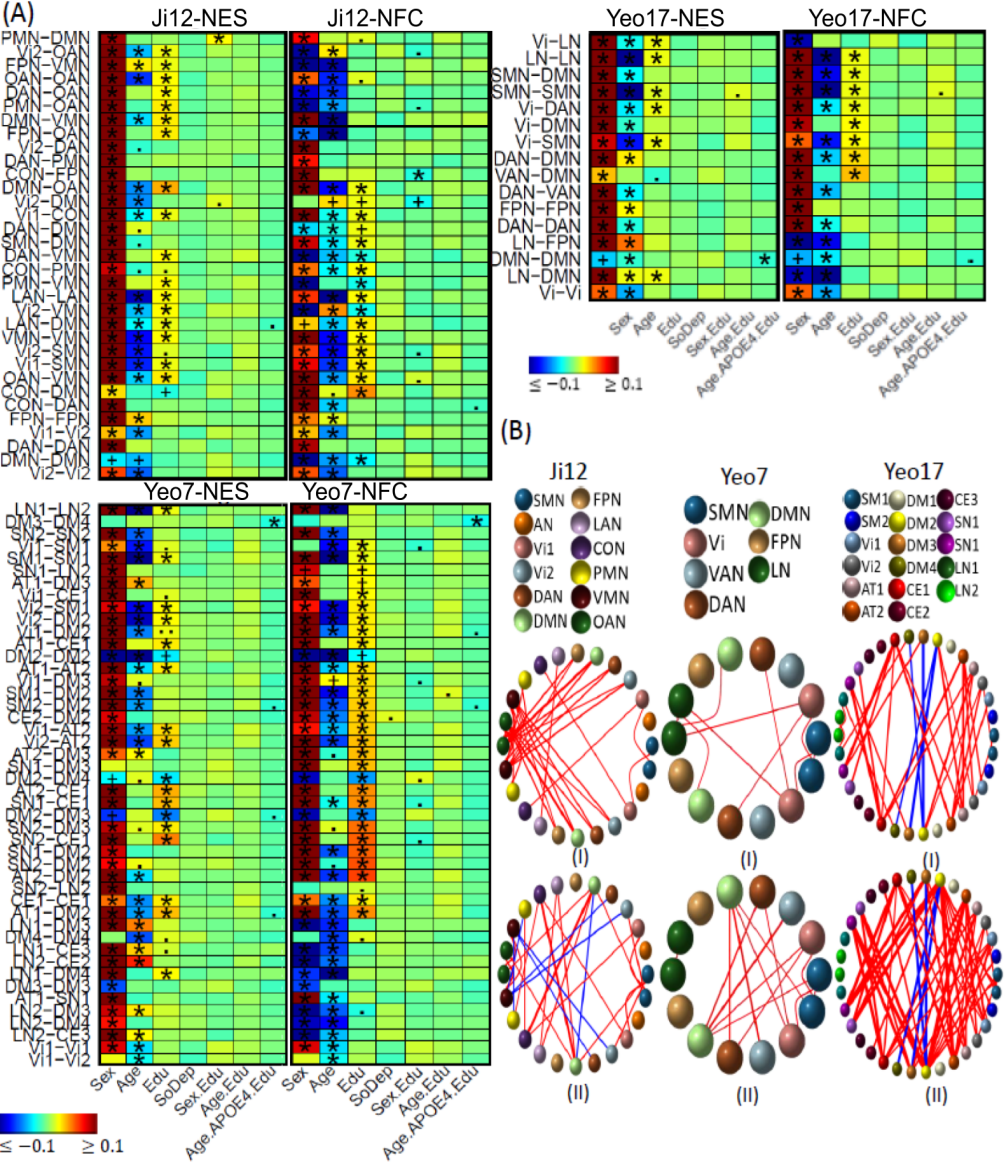
Selected association results on education and their interactions with age and sex on brain network functional connectivity (NFC) and edge strength (NES) measures. (A) Heatmaps display association results from the white British population using three network atlases: Ji-12, Yeo-7, and Yeo-17, for NES (Panel I) and NFC (Panel II). Results that passed the Bonferroni-corrected significance threshold and were confirmed by the non-British population are marked with (*); significant results not validated by the non-British are indicated by (+). (.) and (..) denote non-significance with p-values less than 1e-4 and 1e-3, respectively. (B) Circular plots showing association results between education and NES in panel (I), between education and NFC in panel (II). The results for the three network atlases Ji-12, Yeo-7, and Yeo-17 are shown in columns 1–3 respectively.

No effects of SoDep were identified on NES or NFC measures. Positive effects of education (Figure 5) on the NES measures were observed within OAN, VMN and LAN networks, and across 13 network pairs, including OAN-DAN, OAN-DMN, OAN-FPN, OAN-PMN, OAN-VMN, OAN-Vi2, VMN-PMN, VMN-DAN, VMN-DMN, VMN-FPN, VMN-Vi2, CON-Vi1, and SMN-Vi1. A negative sex-education effect was identified on the NFC between the CON and FPN networks (ES: −0.021, p = 1.7E-4), and a positive sex-education effect was identified on the NES between networks (ES: 0.023, p = 4.5E-5), suggesting that females and males with higher education levels exhibit greater CON-FPN and PMN-DMN connectivities (Supplementary Figures S26 and S27). No ROI-level associations passed the FDR correction.

## Discussion

### A Fine-grained Underpinning of Age, Sex and APOE Effects on the Aging Brain

Our study systematically examined the interactions of aging, sex, and the APOE gene with network connectivity measures. We observed a general decline in both intra- and inter-network NES measures with aging across the majority of networks, highlighting the broad impact of aging on functional connectivity, particularly within the DMN, salience, auditory, attention, and visual networks ^8–11^. However, exceptions were found within the FPN and between the FPN, DAN, and VMN networks, where increased overactivations were observed. This increased frontal activity during aging aligns with the concept of PASA ^10,12,13^, suggesting an increase in frontal activity as posterior occipital activity declines. The increased frontal activity during aging also aligns with the scaffolding mechanism ^93,94^, which posits that older adults recruit compensatory neural resources to maintain cognitive performance.

Our analysis of sex differences in network connectivity measures revealed distinct patterns that extend previous findings. Males exhibited higher NES measures across the majority of networks, except within the DMN network, where females demonstrated significantly higher connectivity ^88,89^. For age-sex interactions, we found that males experienced a larger increase in NESs within the FPN and between DMN-LN and DAN-LN networks, along with a greater reduction within the visual network. This suggests a substantial neural reorganization, potentially involving compensatory or the PASA mechanisms, occurring more prominently in males than in females. In contrast, females showed greater reductions in connectivity strength between AN, DMN, VMN, and CON networks. The pronounced decline in these networks in females might indicate a higher vulnerability to neurodegenerative processes ^95^. Overall, this highlights distinct patterns of network connectivity and compensatory mechanisms in aging males and females, with males showing more extensive reorganization and females facing greater challenges from neurodegenerative conditions.

For the *APOE* gene, the negative impacts of the APOE4 allele on key cognitive and visual networks highlight its role in neural integrity deterioration ^17^. Specifically, the negative sex-APOE4 interaction effect on NES between the CON and PMN networks underscores the vulnerability of male APOE4 carriers during aging. While most existing literature has focused on the cognitive vulnerability of female APOE4 carriers ^3,22,25^—often attributed to greater hypometabolism, atrophy, and reduced DMN connectivity—there is limited research on the atrophy of brain networks in males. Interestingly, some studies have observed that APOE4’s effects are more pronounced in the longitudinal decline of memory and executive function in men, whereas in women, these effects are more evident in the decline of attention ^24^. Our findings revealed a negative sex-APOE4 interaction, indicating that male APOE4 carriers may experience more reduced connectivity in the L_p32pr-R_TPOJ2 region and between the CON and PMN networks. These areas and networks are critically involved in memory and executive function. The CON-PMN network connectivity is essential for complex cognitive tasks that require the maintenance of task goals and the integration of diverse sensory and cognitive information. The L_p32pr region, part of the dorsal medial prefrontal cortex, is crucial for high-level executive functions such as decision-making, emotional regulation, and social cognition. Meanwhile, the R_TPOJ2 region, located at the intersection of the temporal, parietal, and occipital lobes, plays a vital role in processing social information, integrating sensory inputs, and understanding others’ perspectives or predicting their thoughts and intentions.

Moreover, our finding of negative age-*APOE4*-education interaction effects within the DMN suggest that the protective effects of education are diminished in *APOE4* carriers ^91^, indicating that higher educational attainment may not fully mitigate the adverse effects of aging on brain connectivity in *APOE4* carriers.

### CVRFs are Extensively Associated with Brain Functional Changes

The current investigation provides compelling evidence linking cardiovascular risk factors—specifically hypertension, BMI, diabetes, smoking and alcohol—with significant alterations in brain FC. These findings highlight the complex interplay between cardiovascular health and brain functional networks across various brain regions.

Elevated blood pressure, a prevalent modifiable risk factor for cardiovascular diseases, is linked to cognitive decline in late life. Hypertension predominantly exhibited negative effects on connectivity within and between multiple networks, including the attentional, SMN, and DMN. This widespread disruption may be attributed to hypertension’s contribution to neural inefficiencies across a broad spectrum of cognitive and sensory processes ^96,97^. In addition, we observed a reduced anti-correlation between L_p9-46v (dorsolateral prefrontal cortex) and R_9m (medial prefrontal cortex), indicating decreased functional segregation of these regions. In a healthy brain, anti-correlation reflects balanced and efficient network activity, where the activity of one region inhibits the other. Reduced anti-correlation suggests a decline in this balance, leading to less efficient information processing and integration, often associated with cognitive decline and impaired executive function, which are common in aging and exacerbated by hypertension. A recent study ^39^ validated this finding, demonstrating that higher blood pressure is causally related to lower brain functional segregation, resulting in worse cognition in the general aging population through observational and Mendelian randomization analyses.

The influence of BMI on brain function revealed a mixed pattern. Negative effects predominated within the OAN and 19 between-network pairs, and positive effects within the DAN and CON, between the SMN and CON, and between the DAN and PMN networks. Similar findings ^49^ have reported decreased functional connectivity in the ventromedial and ventrolateral prefrontal cortex, insula, and caudate nucleus during rest and milkshake consumption, while increases were noted in regions associated with the DAN connectivity. The sex-BMI interaction effects underscore the larger impact of obesity on brain function in males compared to females. Specifically, our results indicated a stronger negative effect of BMI on visual network connectivity strength in males, along with positive effects on between-network connectivity related to sensorimotor, attention, and executive control functions (SMN-CON, SMN-PMN, Vi2-AN, and DAN-FPN). This finding corroborates previous research, indicating that men experience detrimental changes in brain connectivity starting from the overweight category, whereas women do not show obvious declines until the obese range, possibly due to obesity-induced chronic white matter damage in males ^98^.

We identified a sex-diabetes interaction effect that enhances the FC between the visual and auditory networks. This positive association was particularly evident between multiple ROIs within these networks, such as L_PBelt and L_V3. These results suggest that diabetes may impact brain connectivity differently based on sex, with females showing a greater change in visual-auditory connectivity ^41^.

Our findings on the impact of smoking highlight the detrimental effects of smoking on brain health. We observed negative effects within and between the FPN, VMN, OAN, DAN, DMN, LAN, and CON, consistent with studies ^44,45^ showing broad neurotoxic effects of nicotine and other smoking-related toxins on brain functional networks. In addition, we found a negative sex-smoking effect on connectivity between the VMN and CON, suggesting more severe smoking-related VMN-CON impairments in males.

Turning to the effects of alcohol consumption, our study differentiates between the impacts of excessive and moderate alcohol intake. We found that excessive alcohol consumption predominantly harms connectivity within networks associated with higher cognitive functions and emotional processing, such as the DMN and OAN, as well as between these networks and visual processing areas (Vi1 and Vi2). These findings corroborate with studies suggesting that alcohol neurotoxicity may disrupt the neural substrate underlying both cognitive and emotional regulation. It was found ^50^ that reduced connectivities related to precuneus, postcentral gyrus, insula and visual cortex were the main brain areas with rfMRI NC reduction suggesting reduced interoceptive awareness in drinkers; in addition, reduced left executive control network FC ^51^ is associated with alcohol use disorders. Research has shown that males with alcohol misuse have lower cortical volume and thickness, reduced white matter volume and hippocampal volume, and greater changes in brain function and behavior ^99–103^. Interestingly, while excessive alcohol intake has detrimental effects in males, we observed that moderate alcohol consumption had a positive sex-moderate alcohol interaction was observed on the NES between the DAN and LAN and between the FPN and PMN network, suggesting a protective influence on connectivity measures between the dorsal attention, language and frontoparietal networks. This dichotomy highlights the complex relationship between alcohol dosage and brain health, suggesting that moderate consumption might support certain aspects of cognitive connectivity ^92^.

### Healthy Lifestyle and Education Benefit Brain Functionality

The current investigation elucidates the multifaceted roles of healthy lifestyle factors, including education, physical activity, and sleep, and their complex interactions with sex, age and *APOE* gene on brain FC. By analyzing their effects across various neural networks, our findings highlight the significance of maintaining a healthy lifestyle in enhancing brain health.

Our study revealed significant positive associations of education with connectivity within and between several major brain functional networks, including the OAN, VMN, LAN and FPN. Higher educational attainment was linked to increased brain network connectivity, specifically, between regions such as the anterior cingulate cortex and the hippocampus, inferior frontal lobe, posterior cingulate cortex, and angular gyrus ^53^, potentially enhancing cognitive reserve and maintaining functionality during aging ^54^. Additionally, we observed a negative and a positive sex-education effect on the CON-FPN NFC and PMN-DMN NES, indicating that education benefits different network pairs across sexes. Specifically, more pronounced positive effects on the CON-FPN and PMN-DMN networks were seen in females and males, respectively. The differential impact of education on these networks suggests that educational experiences may enhance neural pathways that align with the cognitive strengths or needs of each sex. For females, the emphasis may be on bolstering executive functions and cognitive control, which are crucial for managing complex tasks and responsibilities. For males, education may primarily enhance the networks involved in integrating complex information and memory processes, supporting tasks that require strategic thinking and planning. The sex-specific effect of education on brain FCs suggests the potential for tailored educational programs to optimize brain health and cognitive function.

Turning to physical activity and sleep, our findings underscore their vital role in maintaining connectivity within the SMN. The positive association between these lifestyle factors and brain function supports a wide array of research advocating for the neuroprotective effects of regular physical activity and sufficient sleep. These benefits also align with the active framework theory ^104^, which posits that these lifestyle factors benefit cerebral health by improving vascular function and reducing inflammatory markers, and in turn supports neurocognitive function.

### Limitations and Future Work

Leveraging the large-sample fMRI data from the UKB, we were able to investigate a wide range of modifiable and non-modifiable risk factors and explored their joint, conditional and interaction effects on brain functions, which were validated by multi-atlases and multi-ethnic populations. This ensured the robustness of our results against potential racial differences, atlas choices, outlier sensitivities and sample size limitations. As the UKB prepares to include MR images from 100,000 subjects, we will continually enrich our resources and analyses with new data and parcellation schemes, including those from Gordon ^105^, Power ^106^, and various ICA atlases ^64,65,107^. Additionally, we plan to extend our genetic analyses from the *APOE4* gene to polygenic risk scores, high-dimensional single nucleotide polymorphisms, and other genomics data. We will also assess the generalizability of gene-environment interactions from UKB data to multi-site fMRI data across the lifespan ^108^, such as the Adolescent Brain Cognitive Development (ABCD) ^109^ study. Furthermore, we plan to explore the impacts of different preprocessing approaches, such as surface-based registration ^110^, to enhance registration accuracy, adherence to the inherent geometry of cortical surfaces, reliability of connectivity metrics, and statistical power ^111,112^.

Our main analyses were based on parcellation-based full correlations. Although FMRIB’s ICA-based X-noiseifier (FIX) has been applied to the UKB dataset to remove scanner artifacts and motion effects, full correlation measures can be more sensitive to remaining global artifacts and noises than partial correlations ^66,113^. To address this, global artifacts can be further reduced by measuring partial functional connectivity between paired brain regions, removing dependencies on other brain regions ^114,115^. Future studies should explore parcellation-based partial correlation traits for a large number of parcels with a limited number of time points in the UKB study.

## Supplementary Material

Supplement 1

## Figures and Tables

**Table 1.** Demographic information, lifestyle factors, socioeconomic status (SES) and cardiovascular risk factors (CVRFs) of the 36,630 UK Biobank subjects.

Characteristic	Number(%)		
	Female (N=19395)	Male (N=17235)	Total (N=36630)
Age at brain imaging			
Mean(sd)	63.0 (7.39)	64.3 (7.65)	63.6 (7.54)
Median(range)	63.0 [45.0, 81.0]	65.0 [44.0, 81.0]	64.0 [44.0, 81.0]
Ethnicity			
British	17861 (92.1%)	15963 (92.6%)	33824 (92.3%)
Others	1534 (7.9%)	1272 (7.4%)	2806 (7.7%)
APOE4			
0 APOE4	13868 (71.5%)	12576 (73.0%)	26444 (72.2%)
1 APOE4	5085 (26.2%)	4295 (24.9%)	9380 (25.6%)
2 APOE4	442 (2.3%)	364 (2.1%)	806 (2.2%)
APOE2			
0 APOE2	16378 (84.4%)	14619 (84.8%)	30997 (84.6%)
1 APOE2	2904 (15.0%)	2518 (14.6%)	5422 (14.8%)
2 APOE2	113 (0.6%)	98 (0.6%)	211 (0.6%)
Marriage status			
Living with a partner	13619 (70.2%)	13913 (80.7%)	27532 (75.2%)
Not living with a partner	1235 (6.4%)	520 (3.0%)	1755 (4.8%)
Education			
College/university degree or above	8626 (44.5%)	8293 (48.1%)	16919 (46.2%)
No college/university degree or above	9433 (48.6%)	7737 (44.9%)	17170 (46.9%)
Social Deprivation			
Above the Townsend deprivation index median	8275 (42.7%)	7092 (41.1%)	15367 (42.0%)
Below the Townsend deprivation index median	11099 (57.2%)	10130 (58.8%)	21229 (58.0%)
Lifestyle factors			
Smoking status			
Ever smoked	12459 (64.2%)	9867 (57.2%)	22326 (61.0%)
Never invoked	6936 (35.8%)	7368 (42.8%)	14304 (39.1%)
Doing regular physical activity			
Yes	14199 (73.2%)	13267 (77.0%)	27466 (75.0%)
No	5040 (26.0%)	3893 (22.6%)	8933 (24.4%)
Healthy diet			
Yes	10517 (54.2%)	7371 (42.8%)	17888 (48.8%)
No	8870 (45.7%)	9854 (57.2%)	18724 (51.1%)
Alcohol consumption			
Excessive alcohol consumption	2742 (14.1%)	1284 (7.5%)	4026 (11.0%)
Moderate alcohol consumption	11115 (57.3%)	11096 (64.4%)	22211 (60.6%)
Not current drinking	5531 (28.5%)	4848 (28.1%)	10379 (28.3%)
Sleeping between 6 hours and 8 hours			
Yes	8193 (42.2%)	7759 (45.0%)	15952 (43.5%)
No	11145 (57.5%)	9455 (54.9%)	20600 (56.2%)
CVRFs factors			
Hypertension Status			
Yes	3060 (15.8%)	4259 (24.7%)	7319 (20.0%)
No	16335 (84.2%)	12976 (75.3%)	29311 (80.0%)
Diabetes			
Yes	573 (3.0%)	975 (5.7%)	1548 (4.2%)
No	18822 (97.0%)	16260 (94.3%)	35082 (95.8%)
BMI			
Mean(sd)	26.0 (4.53)	27.1 (3.73)	26.5 (4.20)
Median (range)	25.2 [14.7, 56.6]	26.7 [16.7, 56.0]	26.0 [14.7, 56.6]
