# Supplementary material for: The Interaction Effects of Age, Sex, APOE and Common Health Risk Factors on Human Brain Functions
Source: medRxiv. 2024 Aug 5:2024.08.05.24311482. Preprint. [Version 1] doi: 10.1101/2024.08.05.24311482 (PMC11326347; doi:10.1101/2024.08.05.24311482)
Supplement: Supplementary file 1 [file media-1.pdf]

Supplementary Materials for

The Interaction Effects of Age, Sex, APOE and Common Health Risk Factors on Human Brain Functions

Tengfei Li[1,2,†], Jie Chen[3†], Bingxin Zhao[4], Hui Chen[5], Changzheng Yuan[5,6], Gwenn A. Garden[7], Kelly S. Giovanello[8], Guorong Wu[8,9,10,11,*], and Hongtu Zhu[1,3,9,12,*]

[1]Biomedical Research Imaging Center, School of Medicine, University of North Carolina at Chapel Hill, Chapel Hill, NC, USA
[2]Department of Radiology, School of Medicine, University of North Carolina at Chapel Hill, Chapel Hill, NC, USA
[3]Department of Biostatistics, University of North Carolina at Chapel Hill, Chapel Hill, NC, USA
[4]Department of Statistics and Data Science, the Wharton School, University of Pennsylvania, Philadelphia, PA, USA
[5]School of Public Health, Zhejiang University School of Medicine, Hangzhou, China
[6]Department of Nutrition, Harvard T H Chan School of Public Health, Boston, MA, USA
[7]Department of Neurology, School of Medicine, University of North Carolina at Chapel Hill, Chapel Hill, NC, USA
[8]Department of Psychiatry, School of Medicine, University of North Carolina at Chapel Hill, Chapel Hill, NC, USA
[9]Departments of Statistics and Computer Science, University of North Carolina at Chapel Hill, Chapel Hill, NC, USA
[10]UNC Neuroscience Center, University of North Carolina at Chapel Hill, Chapel Hill, NC, USA
[11]Carolina Insititute for Developmental Disabilities, Chapel Hill, NC, USA
[12]Departments of Genetics, University of North Carolina at Chapel Hill, Chapel Hill, NC, USA
[†] *These authors contributed equally*

**Corresponding author*
Hongtu Zhu
Professor
Department of Biostatistics
3105C McGavran_Greenberg Hall, CB# 7420
The University of North Carolina at Chapel Hill
Chapel Hill, NC 27599
919-966-7250
Email : htzhu@email.unc.edu
Guorong Wu
Associate Professor
Department of Psychiatry
The University of North Carolina at Chapel Hill
Chapel Hill, NC 27599
984-974-5217
Email: guorong_wu@med.unc.edu

The Supplementary Materials include the following items:

Supplementary Tables S1-S6

Supplementary Figures S1-S49

Tables

Variable	UK Biobank field I	Defination	Details
Education	6138	2 categories:<br>(1) Having college or university degree<br>(0) Not having college or univerisity degree	A levels/AS levels, O levels/GCSEs, CSEs, NVQ, HND, HNC or other professional qualifications, such as nursing, teaching and their equivalents are considered as not having college or univeristy degree
Social deprivation	189	2 catergories:<br>(1) Above median townsend deprivation index<br>(0) Below median townsend deprivation index	Median is caculated from the 500K UKB participants
Doing regular physical activity	884, 894, 904, 914	2 catergories:<br>(1) Doing regular physical activity<br>(0) Not doing regular physical activity	According to the AHA recommendations, meeting any of the 5 criteria is considered as doing regular physical activity:<br>1. Doing more than 150 minutes of moderate activity each week<br>2. Doing more than 75 minutes of rigorous activity each week<br>3. Equivalent combinations of moderate and rigorous activity (2 minutes of moderate eqauls 1 minutes of rigorous activity)<br>4. Doing moderate activity at least 5 days a week<br>5. Doing rigourous activity at least 5 days a week
Healthy diet	1309, 1319, 1289, 1299, 1329, 1339, 1349, 1369, 1379, 1389, 1438, 1448, 1458, 1468	2 catergories:<br>(1) Meeting at least 4 of the 7 healthy eating criteria<br>(0) Only meeting 3 or less of the 7 healthy eating criteria	7 healthy eating criteria:<br>1. Eating >= 3 servings of fruits a day<br>2. Eating >= 3 servings of vegetables a day<br>3. Eating >= 2 servings of vegetables a week<br>4. Eating >= 3 servings of whole grains a day<br>5. Eating <= 1.5 servings of refined grains a day<br>6. Eating <= 1 serving of processed meats a week<br>7. Eating <= 1.5 servings of unprocessed red meats a week
Alcohol consumption	20117, 1558, 1568, 1578, 1588, 1598, 1608, 5364, 4407, 4418, 4429, 4440, 4451, 4462	3 catergories:<br>(2) Drinking excessive alcohol<br>(1) Drinking moderate alcohol<br>(0) Not drinking at all	According to the 2015-2020 US dietary guidelines for American, up to 1 drink a day for women and up to 2 drinks a day for men are considered drinking moderate alcohol. More than the above threshold is considered drinking excessive alcohol.<br>14g pure alcohol is considered 1 drink, 1 glass of wine (125ml) is equivalent to 0.85 drink, 1 glass of fortified wine (50ml) is equivalent to 0.56 drink, 1 shot of spirits (25ml) is equivalent to 0.57 drink and 1 pint of 4% ABV beer is equivalent to 1.28 drink.
Sleeping between 6 hours and 8 ho	1160	2 catergories:<br>(1) Sleeping between 6 hours and 8 hours a day<br>(0) Sleeping less than 6 hours a day or sleeping longer than 8 hours a day	According to previous studies, sleeping too long or too short are both associated with higher risks of dementia
Smoking Status	20160	2 catergories:<br>(1) Ever had smoked<br>(0) Never smoked	(1) include ever-smokers and never-smokers
Hypertension Status	20002	2 catergories:<br>(1) Having hypertension<br>(0) Never expreienced hypertension	Including essential hypertension4895 and gestational hypertension/pre-eclampsia
Diabete	120007	2 catergories:<br>(1) Ever had Diabete<br>(0) Never had Diabete	Including both Type I and Type II
BMI	21001	Continuous variable with mean around 27	Units of measurement are Kg/m2.

Supplementary Table S1. Criteria for the SES variables, CVRFs and lifestyle factors generated from the UKB data fields. All variables are measured at the baseline.

Models Investigated	Terms	Models
(M0) Main effect model	Age,Sex,APOE4,APOE2,Smoking_status,hyperten_status,Diabete,BMI,Edu,SoDep,Regular_physical_activity,Healthy_diet,Sleep_7,MOD.alcohol,EXCS.alcohol	Sex, Age) fMRI ~ Sex + Age+ APOE4+APOE2+SES + Lifestyle +CVRFs+head_motion1+head_motion2+brain_position1+brain_position2+brain_position3+brain_position4+volumetric_scaling+Partner+UKB_Phase1+UKB_Phase2;<br>All others) fMRI ~ APOE4+APOE2+SES + Lifestyle + CVRFs + head_motion1+head_motion2+brain_position1+brain_position2+brain_position3+brain_position4+volumetric_scaling+Partner+UKB_Phase1+UKB_Phase2+Age+Sex+Age:Sex+(Age^2)+(Age^2):Sex
(M1) Two-way effect model	Age:Smoking_status,Age:hyperten_status,Age:Diabete,Age:BMI,Age:Edu,Age:SoDep,Age:Regular_physical_activity,Age:Healthy_diet,Age:Sleep_7,Age:MOD.alcohol,Age:EXCS.alcohol,Sex:Smoking_status,Sex:hyperten_status,Sex:Diabete,Sex:BMI,Sex:Edu,Sex:SoDep,Sex:Regular_physical_activity,Sex:Healthy_diet,Sex:Sleep_7,Sex:MOD.alcohol,Sex:EXCS.alcohol,APOE4:Smoking_status,APOE4:hyperten_status,APOE4:Diabete,APOE4:BMI,APOE4:Edu,APOE4:SoDep,APOE4:Regular_physical_activity,APOE4:Healthy_diet,APOE4:Sleep_7,APOE4:MOD.alcohol,APOE4:EXCS.alcohol,APOE2:Smoking_status,APOE2:hyperten_status,APOE2:Diabete,APOE2:BMI,APOE2:Edu,APOE2:SoDep, APOE2:Regular_physical_activity,APOE2:Healthy_diet,APOE2:Sleep_7,APOE2:MOD.alcohol, APOE2:EXCS.alcohol,Age:Sex,Sex:APOE4,Age:APOE4,Sex:APOE2,Age:APOE2	Sex*Age) fMRI ~ Sex*Age + APOE4+APOE2+SES + Lifestyle +CVRFs+head_motion1+head_motion2+brain_position1+brain_position2+brain_position3+brain_position4+volumetric_scaling+Partner+site1+site2;<br>All others) fMRI ~ A:B + APOE4+APOE2+SES + Lifestyle +CVRFs +head_motion1+head_motion2+brain_position1+brain_position2+brain_position3+brain_position4+volumetric_scaling+Partner+UKB_Phase1+UKB_Phase2+Age:Sex+(Age^2):Sex, where A and B, the two factors in the interaction term of interest, loops over all APOE, lifestyle, CVRF, and SES covariates.
(M2) Three-way effect model	Age:Sex:Smoking_status,Age:Sex:hyperten_status,Age:Sex:Diabete,Age:Sex:BMI,Age:Sex:Edu,Age:Sex:SoDep,Age:Sex:Regular_physical_activity,Age:Sex:Healthy_diet,Age:Sex:Sleep_7,Age:Sex:MOD.alcohol,Age:Sex:EXCS.alcohol,Sex:APOE4:Smoking_status,Sex:APOE4:hyperten_status,Sex:APOE4:Diabete,Sex:APOE4:BMI,Sex:APOE4:Edu,Sex:APOE4:SoDep,Sex:APOE4:Regular_physical_activity,Sex:APOE4:Healthy_diet,Sex:APOE4:Sleep_7,Sex:APOE4:MOD.alcohol,Sex:APOE4:EXCS.alcohol,Age:APOE4:Smoking_status,Age:APOE4:hyperten_status,Age:APOE4:Diabete,Age:APOE4:BMI,Age:APOE4:Edu,Age:APOE4:SoDep,Age:APOE4:Regular_physical_activity,Age:APOE4:Healthy_diet,Age:APOE4:Sleep_7,Age:APOE4:MOD.alcohol,Age:APOE4:EXCS.alcohol,Age:Sex:APOE4,Age:Sex:APOE2	fMRI ~ A:B:C+APOE4+APOE2+SES + Lifestyle +CVRFs+ head_motion1+head_motion2+brain_position1+brain_position2+brain_position3+brain_position4+volumetric_scaling+Partner+UKB_Phase1+UKB_Phase2+Age:Sex+(Age^2):Sex, where A, B and C, the three factors in the interaction term of interest, loops over all APOE, lifestyle, CVRF, and SES covariates.

Supplementary Table S2. Models investigated in this study. Each interaction term is tested separately, while controlling main effects of all risk factors as confounding factors. A three-way interaction term implied that all its relevant two-way interactions and main effects were also included; a two-way interaction term implied that all relevant main effects were also included. “A:B” denotes the interaction of covariates A and B; “A:B:C" denotes the three-way interaction of covariates A, B, and C. PRS, polygenic risk score; ROI, regions of interest; SES, socioeconomic status; CVRFs, cardiovascular risk factors.

FC Measures	Network	Phenotype	Number of connectivities *m*	Number of Signals	Number of Signals validated by non-British
Ji12-RestConn	Ji-12	NFC	66 (between) + 12 (within) =78	113	91 (80.5%)
Ji12-Abs-RestConn	Ji-12	NES	66 (between) + 12 (within) =78	113	102 (90.3%)
Yeo7-RestConn	Yeo-7	NFC	21 (between) + 7 (within) =28	68	55 (80.9%)
Yeo7-Abs-RestConn	Yeo-7	NES	21 (between) + 7 (within) =28	54	49 (90.7%)
Yeo17-RestConn	Yeo-17	NFC	136 (between) + 17 (within) =153	185	154 (83.2%)
Yeo17-Abs-RestConn	Yeo-17	NES	136 (between) + 17 (within) =153	123	110 (89.4%)

Supplementary Table S3. All the functional connectivity metrics tested in our paper are presented with basic information, including the number of signals (sex, age and sex*age not included; associations that pass the corresponding Bonferroni significance level 0.05/*m* in white British) and the number of signals that were validated by non-British populations (with the same directions as in white British). NFC: network-mean functional connectivity; NES: network connectivity strength (network-mean absolute functional connectivity).

Characteristic	Number(%)						
	APOE4 homozygote (N=760)	APOE3 homozygote (N=19943)	APOE2 homozygote (N=196)	APOE4/APOE3 (N=7885)	APOE2/APOE3 (N=4209)	APOE4/APOE2 (N=831)	Total (N=33824)
Age at brain imaging							
Mean(sd)	63.2 (7.24)	63.9 (7.51)	63.4 (7.85)	63.4 (7.46)	64.0 (7.47)	63.9 (7.58)	63.8 (7.49)
Median(range)	64.0 [48.0, 80.0]	65.0 [45.0, 81.0]	63.5 [46.0, 80.0]	64.0 [46.0, 81.0]	65.0 [46.0, 81.0]	64.0 [47.0, 80.0]	64.0 [45.0, 81.0]
Sex							
Female	419 (55.1%)	10402 (52.2%)	103 (52.6%)	4244 (53.8%)	2234 (53.1%)	459 (55.2%)	17861 (52.8%)
Male	341 (44.9%)	9541 (47.8%)	93 (47.4%)	3641 (46.2%)	1975 (46.9%)	372 (44.8%)	15963 (47.2%)
Marriage status							
Living with a partner	587 (77.2%)	15104 (75.7%)	151 (77.0%)	5959 (75.6%)	3186 (75.7%)	614 (73.9%)	25601 (75.7%)
Not living with a partner	32 (4.2%)	885 (4.4%)	12 (6.1%)	382 (4.8%)	176 (4.2%)	48 (5.8%)	1535 (4.5%)
Education							
College/university degree or above	360 (47.4%)	9011 (45.2%)	82 (41.8%)	3537 (44.9%)	1878 (44.6%)	389 (46.8%)	15257 (45.1%)
No college/university degree or above	355 (46.7%)	9493 (47.6%)	100 (51.0%)	3823 (48.5%)	2015 (47.9%)	378 (45.5%)	16164 (47.8%)
Social Deprivation							
Above the Townsend deprivation index median	291 (38.3%)	8164 (40.9%)	74 (37.8%)	3248 (41.2%)	1647 (39.1%)	325 (39.1%)	13749 (40.6%)
Below the Townsend deprivation index median	469 (61.7%)	11762 (59.0%)	122 (62.2%)	4631 (58.7%)	2557 (60.8%)	506 (60.9%)	20047 (59.3%)
Lifestyle factors							
Smoking status							
Ever smoked	467 (61.4%)	12201 (61.2%)	125 (63.8%)	4822 (61.2%)	2550 (60.6%)	536 (64.5%)	20701 (61.2%)
Never smoked	293 (38.6%)	7742 (38.8%)	71 (36.2%)	3063 (38.8%)	1659 (39.4%)	295 (35.5%)	13123 (38.8%)
Doing regular physical activity							
Yes	575 (75.7%)	14964 (75.0%)	144 (73.5%)	5869 (74.4%)	3174 (75.4%)	604 (72.7%)	25330 (74.9%)
No	183 (24.1%)	4861 (24.4%)	52 (26.5%)	1962 (24.9%)	1015 (24.1%)	221 (26.6%)	8294 (24.5%)
Healthy diet							
Yes	397 (52.2%)	9693 (48.6%)	86 (43.9%)	3896 (49.4%)	1997 (47.4%)	397 (47.8%)	16466 (48.7%)
No	363 (47.8%)	10247 (51.4%)	110 (56.1%)	3985 (50.5%)	2211 (52.5%)	434 (52.2%)	17350 (51.3%)
Alcohol consumption							
Excessive alcohol consumption	75 (9.9%)	1989 (10.0%)	27 (13.8%)	826 (10.5%)	433 (10.3%)	88 (10.6%)	3438 (10.2%)
Moderate alcohol consumption	478 (62.9%)	12213 (61.2%)	112 (57.1%)	4743 (60.2%)	2557 (60.8%)	525 (63.2%)	20628 (61.0%)
Not current drinking	207 (27.2%)	5735 (28.8%)	57 (29.1%)	2315 (29.4%)	1218 (28.9%)	218 (26.2%)	9750 (28.8%)
Sleeping between 6 hours and 8 hours							
Yes	338 (44.5%)	8640 (43.3%)	89 (45.4%)	3535 (44.8%)	1754 (41.7%)	380 (45.7%)	14736 (43.6%)
No	420 (55.3%)	11265 (56.5%)	107 (54.6%)	4339 (55.0%)	2442 (58.0%)	449 (54.0%)	19022 (56.2%)
CVRFs factors							
Hypertension Status							
Yes	148 (19.5%)	4092 (20.5%)	55 (28.1%)	1487 (18.9%)	826 (19.6%)	160 (19.3%)	6768 (20.0%)
No	612 (80.5%)	15851 (79.5%)	141 (71.9%)	6398 (81.1%)	3383 (80.4%)	671 (80.7%)	27056 (80.0%)
Diabetes							
Yes	23 (3.0%)	863 (4.3%)	8 (4.1%)	274 (3.5%)	180 (4.3%)	42 (5.1%)	1390 (4.1%)
No	737 (97.0%)	19080 (95.7%)	188 (95.9%)	7611 (96.5%)	4029 (95.7%)	789 (94.9%)	32434 (95.9%)
BMI							
Mean(sd)	26.2 (3.88)	26.5 (4.18)	26.8 (4.72)	26.6 (4.23)	26.7 (4.22)	26.7 (4.39)	26.6 (4.20)
Median(range)	25.9 [16.4, 44.4]	26.0 [15.2, 56.6]	26.2 [18.0, 45.3]	26.0 [14.7, 56.0]	26.2 [16.2, 50.5]	25.9 [18.0, 44.7]	26.0 [14.7, 56.6]

*: Percentiles do not add up to 1 is due to missingness.

*:All variables are measured at the baseline except the age at imaging, hypertension status, and diabetes.

Supplementary Table S4 Demographic information, lifestyle factors and cardiovascular risk factors of the white British UKB subjects used in the association study on fMRI connectivities. Percentiles may not add up to 1 due to missingness. All variables were measured at baseline, with the exception of age at imaging, diabetes status, and hypertension status.

Characteristic	Number(%) APOE4 homozygote (N=46)	APOE3 homozygote (N=1745)	APOE2 homozygote (N=15)	APOE4/APOE3 (N=618)	APOE2/APOE3 (N=336)	APOE4/APOE2 (N=46)	Total (N=2806)
Age at brain imaging							
Mean(sd)	60.9 (7.63)	61.9 (7.89)	62.7 (9.18)	61.6 (7.65)	62.0 (8.13)	61.5 (7.81)	61.8 (7.86)
Median(range)	60.5 [48.0, 79.0]	62.0 [44.0, 81.0]	63.0 [48.0, 78.0]	61.0 [47.0, 78.0]	62.0 [45.0, 80.0]	61.5 [50.0, 79.0]	62.0 [44.0, 81.0]
Sex							
Female	23 (50.0%)	932 (53.4%)	10 (66.7%)	358 (57.9%)	187 (55.7%)	24 (52.2%)	1534 (54.7%)
Male	23 (50.0%)	813 (46.6%)	5 (33.3%)	260 (42.1%)	149 (44.3%)	22 (47.8%)	1272 (45.3%)
Marriage status							
Living with a partner	31 (67.4%)	1233 (70.7%)	13 (86.7%)	410 (66.3%)	220 (65.5%)	24 (52.2%)	1931 (68.8%)
Not living with a partner	4 (8.7%)	128 (7.3%)	1 (6.7%)	53 (8.6%)	28 (8.3%)	6 (13.0%)	220 (7.8%)
Education							
College/university degree or above	31 (67.4%)	1030 (59.0%)	10 (66.7%)	373 (60.4%)	190 (56.5%)	28 (60.9%)	1662 (59.2%)
No college/university degree or above	14 (30.4%)	618 (35.4%)	4 (26.7%)	224 (36.2%)	130 (38.7%)	16 (34.8%)	1006 (35.9%)
Social Deprivation							
Above the Townsend deprivation index median	27 (58.7%)	999 (57.2%)	8 (53.3%)	360 (58.3%)	197 (58.6%)	27 (58.7%)	1618 (57.7%)
Below the Townsend deprivation index median	19 (41.3%)	743 (42.6%)	7 (46.7%)	257 (41.6%)	137 (40.8%)	19 (41.3%)	1182 (42.1%)
Lifestyle factors							
Smoking status							
Ever smoked	31 (67.4%)	1010 (57.9%)	9 (60.0%)	369 (59.7%)	181 (53.9%)	25 (54.3%)	1625 (57.9%)
Never smoked	15 (32.6%)	735 (42.1%)	6 (40.0%)	249 (40.3%)	155 (46.1%)	21 (45.7%)	1181 (42.1%)
Doing regular physical activity							
Yes	35 (76.1%)	1323 (75.8%)	12 (80.0%)	481 (77.8%)	252 (75.0%)	33 (71.7%)	2136 (76.1%)
No	11 (23.9%)	401 (23.0%)	3 (20.0%)	131 (21.2%)	81 (24.1%)	12 (26.1%)	639 (22.8%)
Healthy diet							
Yes	27 (58.7%)	887 (50.8%)	6 (40.0%)	315 (51.0%)	159 (47.3%)	28 (60.9%)	1422 (50.7%)
No	19 (41.3%)	852 (48.8%)	9 (60.0%)	300 (48.5%)	176 (52.4%)	18 (39.1%)	1374 (49.0%)
Alcohol consumption							
Excessive alcohol consumption	11 (23.9%)	373 (21.4%)	4 (26.7%)	130 (21.0%)	63 (18.8%)	7 (15.2%)	588 (21.0%)
Moderate alcohol consumption	30 (65.2%)	979 (56.1%)	9 (60.0%)	347 (56.1%)	185 (55.1%)	33 (71.7%)	1583 (56.4%)
Not current drinking	5 (10.9%)	387 (22.2%)	2 (13.3%)	141 (22.8%)	88 (26.2%)	6 (13.0%)	629 (22.4%)
Sleeping between 6 hours and 8 hours							
Yes	18 (39.1%)	752 (43.1%)	5 (33.3%)	266 (43.0%)	153 (45.5%)	22 (47.8%)	1216 (43.3%)
No	28 (60.9%)	984 (56.4%)	10 (66.7%)	349 (56.5%)	183 (54.5%)	24 (52.2%)	1578 (56.2%)
CVRFs factors							
Hypertension Status							
Yes	10 (21.7%)	359 (20.6%)	3 (20.0%)	112 (18.1%)	57 (17.0%)	10 (21.7%)	551 (19.6%)
No	36 (78.3%)	1386 (79.4%)	12 (80.0%)	506 (81.9%)	279 (83.0%)	36 (78.3%)	2255 (80.4%)
Diabetes							
Yes	1 (2.2%)	103 (5.9%)	1 (6.7%)	30 (4.9%)	20 (6.0%)	3 (6.5%)	158 (5.6%)
No	45 (97.8%)	1642 (94.1%)	14 (93.3%)	588 (95.1%)	316 (94.0%)	43 (93.5%)	2648 (94.4%)
BMI							
Mean(sd)	27.1 (5.08)	26.3 (4.20)	25.1 (3.93)	26.2 (4.14)	26.7 (4.61)	27.0 (4.95)	26.4 (4.26)
Median(range)	26.4 [18.6, 42.7]	25.7 [17.4, 48.0]	24.2 [20.2, 31.1]	25.9 [16.5, 48.4]	25.7 [18.1, 49.4]	26.1 [19.7, 38.4]	25.8 [16.5, 49.4]

*: Percentiles do not add up to 1 is due to missingness.

*:All variables are measured at the baseline except the age at imaging, hypertension status, and diabetes.

Supplementary Table S5 Demographic information, lifestyle factors and cardiovascular risk factors of the non-British UKB subjects used in the association study on fMRI connectivities. Percentiles may not add up to 1 due to missingness. All variables were measured at baseline, with the exception of age at imaging, diabetes status, and hypertension status.

FC Measures	APOE4	Hypertension	BMI	Smoking	Alcohol	Physical Activity	Sleep	Education	Age & APOE Interaction	Other (Sex) Interaction
Ji12-NFC	N: 6 (5)	N: 12 (12)	P: 33 (27)<br>N: 7 (5)	P: 9 (7)<br>N: 6 (5)	**Ex. Alcoh**<br>N: 4 (0)	P: 1 (1)<br>N: 1 (1)	P: 1 (1)<br>N: 1 (1)	P: 13 (11)<br>N: 4 (4)	**Age*Hypertension**<br>P: 1 (1)<br>**Age*Sex**<br>P: 3 (3); N: 1 (0)<br>**APOE4*Sex**<br>N: 1 (1)	**Sex*Smoking**<br>N: 2 (1)<br>**Sex*Diabetes**<br>P: 1 (1)<br>**Sex*BMI**<br>P: 6 (4); N: 2 (2)<br>**Sex*Edu**<br>N: 2 (1)
Ji12-NES	N: 7 (5)	N: 33 (33)	P: 6 (4)<br>N: 20 (20)	P: 1 (1)<br>N: 9 (9)	**Ex. Alcoh**<br>N: 5 (5)	P: 1 (1)	P: 1 (1)	P: 16 (16)<br>N: 1 (0)	**Age*BMI**<br>N: 1 (1)<br>**Age*Sex**<br>P: 18 (18)	**Sex*Smoking**<br>N: 2 (0)<br>**Sex*BMI**<br>P: 1 (0); N: 4 (2)<br>**Sex*Edu**<br>P: 1 (1)<br>**Sex*Mod.Alcoh**<br>P: 2 (2)<br>**Sex*Ex.Alcoh**<br>N: 2 (1)
Yeo7-NFC	N: 7 (5)	N: 2 (2)	P: 15 (14)<br>N: 1 (0)	P: 6 (5)<br>N: 3 (2)	**Mod. Alcoh**<br>P: 1 (0)<br>**Ex. Alcoh**<br>P: 1 (1)<br>N: 3 (0)	P: 2 (2)	P: 3 (3)	P: 8 (8)	**Age*Hypertension**<br>P: 1 (1)<br>**Age*Sex**<br>P: 3 (3); N: 2 (1)<br>**APOE4*Sex**<br>N: 1 (0)<br>**APOE4*Sex*Diabetes**<br>P: 1 (0)<br>**APOE4*Sex*BMI**<br>P: 1 (0)	**Sex*BMI**<br>P: 11 (11)<br>**Sex*Ex.Alcoh**<br>N: 1 (1)
Yeo7-NES	N: 4 (4)	N: 7 (7)	P: 10 (9)<br>N: 6 (6)	P: 1 (0)<br>N: 5 (4)	**Ex. Alcoh**<br>N: 4 (4)	P: 2 (2)	P: 2 (2)	P: 6 (6)	**Age*BMI**<br>N: 1 (1)<br>**Age*Sex**<br>P: 7 (7); N: 1 (0)<br>**Age*APOE4*Edu**<br>N: 1 (1)<br>**APOE4*Sex*Diabetes**<br>P: 1 (0)	**Sex*BMI**<br>P: 2 (2)<br>**Sex*Ex.Alcoh**<br>N: 1 (1)<br>**Sex*SoDep**<br>N : 1 (0)
Yeo17-NFC	N: 15 (8)	P: 2 (2)<br>N: 8 (7)	P: 66 (62)<br>N: 2 (1)	P: 9 (7)<br>N: 6 (6)	**Ex. Alcoh**<br>P: 3 (2)<br>N: 9 (3)	P: 4 (4)	P: 2 (1)	P: 27 (25)<br>N: 3 (2)	**Age*BMI**<br>N: 1 (1)<br>**Age*Sex**<br>P: 8 (6); N: 5 (3)<br>**Age*APOE4*Edu**<br>N: 1 (1)<br>**Age*APOE4*Sleep**<br>P: 1 (1)<br>**APOE4*Sex**<br>N: 1 (0)<br>**APOE4*Sex*BMI**<br>P: 1 (0)<br>**APOE4*Sex*Diabetes**<br>P: 2 (0)	**Sex*BMI**<br>P: 21 (20);<br>N: 1 (1)
Yeo17-NES	N: 3 (3)	N: 21 (21)	P: 32 (28)<br>N: 18 (17)	P: 1 (1)<br>N: 7 (7)	**Ex. Alcoh**<br>N: 3 (3)	P: 3 (3)	P: 1 (1)	P: 14 (14)<br>N: 3 (2)	**Age*BMI**<br>N: 1 (1)<br>**Age*Diet**<br>N: 1 (0)<br>**Age*Sex**<br>P: 11 (10); N: 2 (0)<br>**Age*APOE4*Edu**<br>N: 1 (1)<br>**APOE4*Sex*Diabetes**<br>P: 2 (0)	**Sex*BMI**<br>P: 6 (5); N: 1 (1)<br>**Sex*Edu**<br>P: 1 (1)<br>**Sex*Mod.Alcoh**<br>P: 1 (0)<br>**Sex*Ex.Alcoh**<br>N: 3 (1)

Supplementary Table S6. Summary of the numbers of associations between brain functional connectivity (FC) measures and *APOE4*, cardiovascular, lifestyle, and socioeconomic factors, as well as their interactions with age, sex, and the *APOE4* gene, as identified in the main findings by the British population across three network atlases (Ji-12, Yeo-7, and Yeo-17). The numbers of validated findings from non-British populations are listed in parentheses. (-) indicates missing p-value/effect size due to insufficient sample size. Entries that are 100% validated by the non-British population are highlighted in yellow. The explanations of FC measures are provided in **Supplementary Table S3**. P: Positive (association); N: negative; Ex.Alcoh: excessive alcohol consumption; Mod.Alcoh: moderate alcohol consumption; SoDep: the index of social deprivation; Edu: education.

Figures

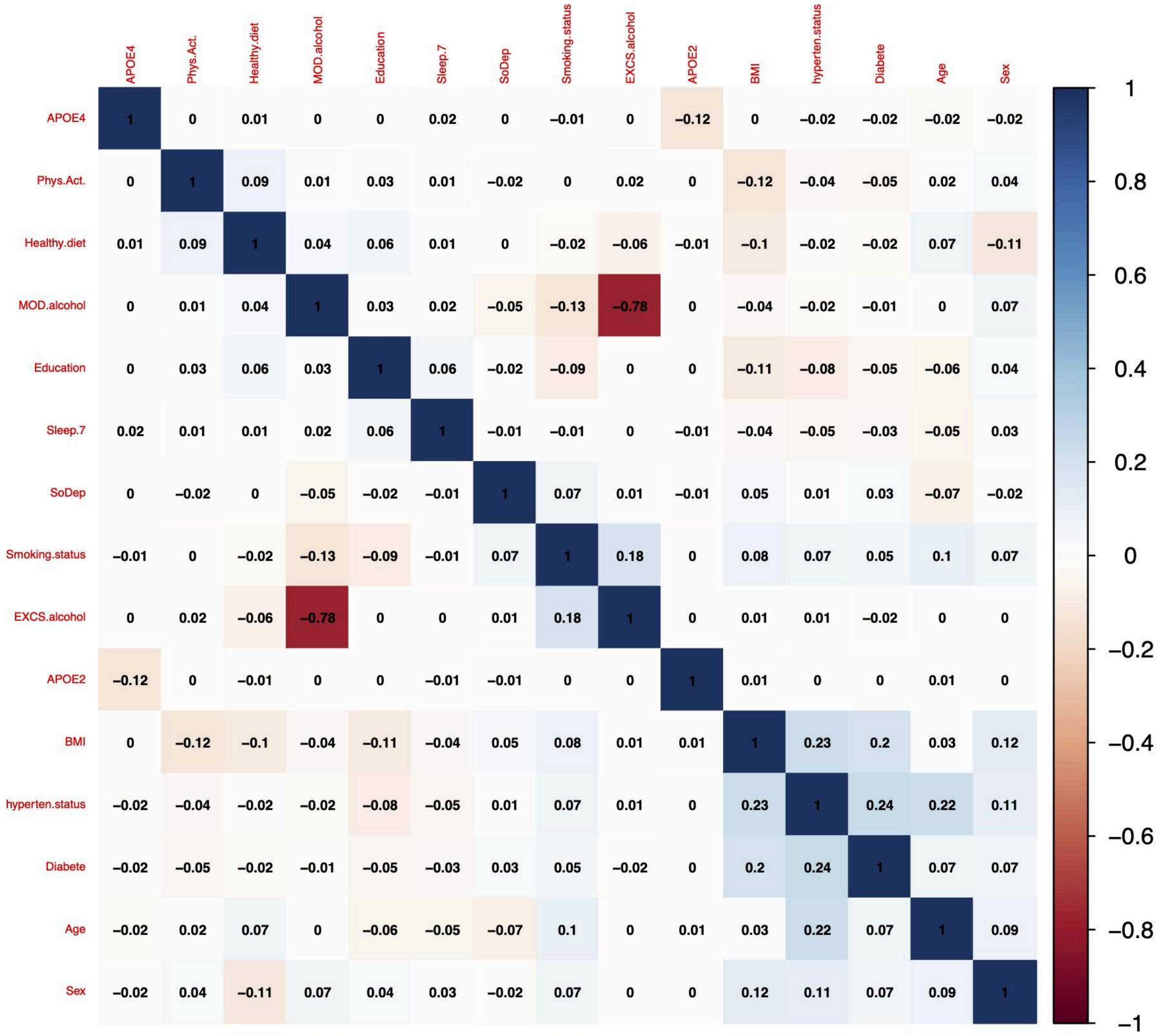

Supplementary Figure S1 Correlation plot of APOE, socioeconomic status, cardiovascular risk factors and lifestyle variables. SoDep, Social deprivation Index; NotSmoking, not-current-smoker; Phys.Act, physical activity; Healthy.diet, healthy diet; Sleep.7, healthy sleep; MOD.alcohol, moderate alcohol; EXCS.alcohol, excessive alcohol;

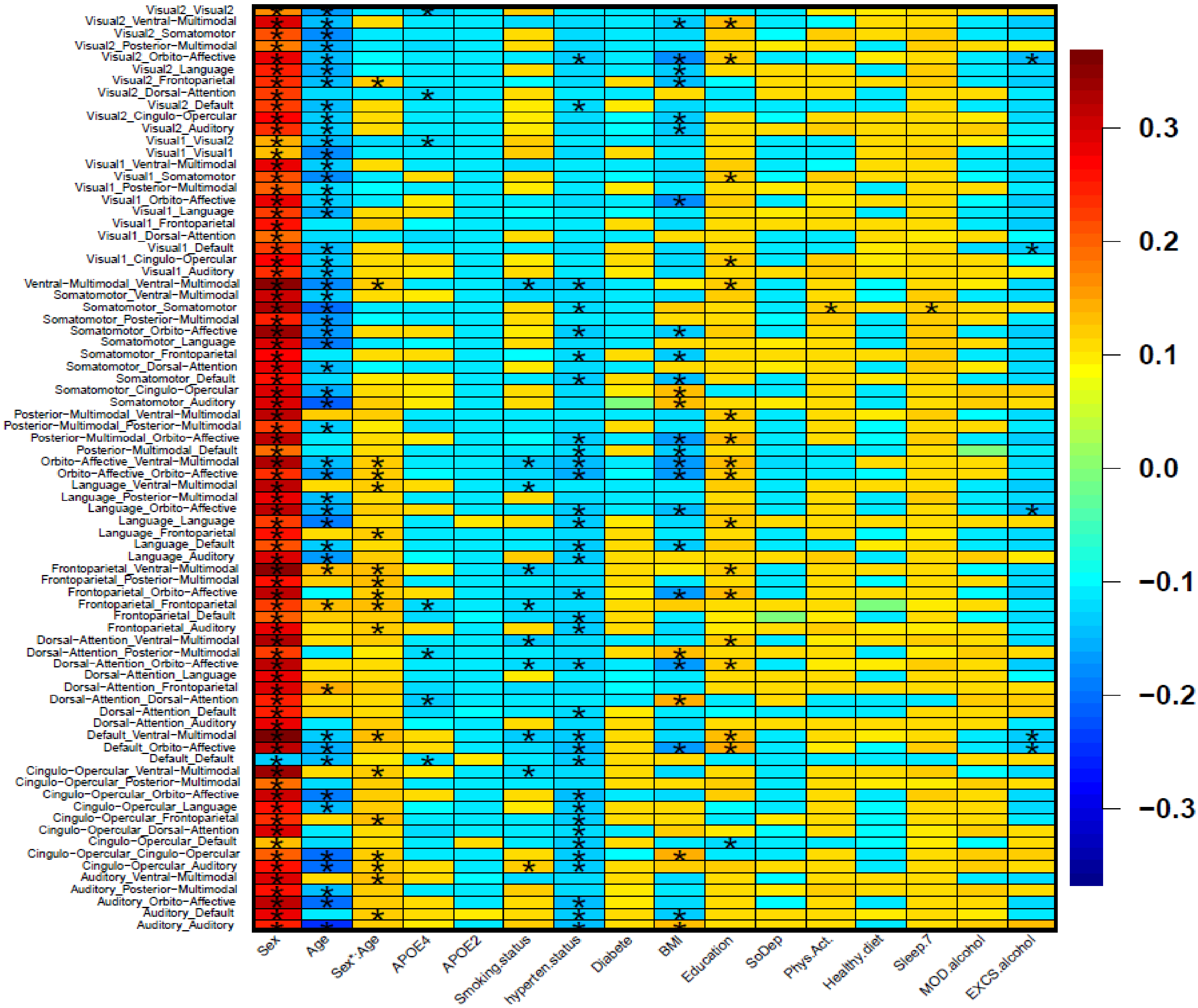

Supplementary Figure S2. Heatmap of all the identified main effects of demographic, lifestyle, social economic status (SES) factors, and cardiovascular risk factors (CVRF) on the network edge strength (NES) measures, based on the Ji-12 network atlas. Results that passed the Bonferroni-corrected significance level of *6.4E-4* will be shown by (*). The effect sizes (standardized coefficients) for each association are illustrated using a color gradient from blue to red.

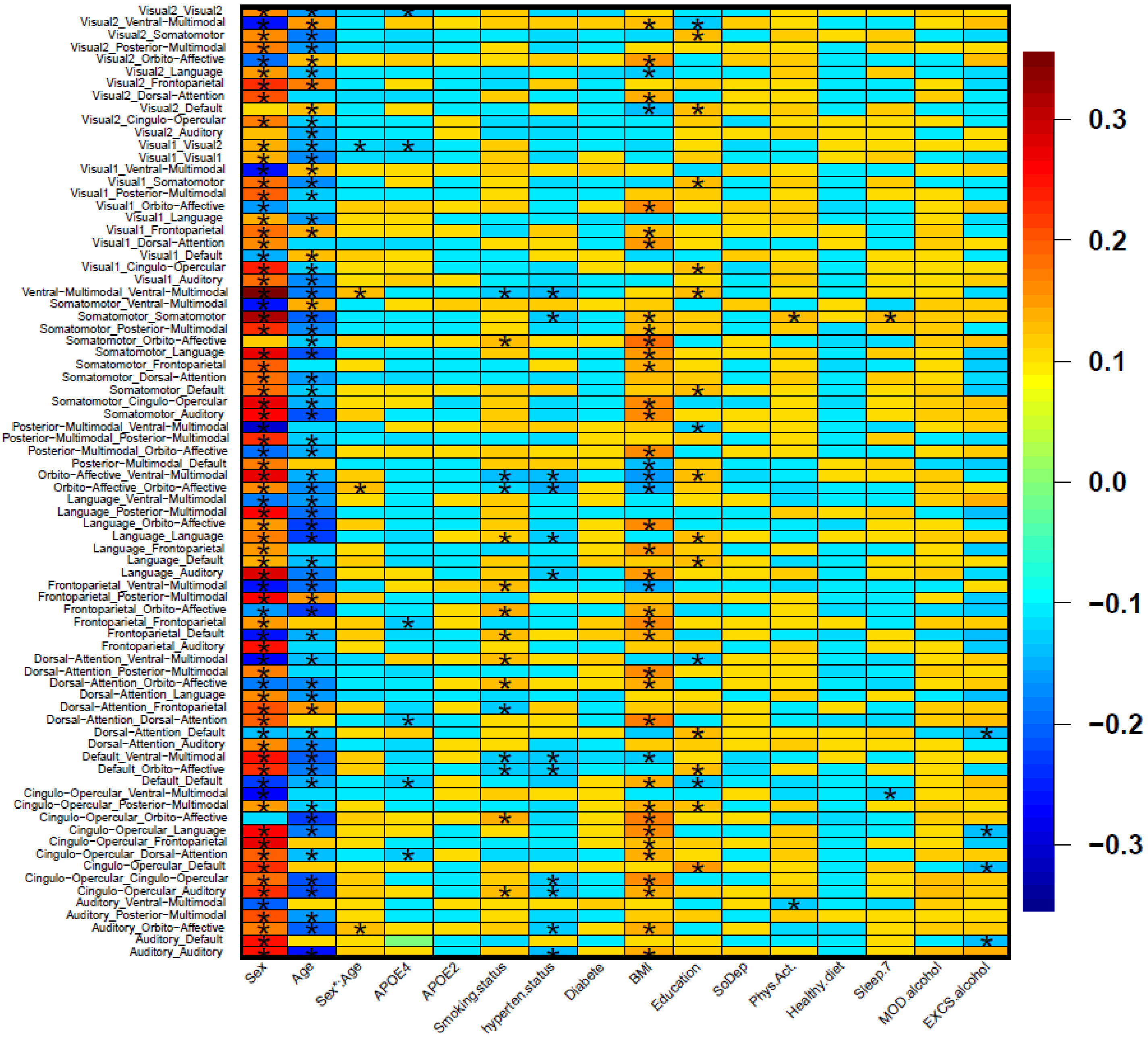

Supplementary Figure S3. Heatmap of all the identified main effects of demographic, lifestyle, social economic status (SES) factors, and cardiovascular risk factors (CVRF) on the network functional connectivity (NFC) measures, based on the Ji-12 network atlas. Results that passed the Bonferroni-corrected significance level of *6.4E-4* will be shown by (*). The effect sizes (standardized coefficients) for each association are illustrated using a color gradient from blue to red.

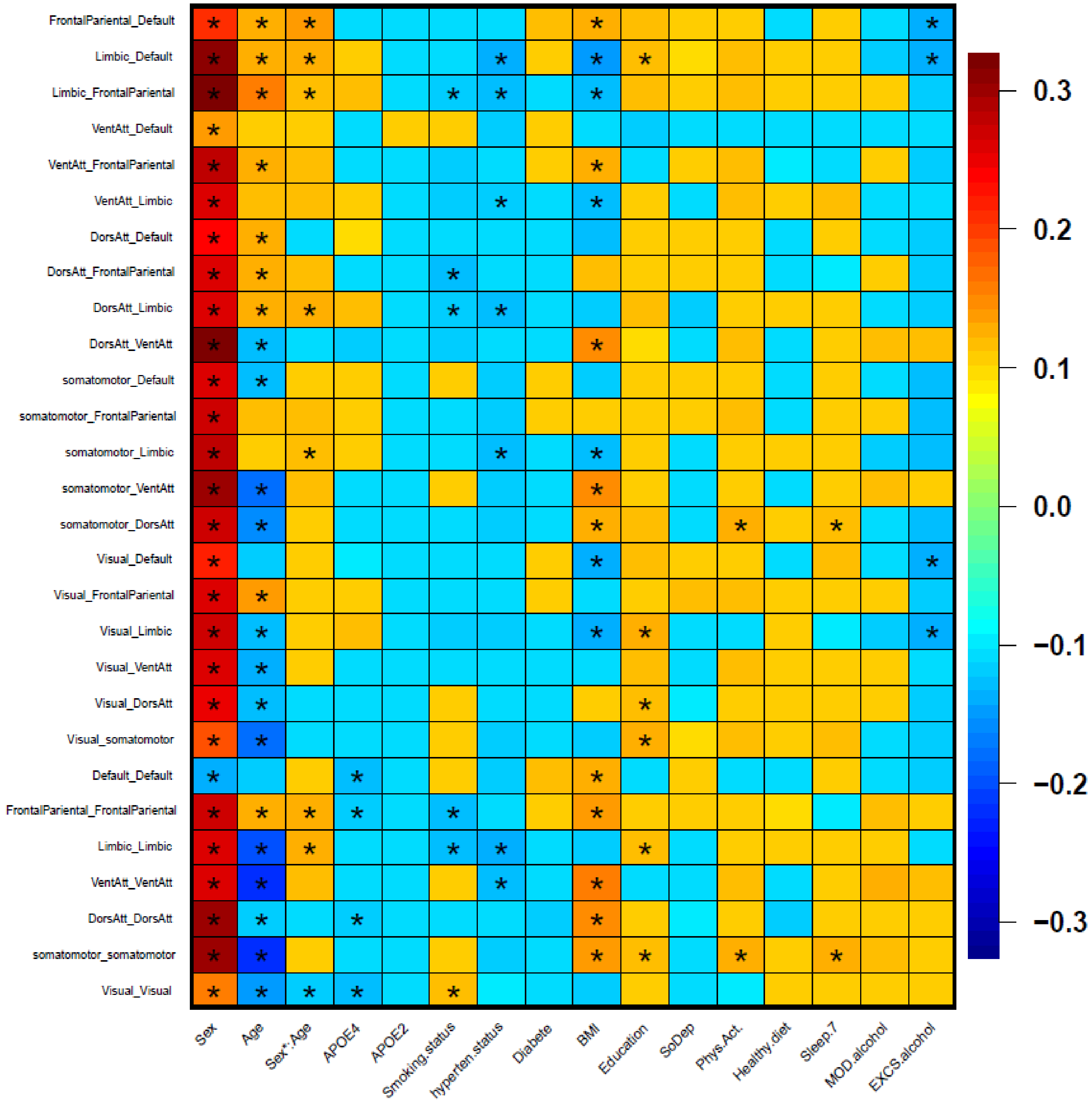

Supplementary Figure S4. Heatmap of all the identified main effects of demographic, lifestyle, social economic status (SES) factors, and cardiovascular risk factors (CVRF) on the network edge strength (NES) measures, based on the Yeo-7 network atlas. Results that passed the Bonferroni-corrected significance level of *1.8E-3* will be shown by (*). The effect sizes (standardized coefficients) for each association are illustrated using a color gradient from blue to red.

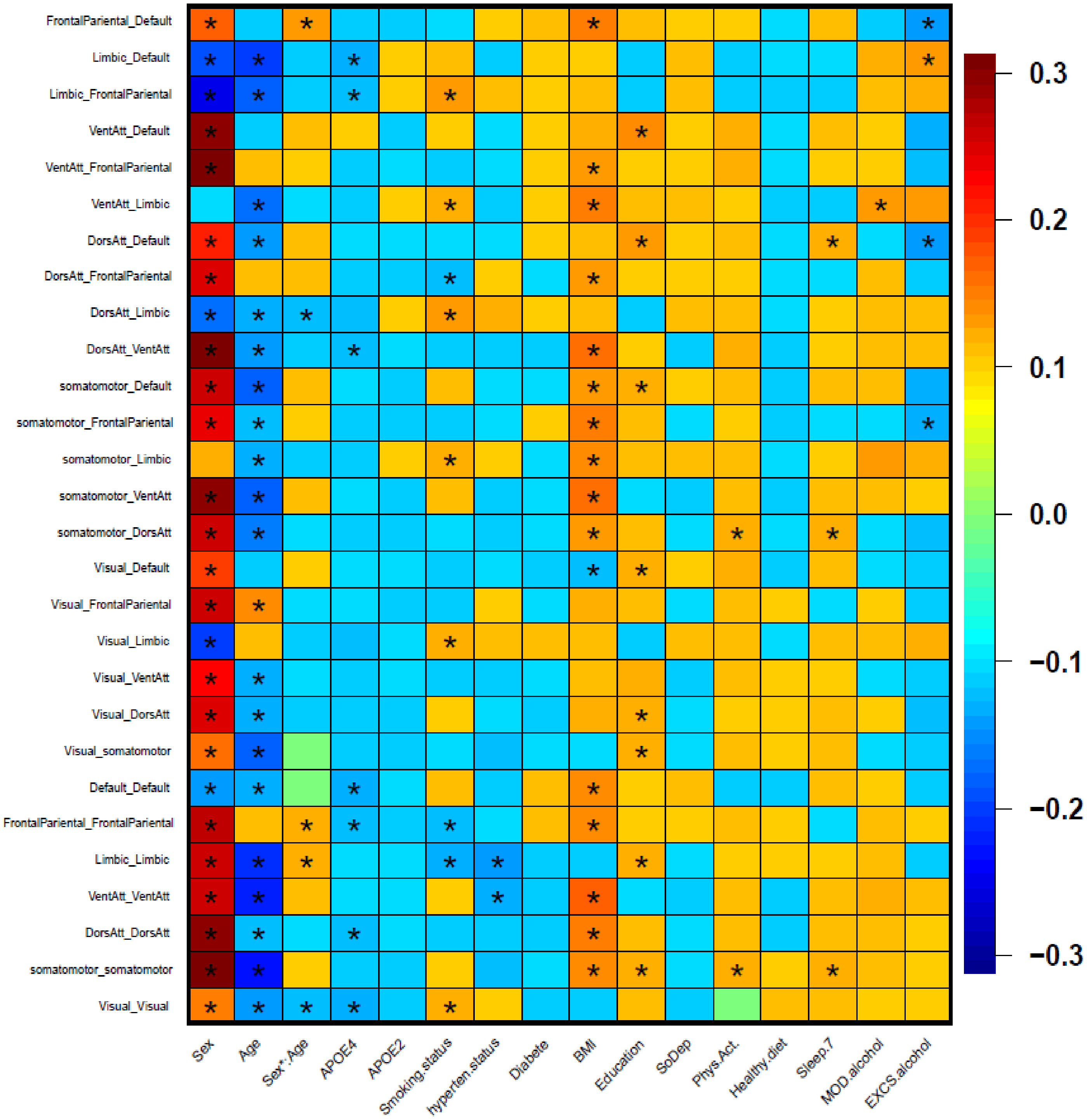

Supplementary Figure S5. Heatmap of all the identified main effects of demographic, lifestyle, social economic status (SES) factors, and cardiovascular risk factors (CVRF) on the network functional connectivity (NFC) measures, based on the Yeo-7 network atlas. Results that passed the Bonferroni-corrected significance level of *1.8E-3* will be shown by (*). The effect sizes (standardized coefficients) for each association are illustrated using a color gradient from blue to red.

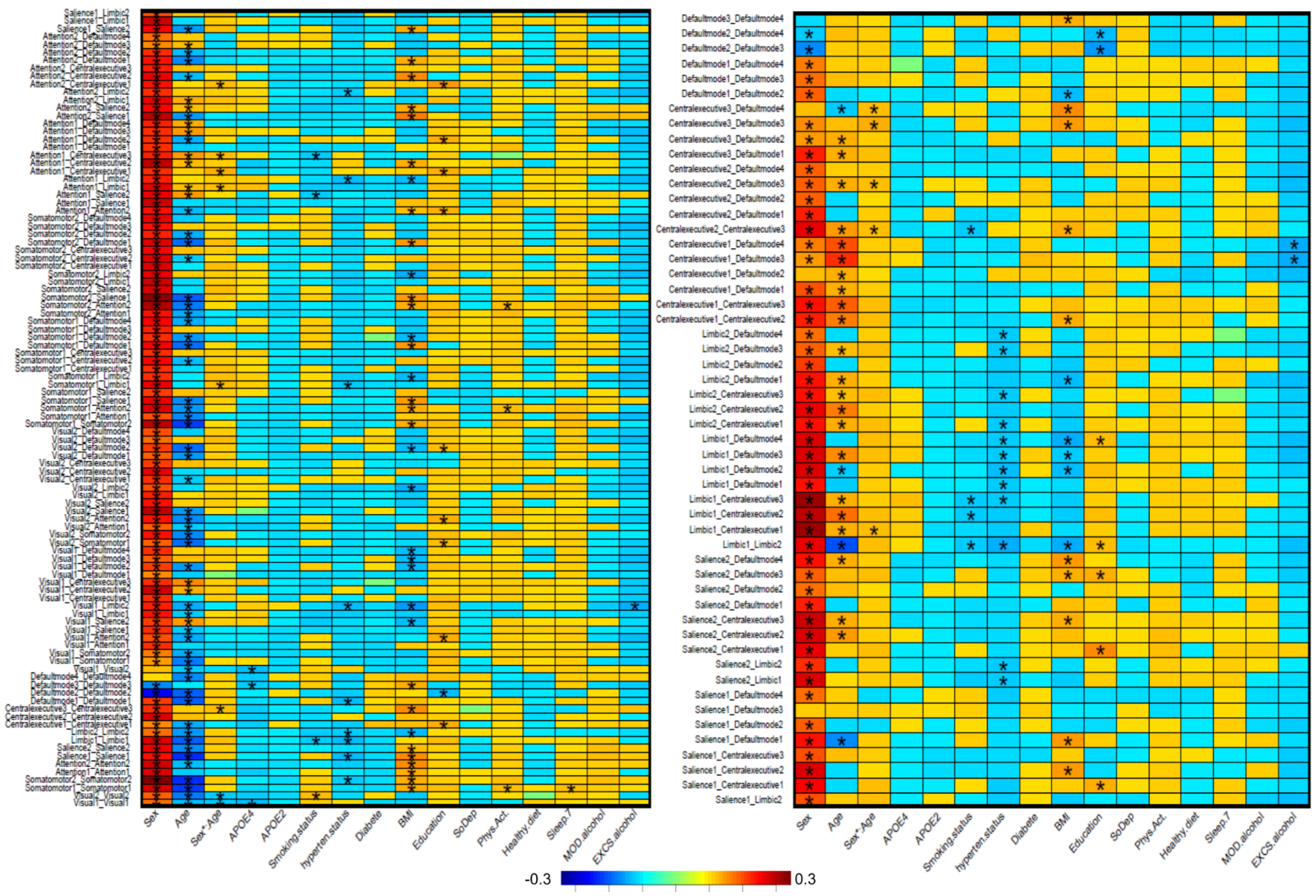

Supplementary Figure S6. Heatmap of all the identified main effects of demographic, lifestyle, social economic status (SES) factors, and cardiovascular risk factors (CVRF) on the network edge strength (NES) measures, based on the Yeo-17 network atlas. Results that passed the Bonferroni-corrected significance level of *3.3E-4* will be shown by (*). The effect sizes (standardized coefficients) for each association are illustrated using a color gradient from blue to red.

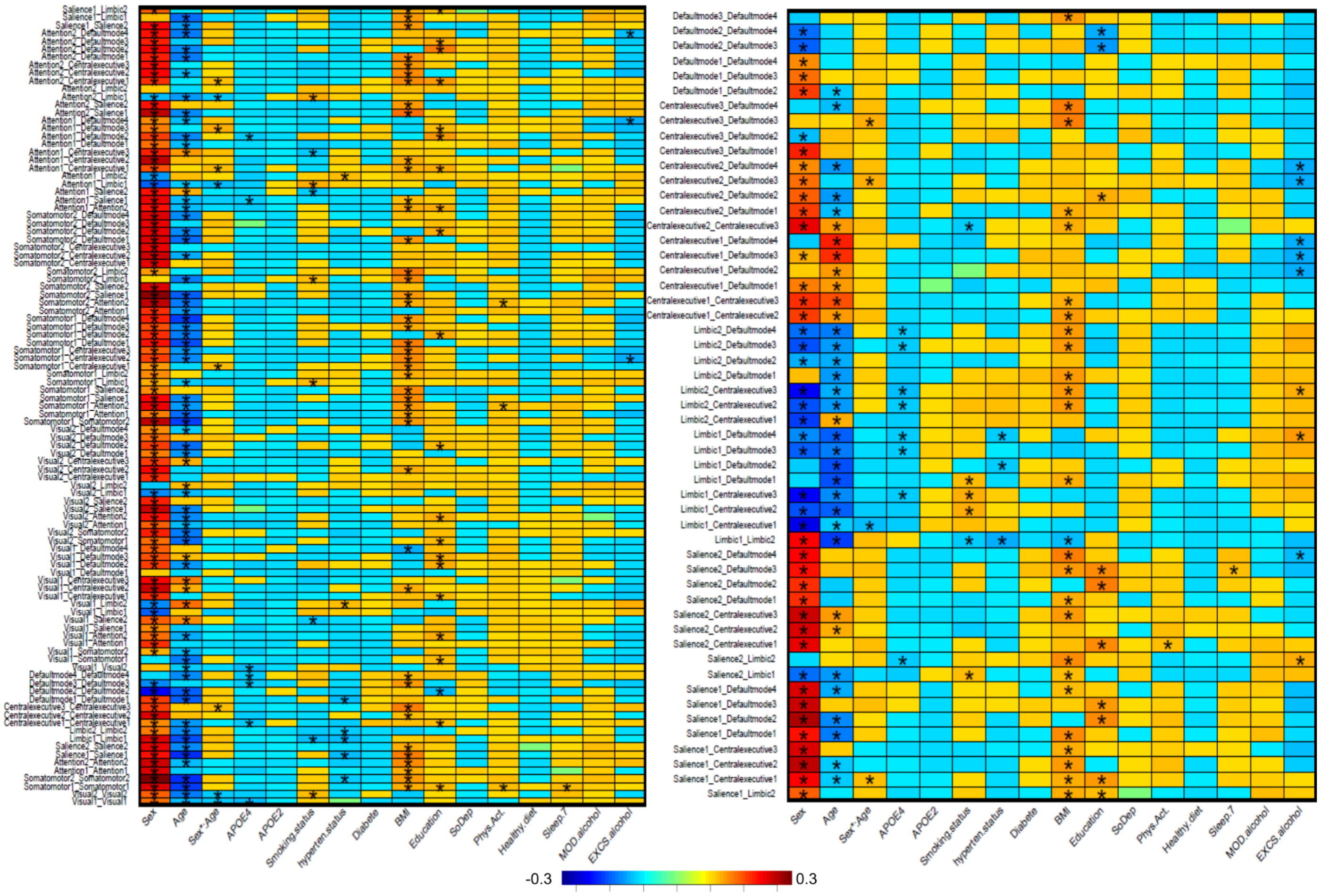

Supplementary Figure S7. Heatmap of all the identified main effects of demographic, lifestyle, social economic status (SES) factors, and cardiovascular risk factors (CVRF) on the network functional connectivity (NFC) measures, based on the Yeo-17 network atlas. Results that passed the Bonferroni-corrected significance level of *3.3E-4* will be shown by (*). The effect sizes (standardized coefficients) for each association are illustrated using a color gradient from blue to red.

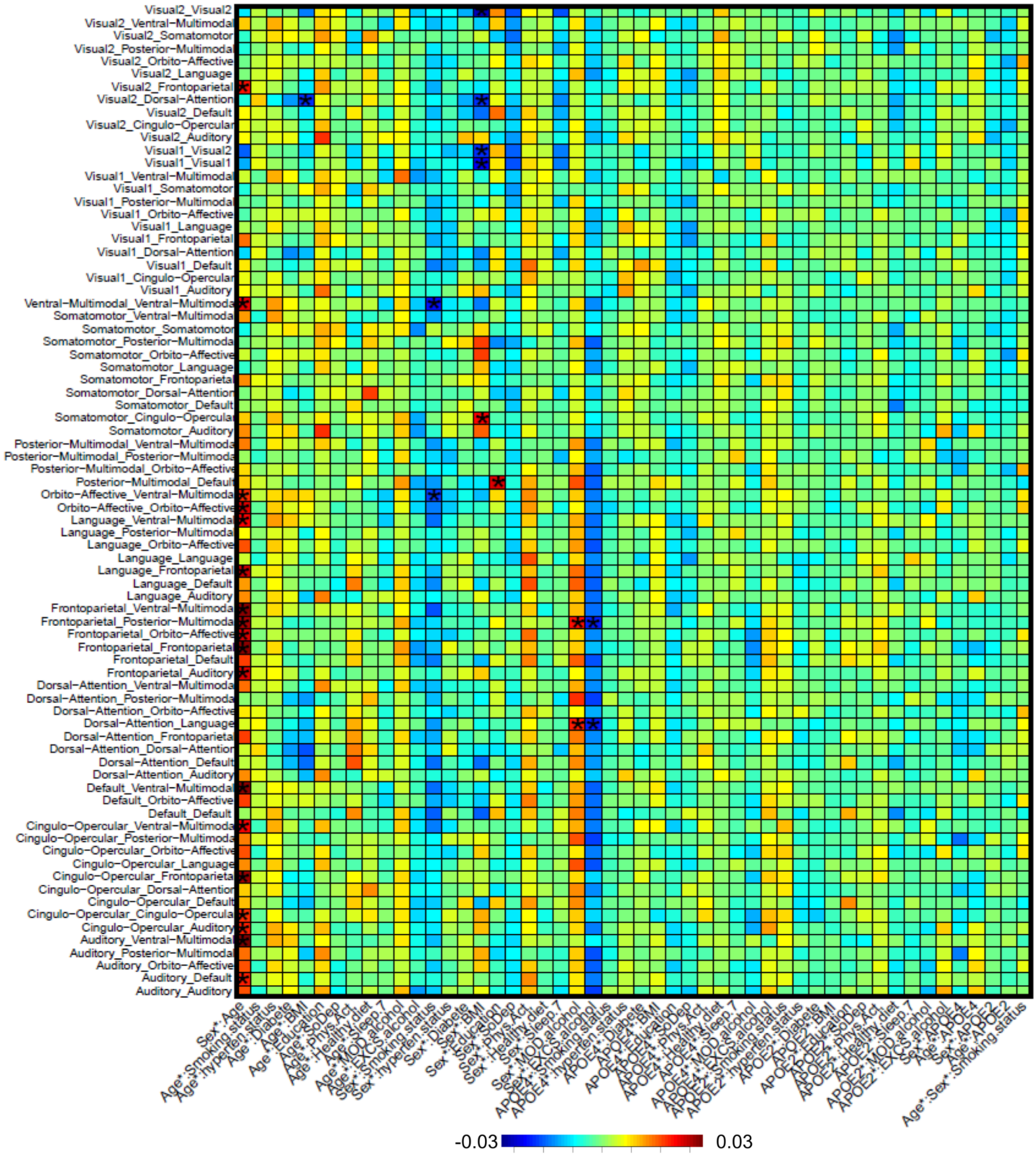

Supplementary Figure S8. Heatmap of all the identified two-way interaction effects of demographic, lifestyle, social economic status (SES) factors, and cardiovascular risk factors (CVRF) on the network edge strength (NES) measures, based on the Ji-12 network atlas. Results that passed the Bonferroni-corrected significance level of *6.4E-4* will be shown by (*). The effect sizes (standardized coefficients) for each association are illustrated using a color gradient from blue to red.

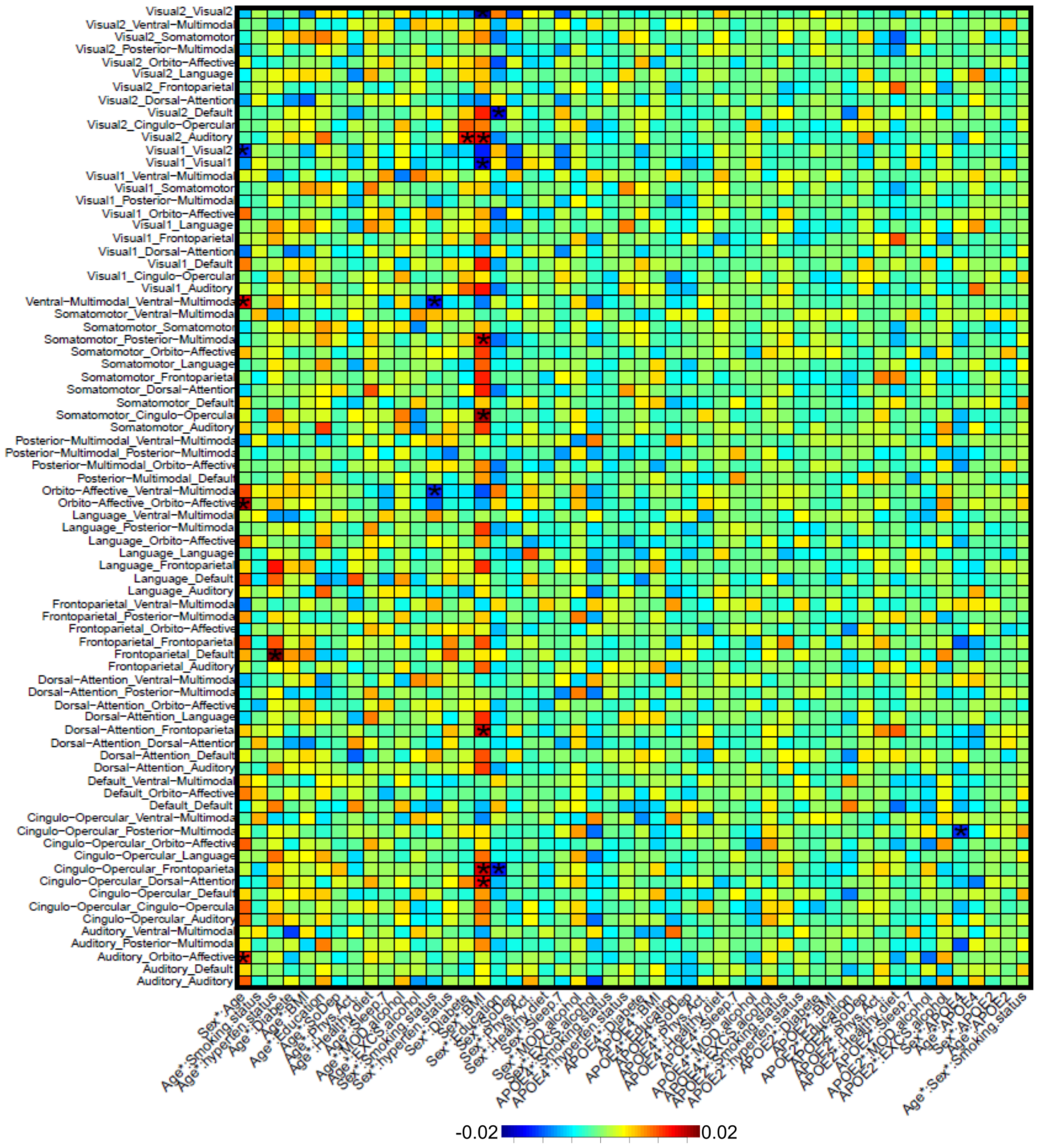

Supplementary Figure S9. Heatmap of all the identified two-way interaction effects of demographic, lifestyle, social economic status (SES) factors, and cardiovascular risk factors (CVRF) on the network functional connectivity (NFC) measures, based on the Ji-12 network atlas. Results that passed the Bonferroni-corrected significance level of *6.4E-4* will be shown by (*). The effect sizes (standardized coefficients) for each association are illustrated using a color gradient from blue to red.

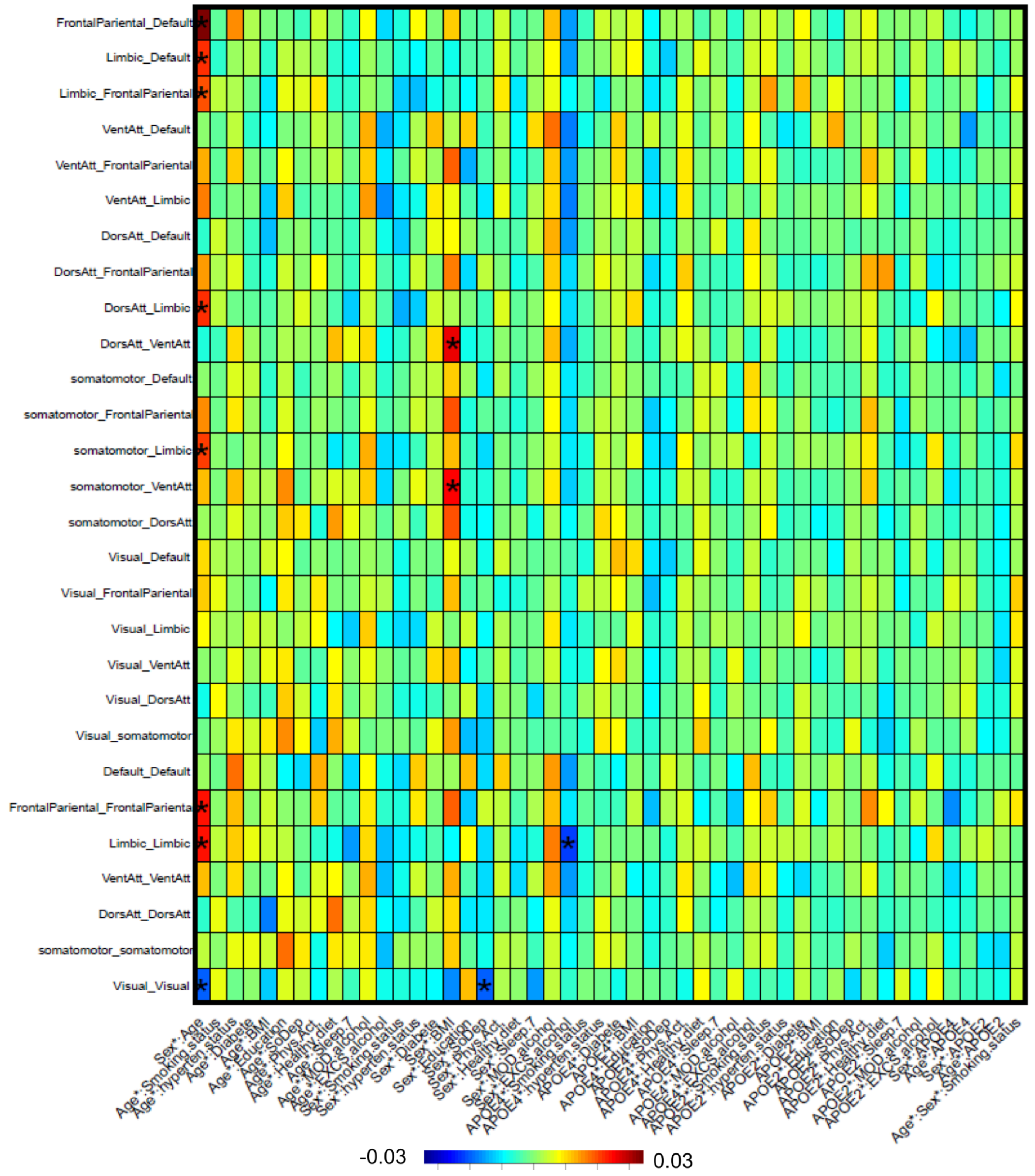

Supplementary Figure S10. Heatmap of all the identified two-way interaction effects of demographic, lifestyle, social economic status (SES) factors, and cardiovascular risk factors (CVRF) on the network edge strength (NES) measures, based on the Yeo-7 network atlas. Results that passed the Bonferroni-corrected significance level of *1.8E-3* will be shown by (*). The effect sizes (standardized coefficients) for each association are illustrated using a color gradient from blue to red.

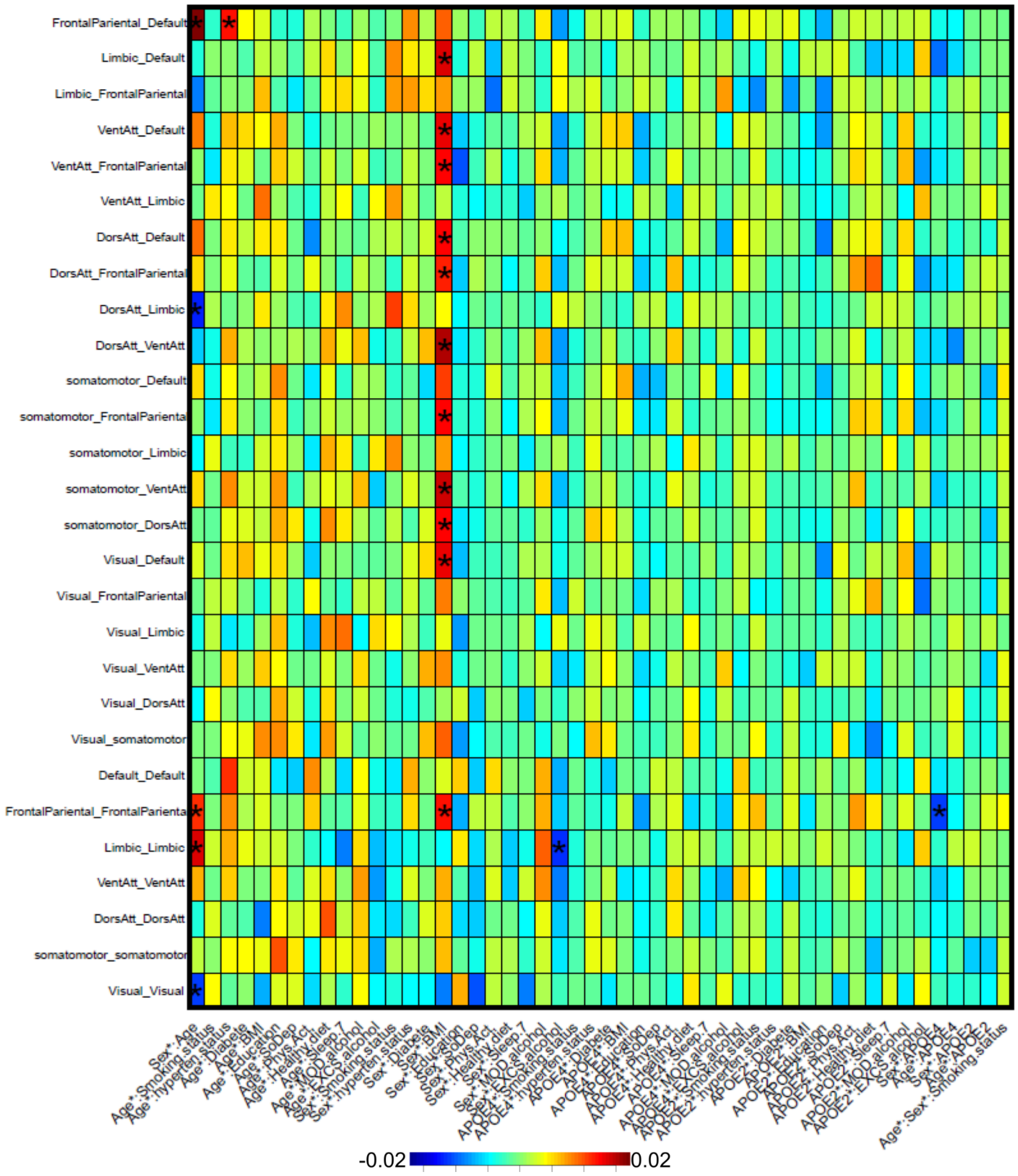

Supplementary Figure S11. Heatmap of all the identified two-way interaction effects of demographic, lifestyle, social economic status (SES) factors, and cardiovascular risk factors (CVRF) on the network functional connectivity (NFC) measures, based on the Yeo-7 network atlas. Results that passed the Bonferroni-corrected significance level of *1.8E-3* will be shown by (*). The effect sizes (standardized coefficients) for each association are illustrated using a color gradient from blue to red.

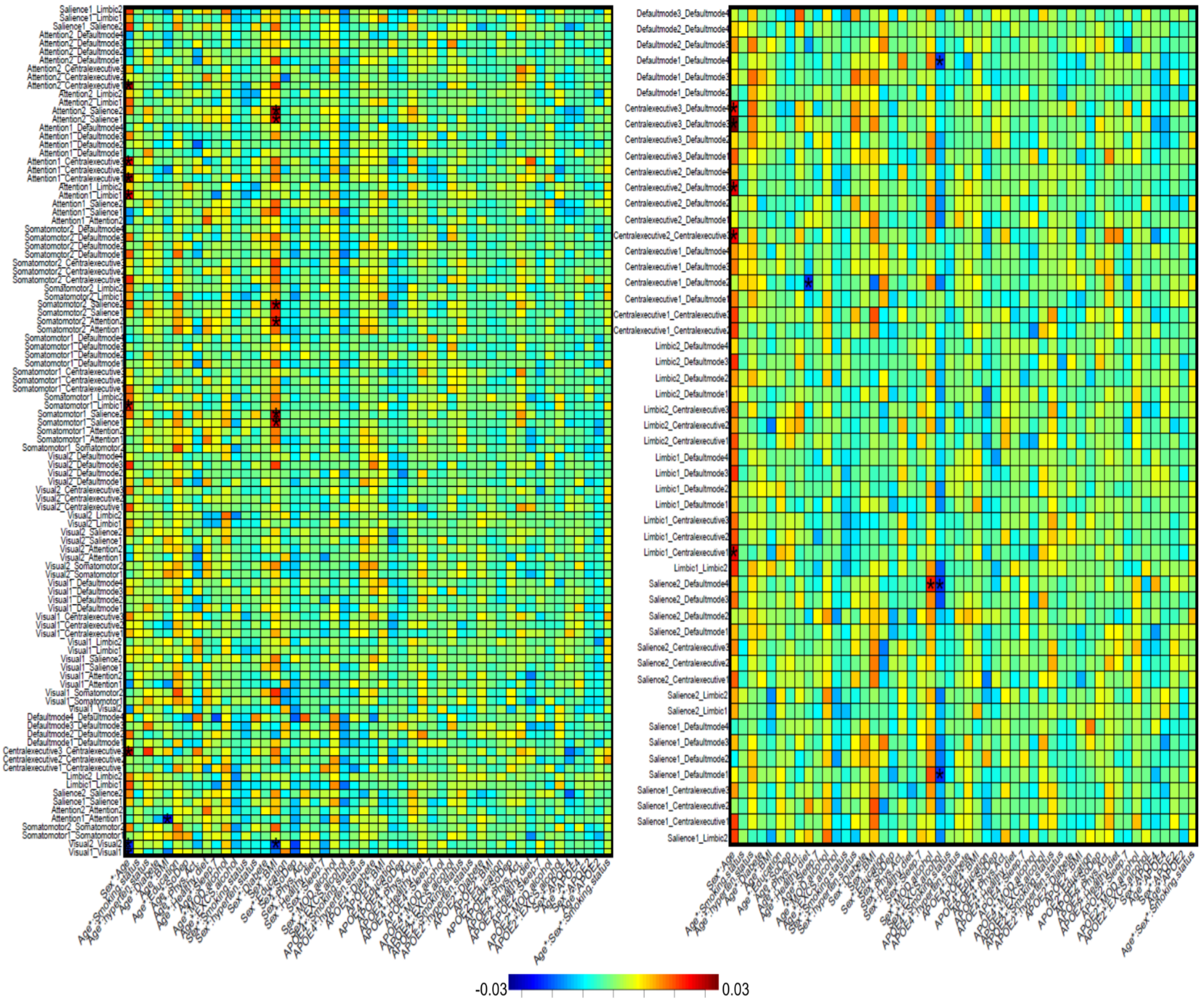

Supplementary Figure S12. Heatmap of all the identified two-way interaction effects of demographic, lifestyle, social economic status (SES) factors, and cardiovascular risk factors (CVRF) on the network edge strength (NES) measures, based on the Yeo-17 network atlas. Results that passed the Bonferroni-corrected significance level of *3.3E-4* will be shown by (*). The effect sizes (standardized coefficients) for each association are illustrated using a color gradient from blue to red.

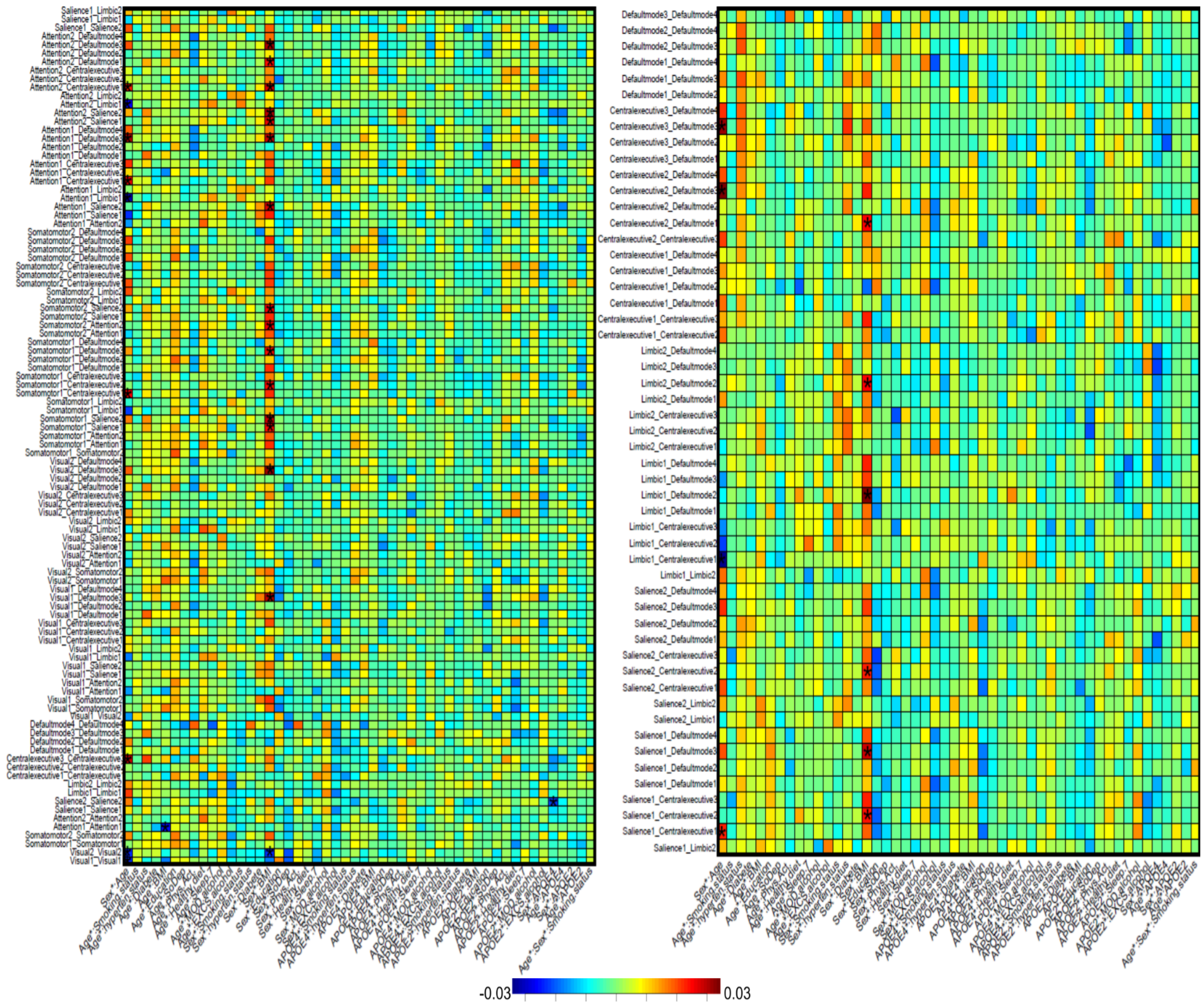

Supplementary Figure S13. Heatmap of all the identified two-way interaction effects of demographic, lifestyle, social economic status (SES) factors, and cardiovascular risk factors (CVRF) on the network functional connectivity (NFC) measures, based on the Yeo-17 network atlas. Results that passed the Bonferroni-corrected significance level of *3.3E-4* will be shown by (*). The effect sizes (standardized coefficients) for each association are illustrated using a color gradient from blue to red.

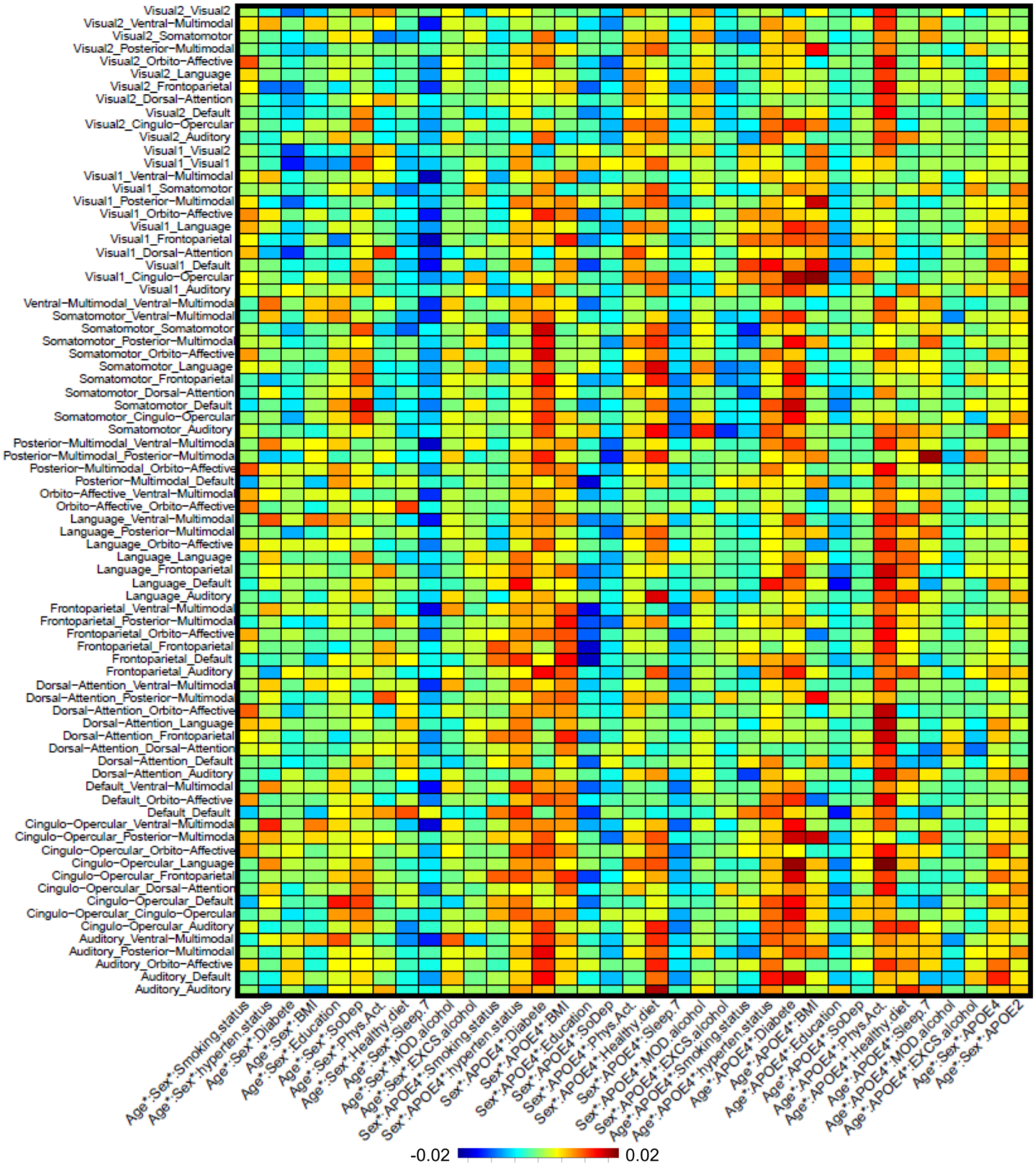

Supplementary Figure S14. Heatmap of all the identified three-way interaction effects of demographic, lifestyle, social economic status (SES) factors, and cardiovascular risk factors (CVRF) on the network edge strength (NES) measures, based on the Ji-12 network atlas. Results that passed the Bonferroni-corrected significance level of *6.4E-4* will be shown by (*). The effect sizes (standardized coefficients) for each association are illustrated using a color gradient from blue to red.

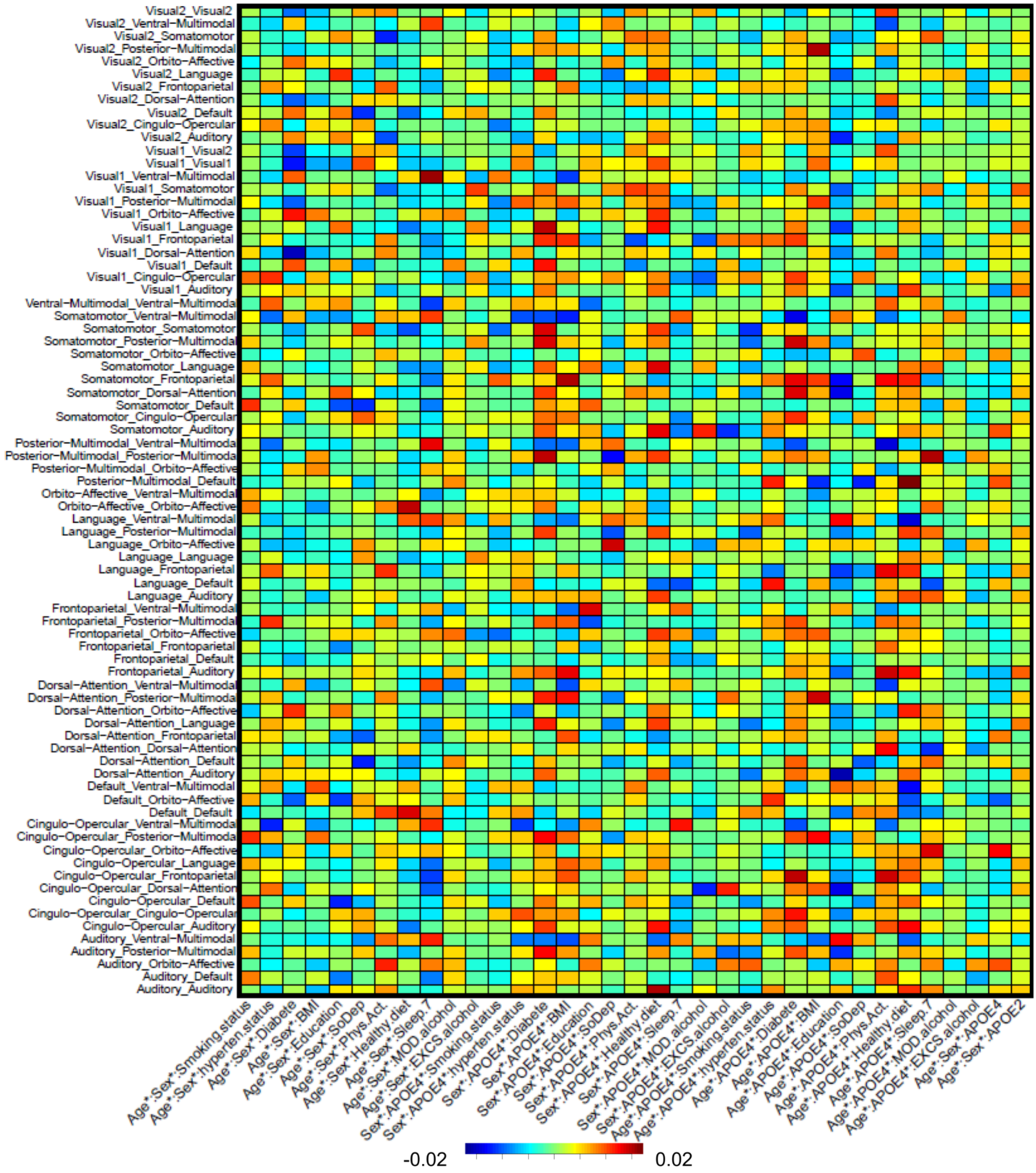

Supplementary Figure S15. Heatmap of all the identified three-way interaction effects of demographic, lifestyle, social economic status (SES) factors, and cardiovascular risk factors (CVRF) on the network functional connectivity (NFC) measures, based on the Ji-12 network atlas. Results that passed the Bonferroni-corrected significance level of *6.4E-4* will be shown by (*). The effect sizes (standardized coefficients) for each association are illustrated using a color gradient from blue to red.

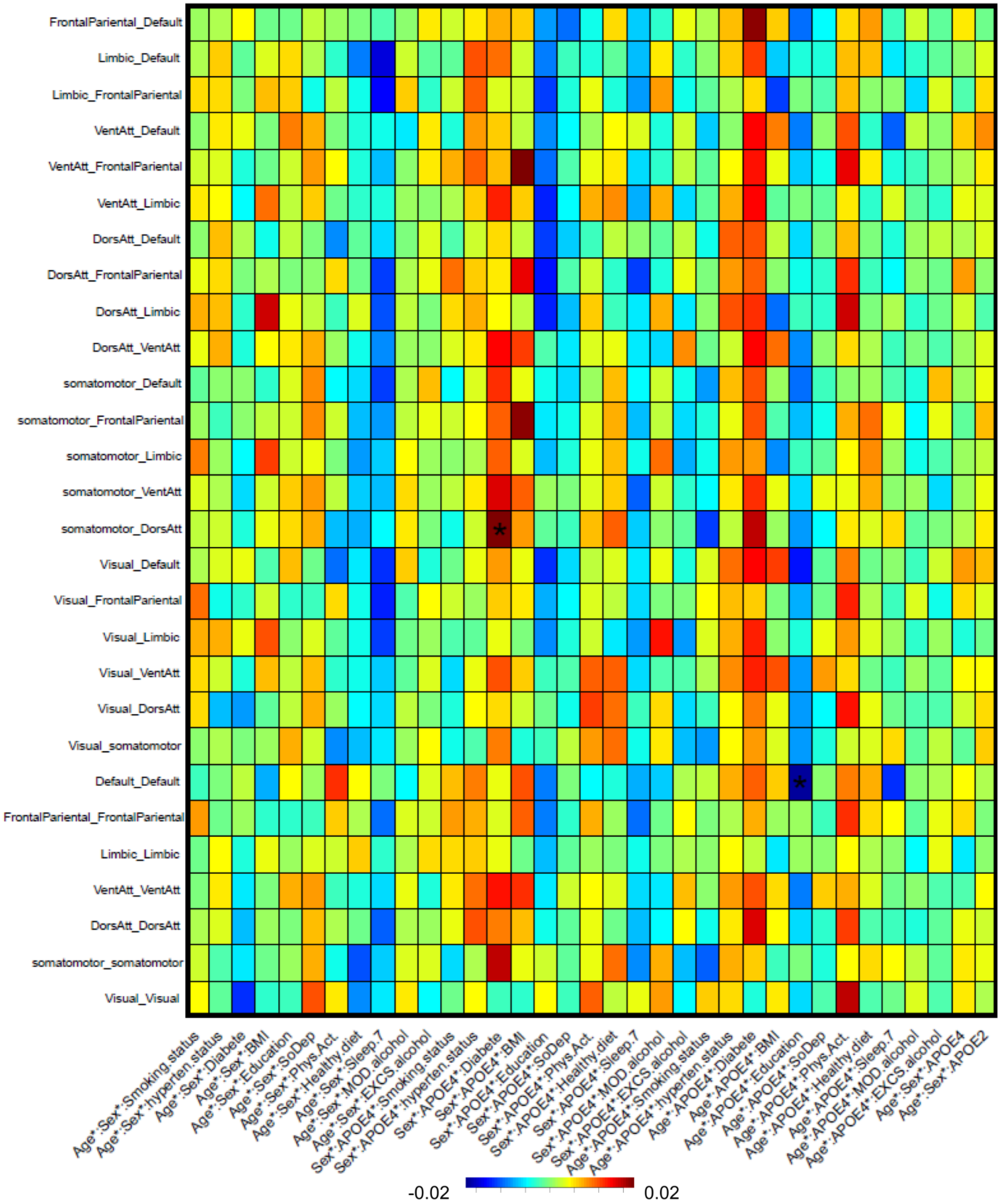

Supplementary Figure S16. Heatmap of all the identified three-way interaction effects of demographic, lifestyle, social economic status (SES) factors, and cardiovascular risk factors (CVRF) on the network edge strength (NES) measures, based on the Yeo-7 network atlas. Results that passed the Bonferroni-corrected significance level of *1.8E-3* will be shown by (*). The effect sizes (standardized coefficients) for each association are illustrated using a color gradient from blue to red.

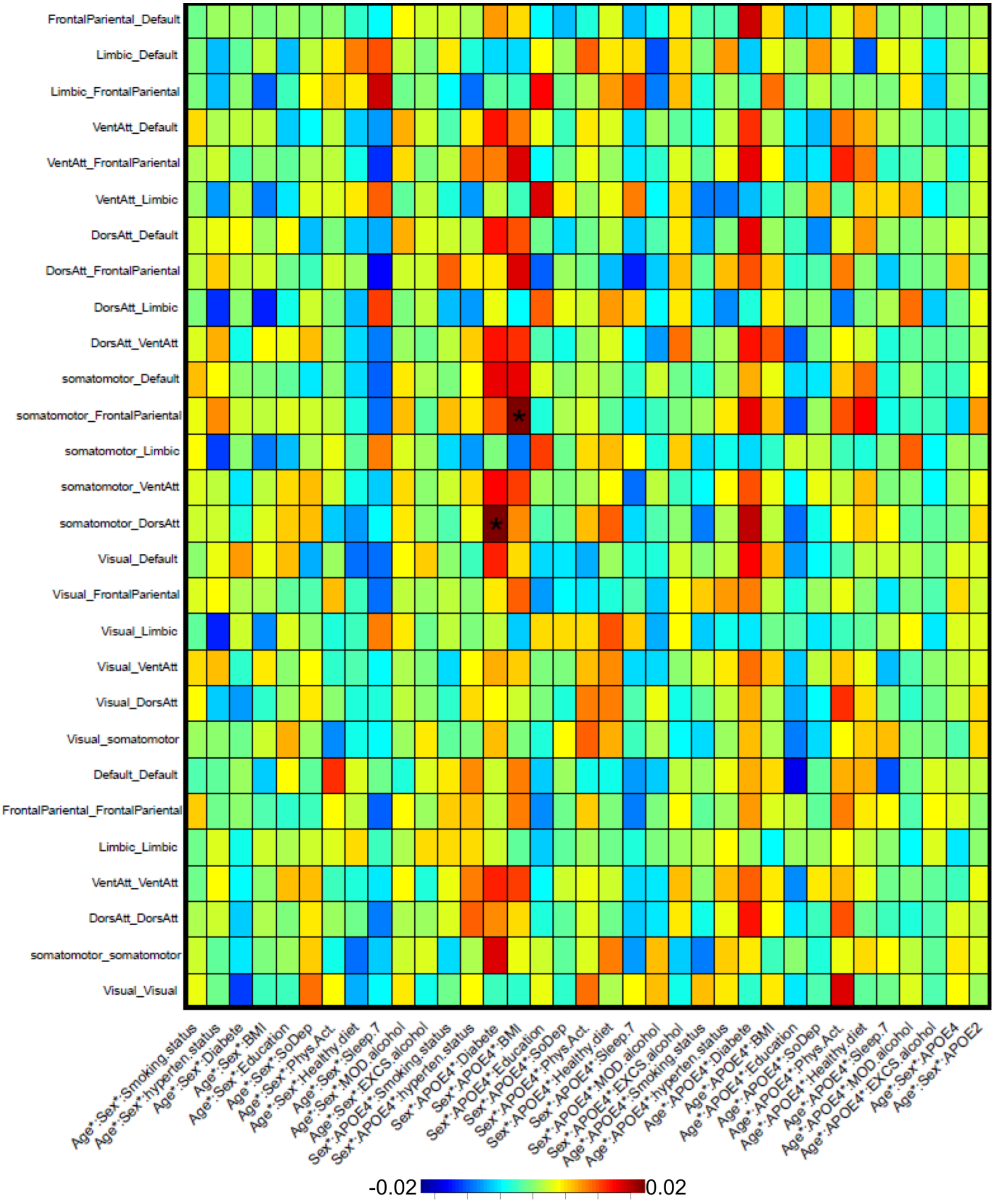

Supplementary Figure S17. Heatmap of all the identified three-way interaction effects of demographic, lifestyle, social economic status (SES) factors, and cardiovascular risk factors (CVRF) on the network functional connectivity (NFC) measures, based on the Yeo-7 network atlas. Results that passed the Bonferroni-corrected significance level of *1.8E-3* will be shown by (*). The effect sizes (standardized coefficients) for each association are illustrated using a color gradient from blue to red.

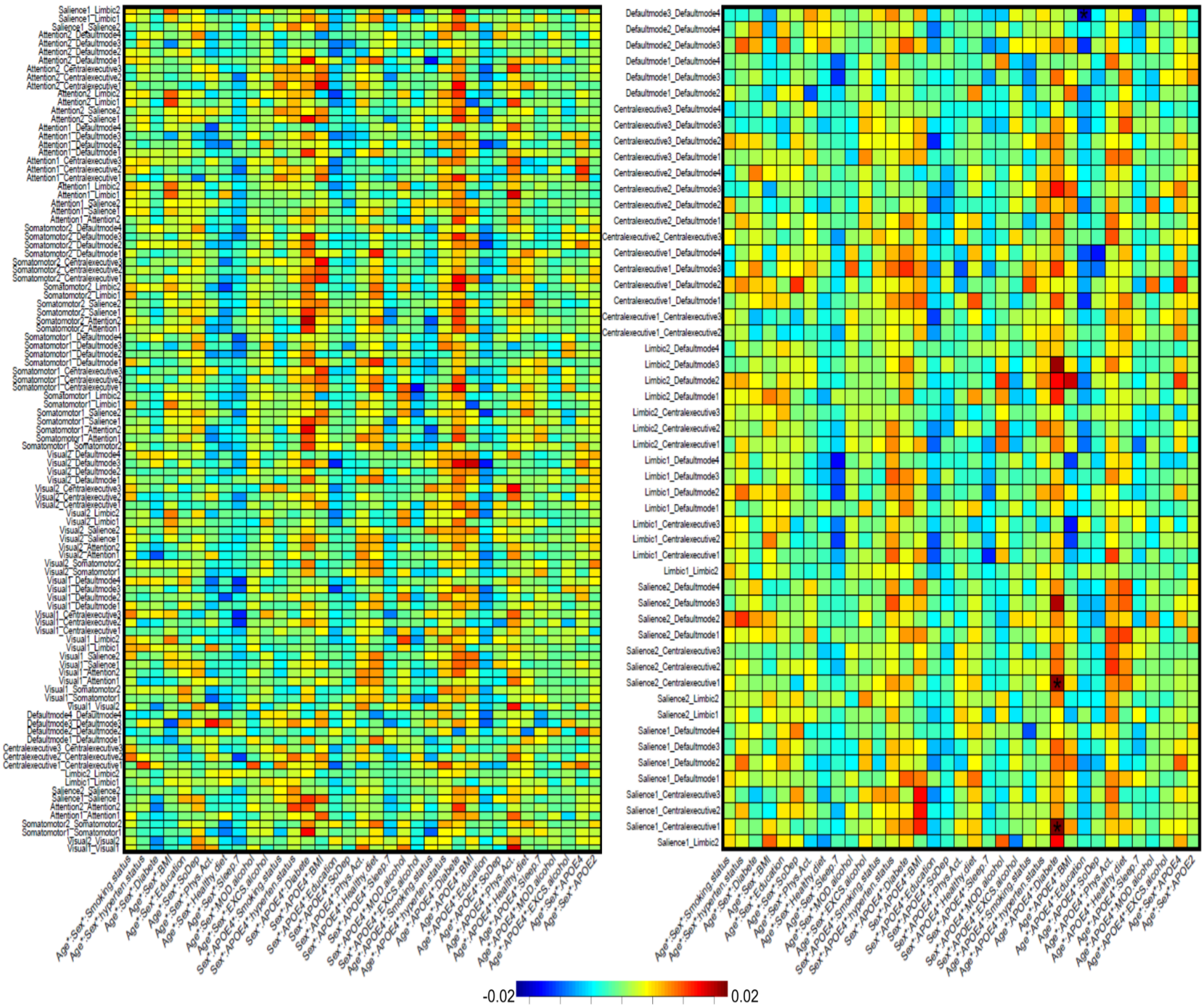

Supplementary Figure S18. Heatmap of all the identified three-way interaction effects of demographic, lifestyle, social economic status (SES) factors, and cardiovascular risk factors (CVRF) on the network edge strength (NES) measures, based on the Yeo-17 network atlas. Results that passed the Bonferroni-corrected significance level of *3.3E-4* will be shown by (*). The effect sizes (standardized coefficients) for each association are illustrated using a color gradient from blue to red.

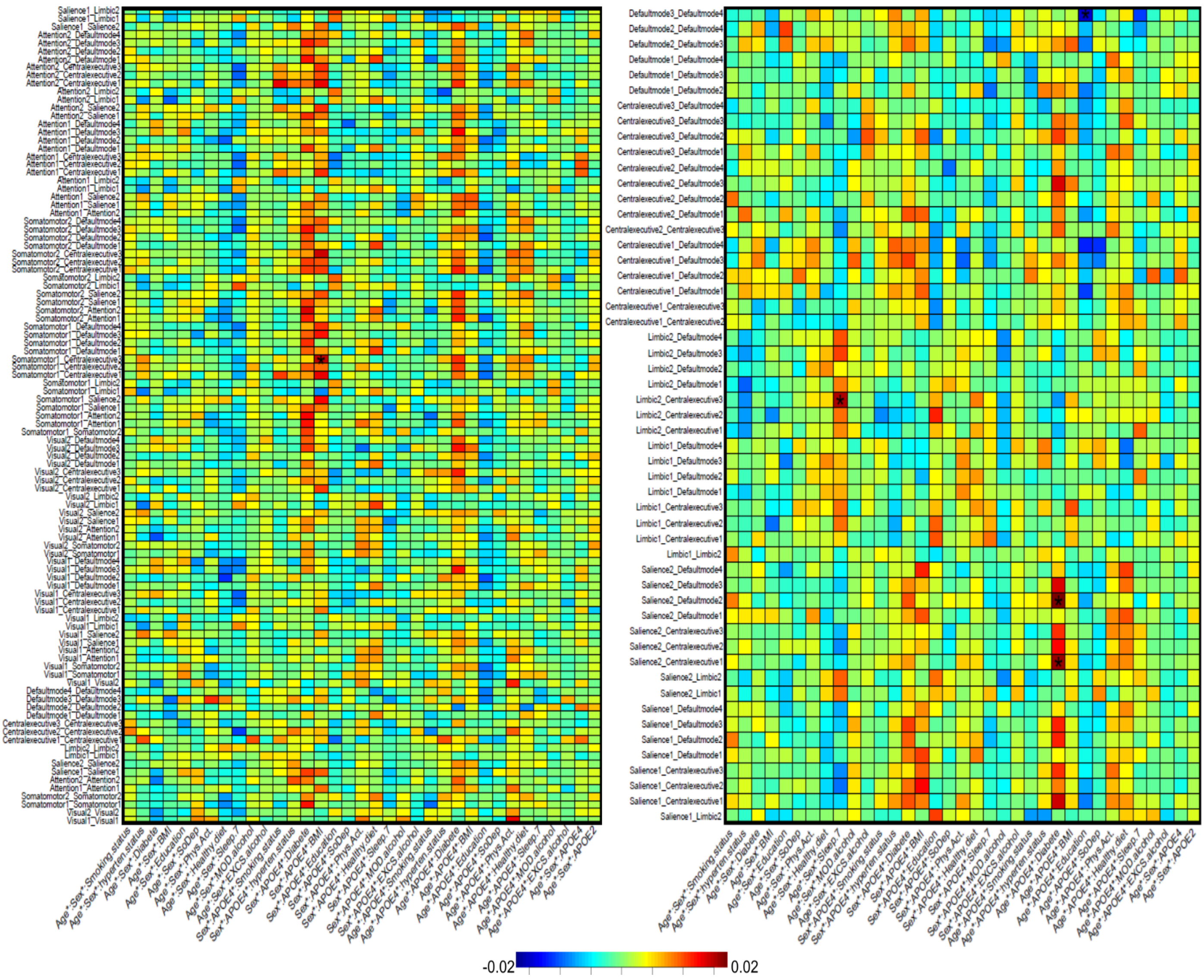

Supplementary Figure 19. Heatmap of all the identified three-way interaction effects of demographic, lifestyle, social economic status (SES) factors, and cardiovascular risk factors (CVRF) on the network functional connectivity (NFC) measures, based on the Yeo-17 network atlas. Results that passed the Bonferroni-corrected significance level of *3.3E-4* will be shown by (*). The effect sizes (standardized coefficients) for each association are illustrated using a color gradient from blue to red.

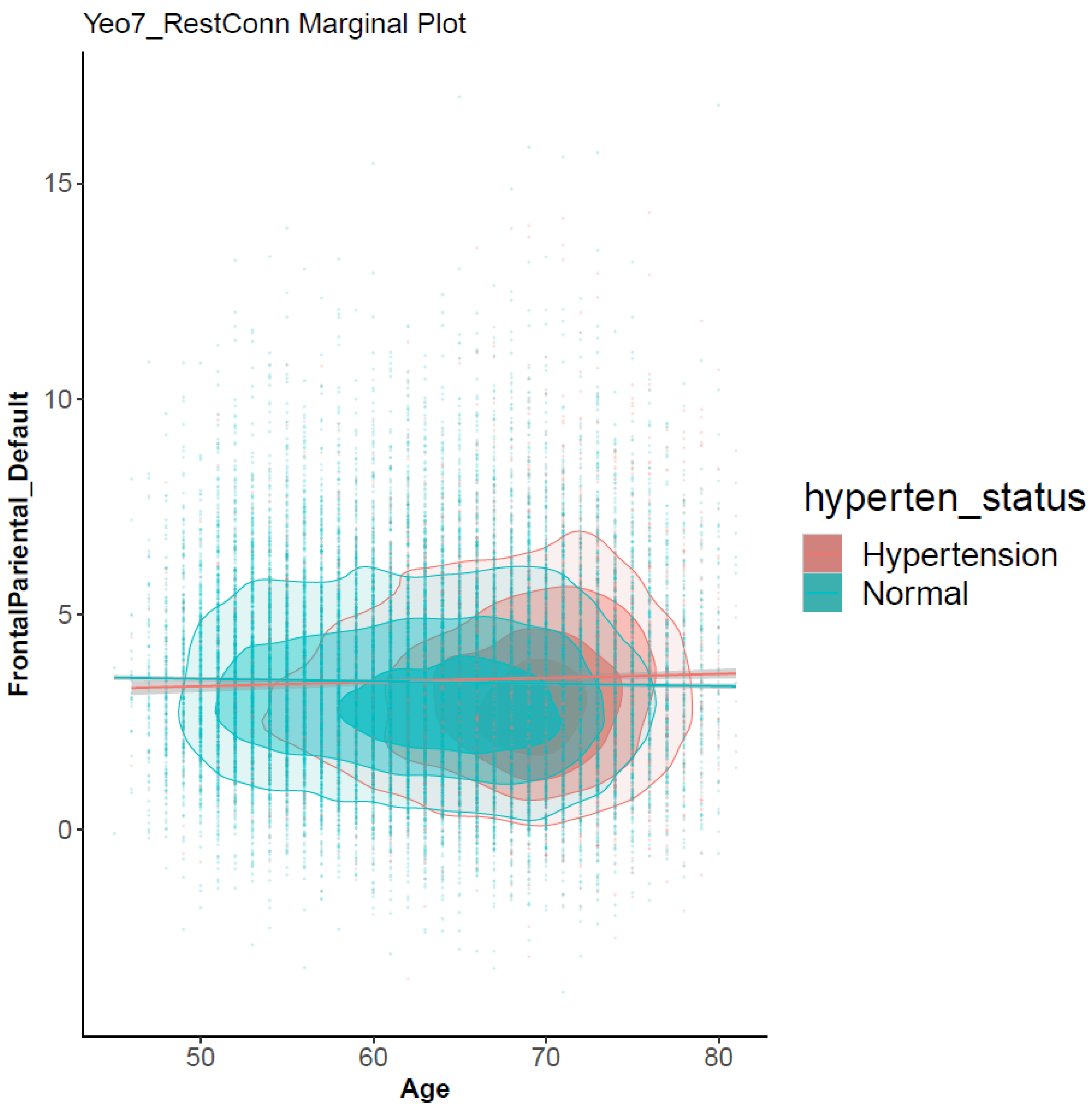

Supplementary Figure S20 The scatterplots of FPN-DMN between-network functional connectivity (NFC) against age grouped by hypertension. Results are based on the Yeo-7 network atlas. Contour plots are used to show the density of two-dimensional distributions; regression lines and 95% confidence intervals were also drawn. FPN, frontoparietal network; DMN, default mode network.

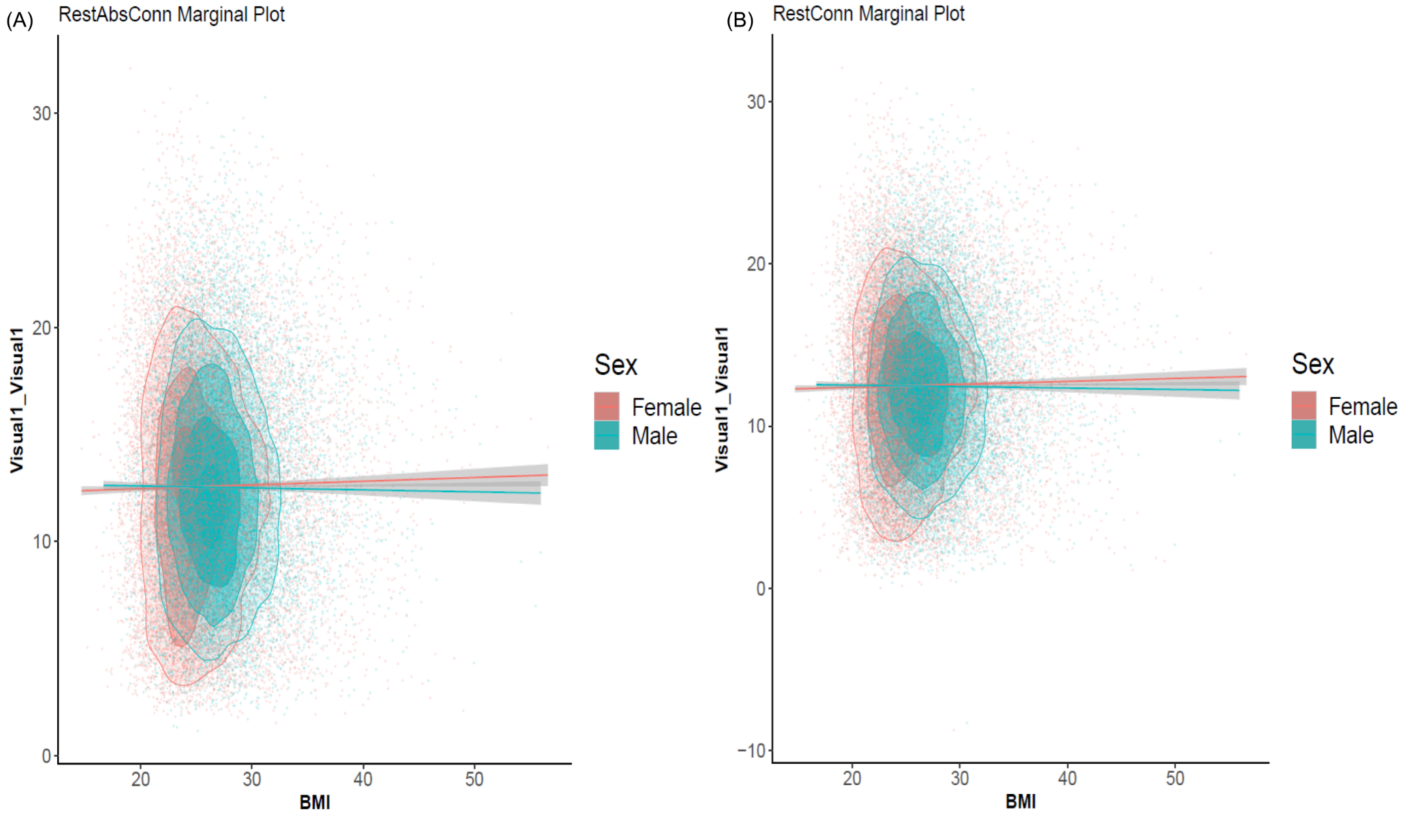

Supplementary Figure S21 The scatterplots of (A) within-Vi1-network edge strength (NES) and (B) network functional connectivity (NFC) against BMI grouped by sex. Results are based on the Ji-12 network atlas. Contour plots are used to show the density of two-dimensional distributions; regression lines and 95% confidence intervals were also drawn. Vi1, visual 1 network.

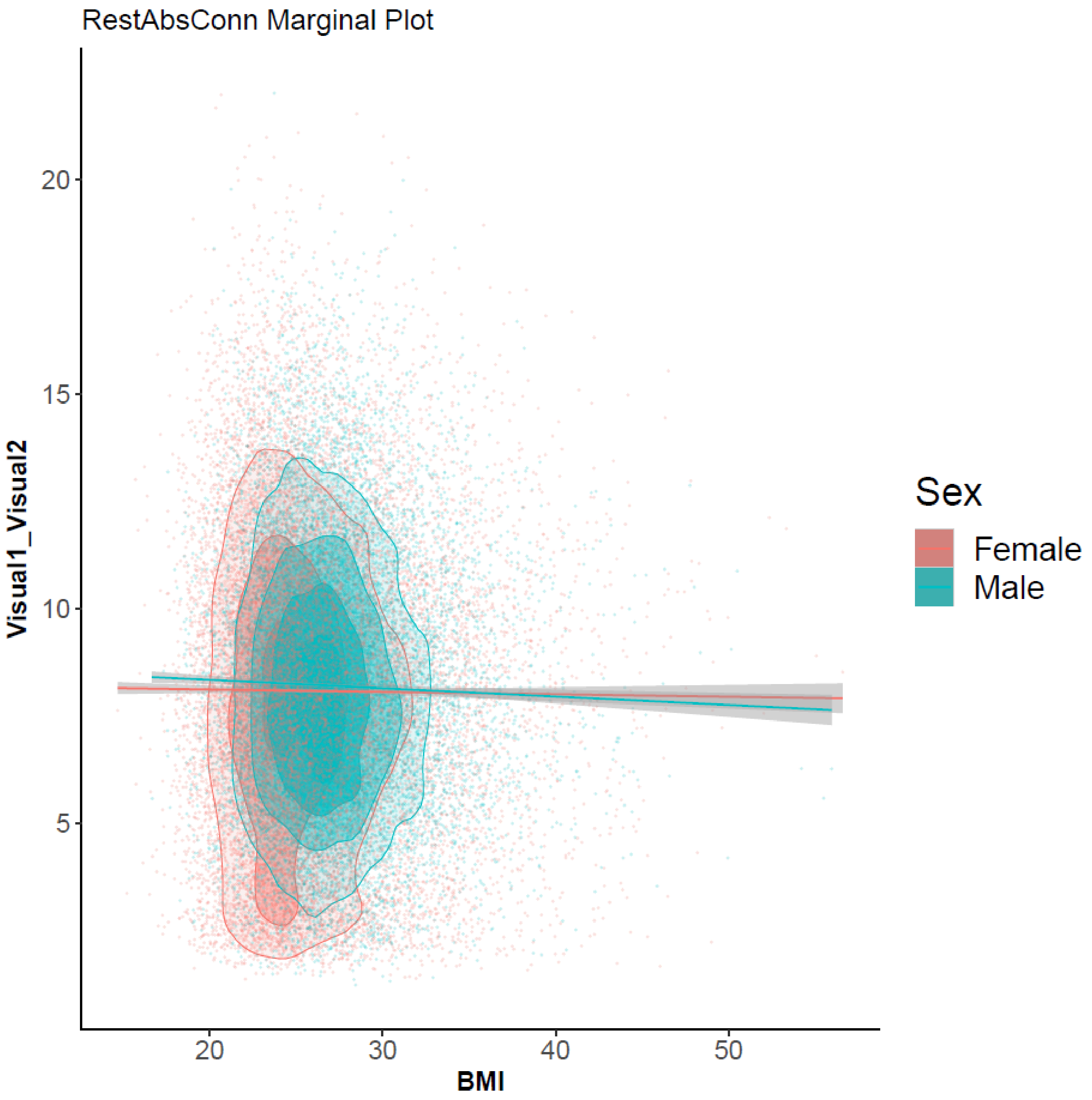

Supplementary Figure S22 The scatterplots of Vi1-Vi2 between-network edge strength (NES) against BMI grouped by sex. Results are based on the Ji-12 network atlas. Contour plots are used to show the density of two-dimensional distributions; regression lines and 95% confidence intervals were also drawn. Vi1, visual 1 network; Vi2, visual 2 network.

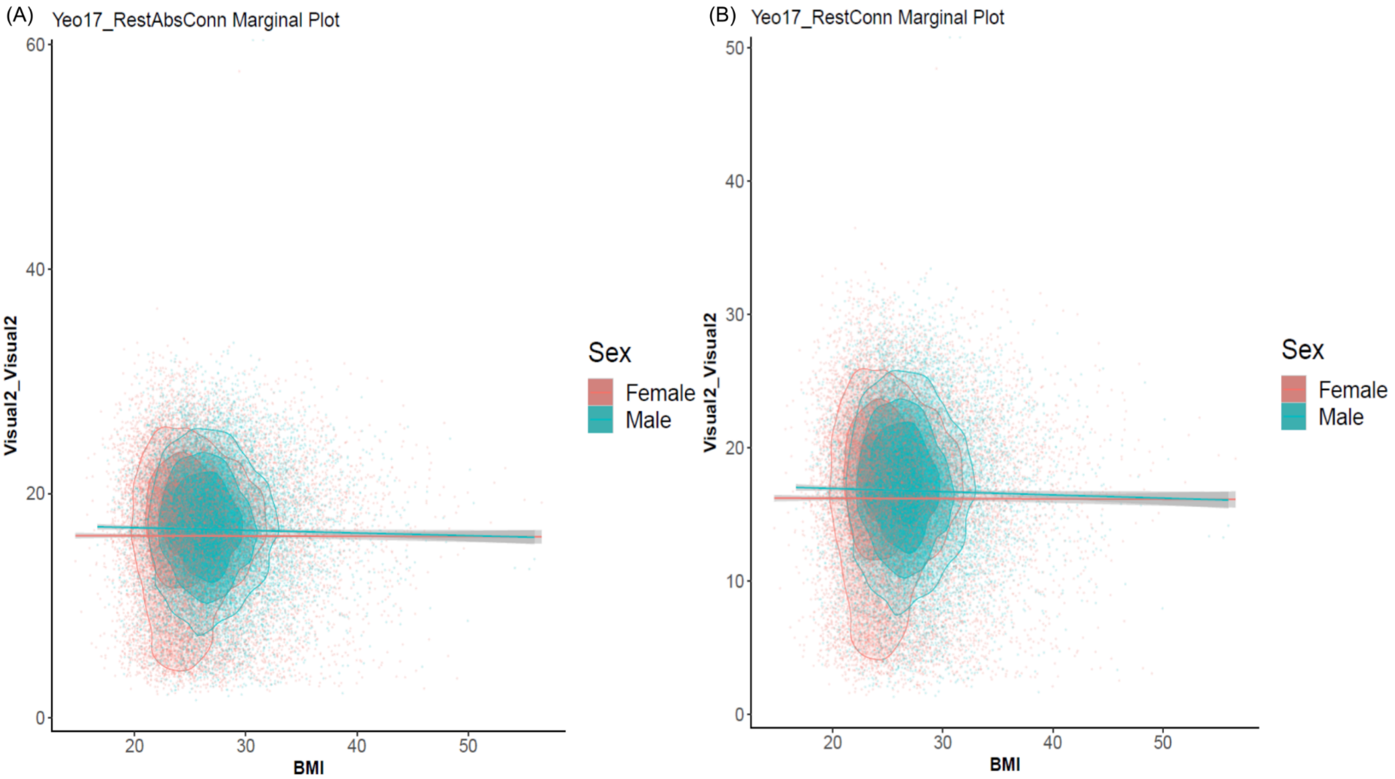

Supplementary Figure S23 The scatterplots of (A) within-Vi2 network edge strength (NES) and (B) network functional connectivity (NFC) against BMI grouped by sex. Results are based on the Yeo-17 network atlas. Contour plots are used to show the density of two-dimensional distributions; regression lines and 95% confidence intervals were also drawn. Vi2, visual 2 network.

intervals were also drawn.

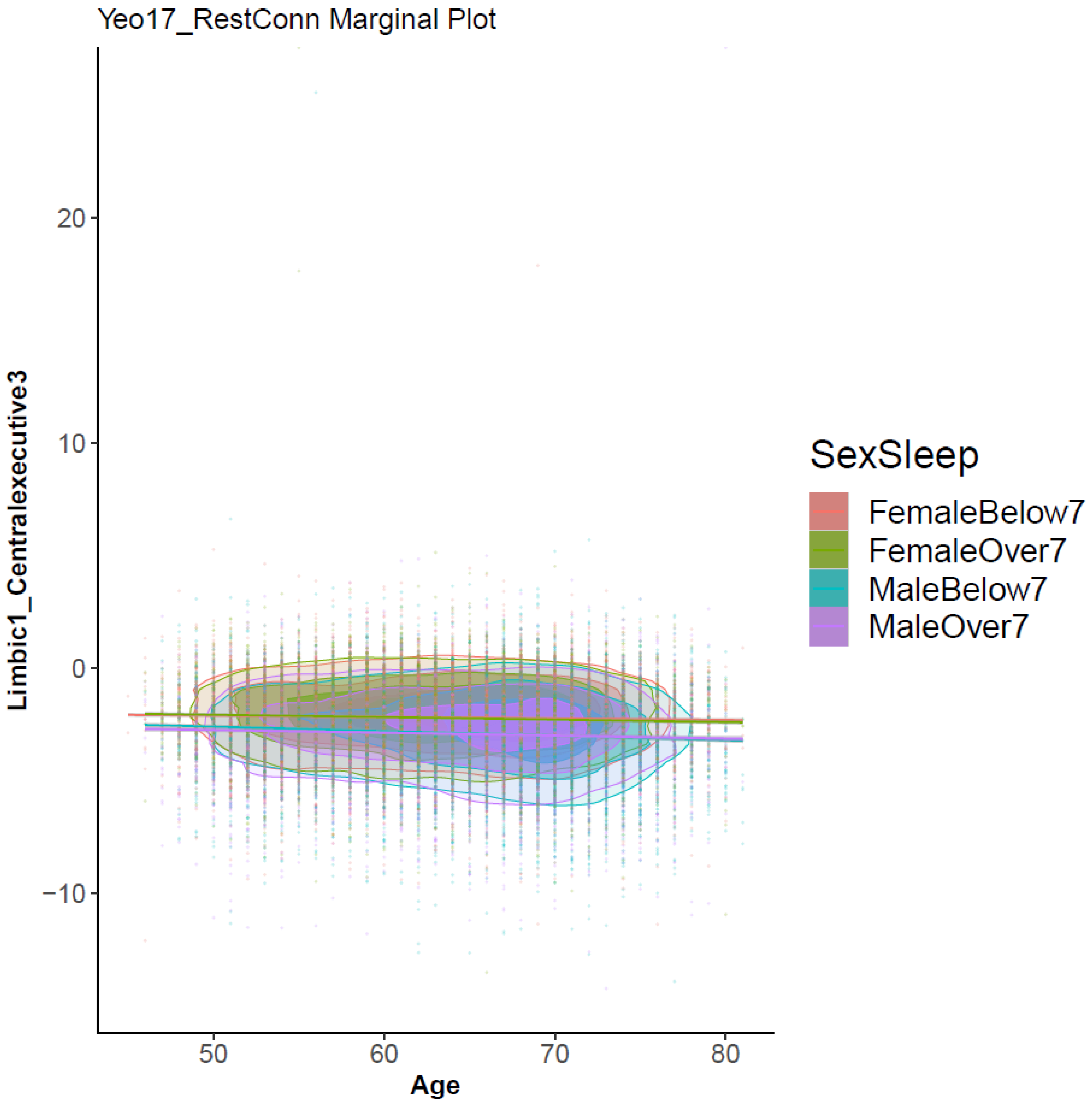

Supplementary Figure S24 The scatterplots of LN1-CE3 between-network functional connectivity (NFC) against age grouped by sex. Results are based on the Yeo-17 network atlas. Contour plots are used to show the density of two-dimensional distributions; regression lines and 95% confidence intervals were also drawn. LN1, limbic 1 network; CE3, central executive 3 network.

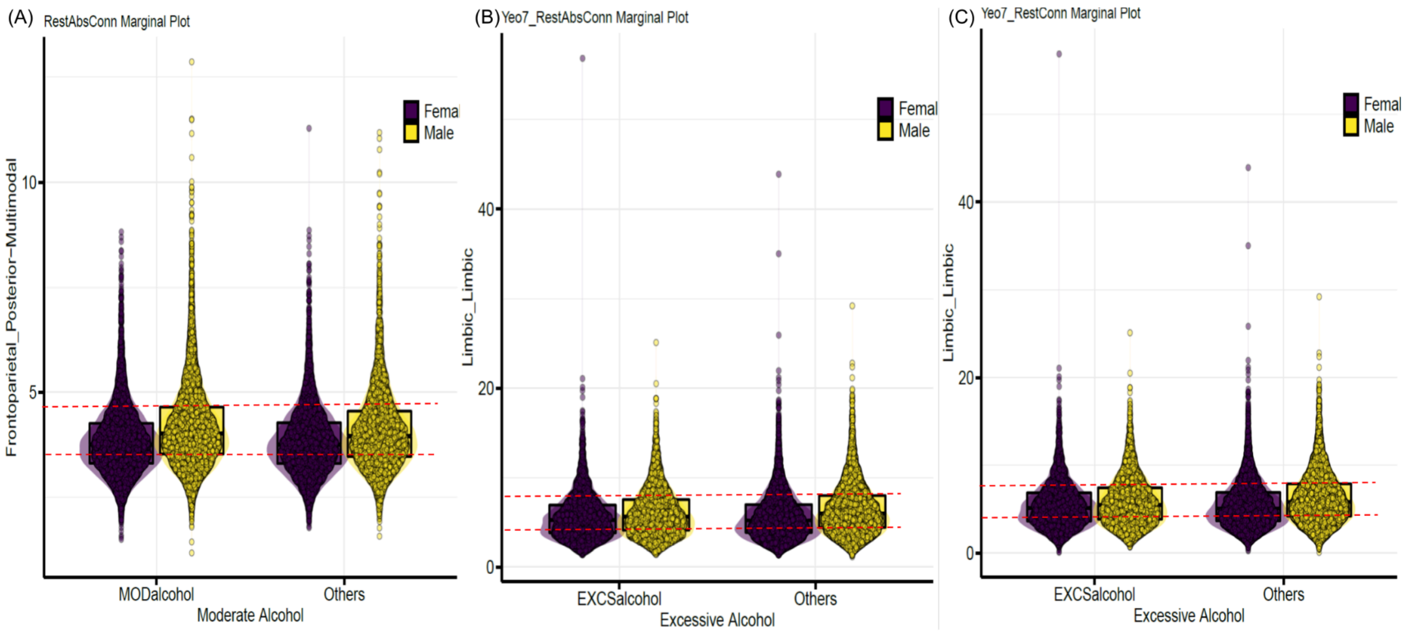

Supplementary Figure S25 The boxplots of (A) FPN-PMN between-network edge strength (NES), (B) within-LN NES and (C) within-LN network functional connectivity (NFC) against different alcohol consumptions grouped by sex. Results are based on the Ji-12, Yeo-7 and Yeo-7 network atlases, respectively. FPN, frontoparietal network; PMN, posterior multimodal network; LN, limbic network.

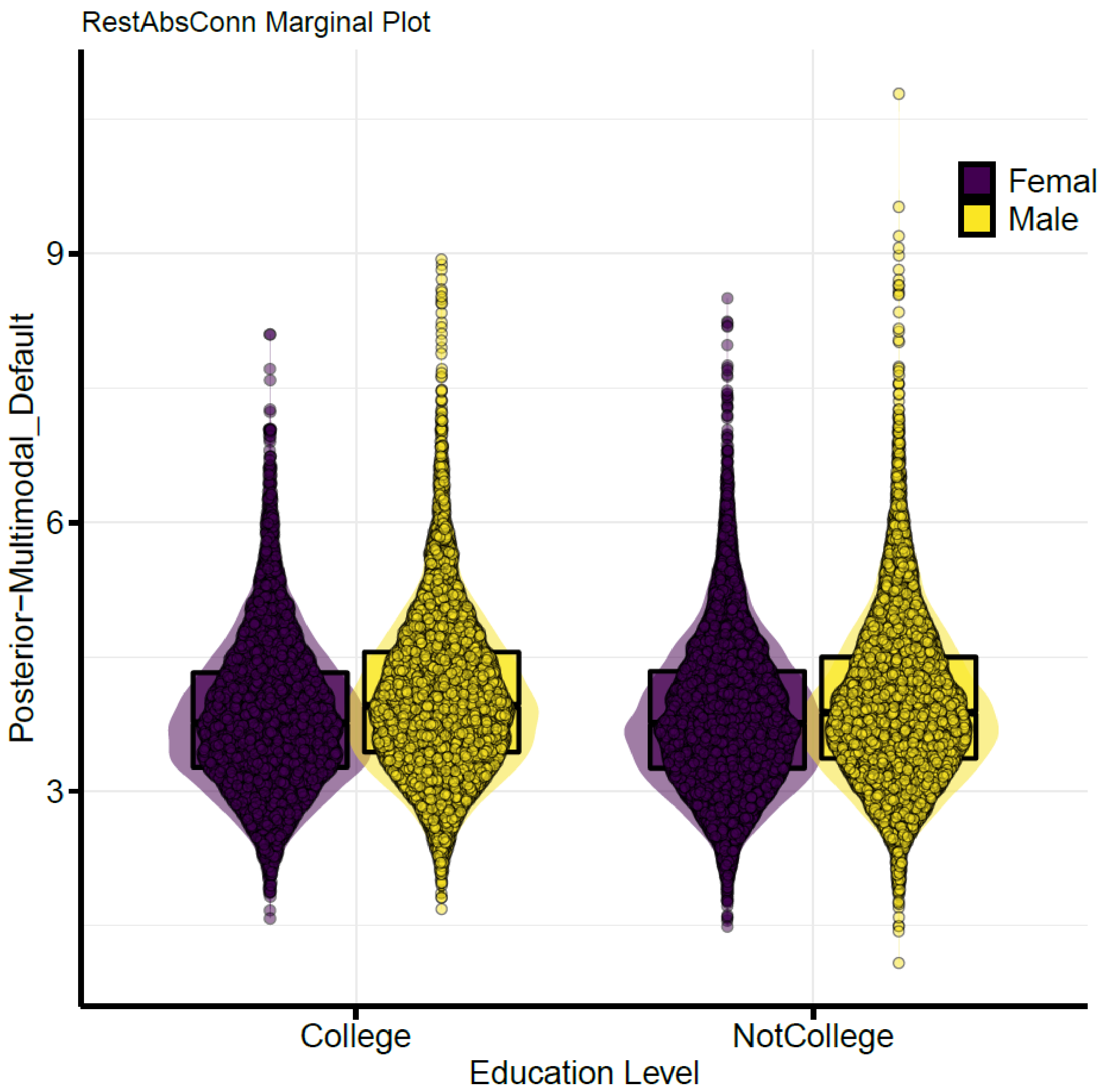

Supplementary Figure S26 The boxplots of PMN-DAN between-network edge strength (NES) against different education levels (with or without college degree) grouped by sex. Results are based on the Ji-12 network atlas. PMN, posterior multimodal network; DAN, dorsal attention network.

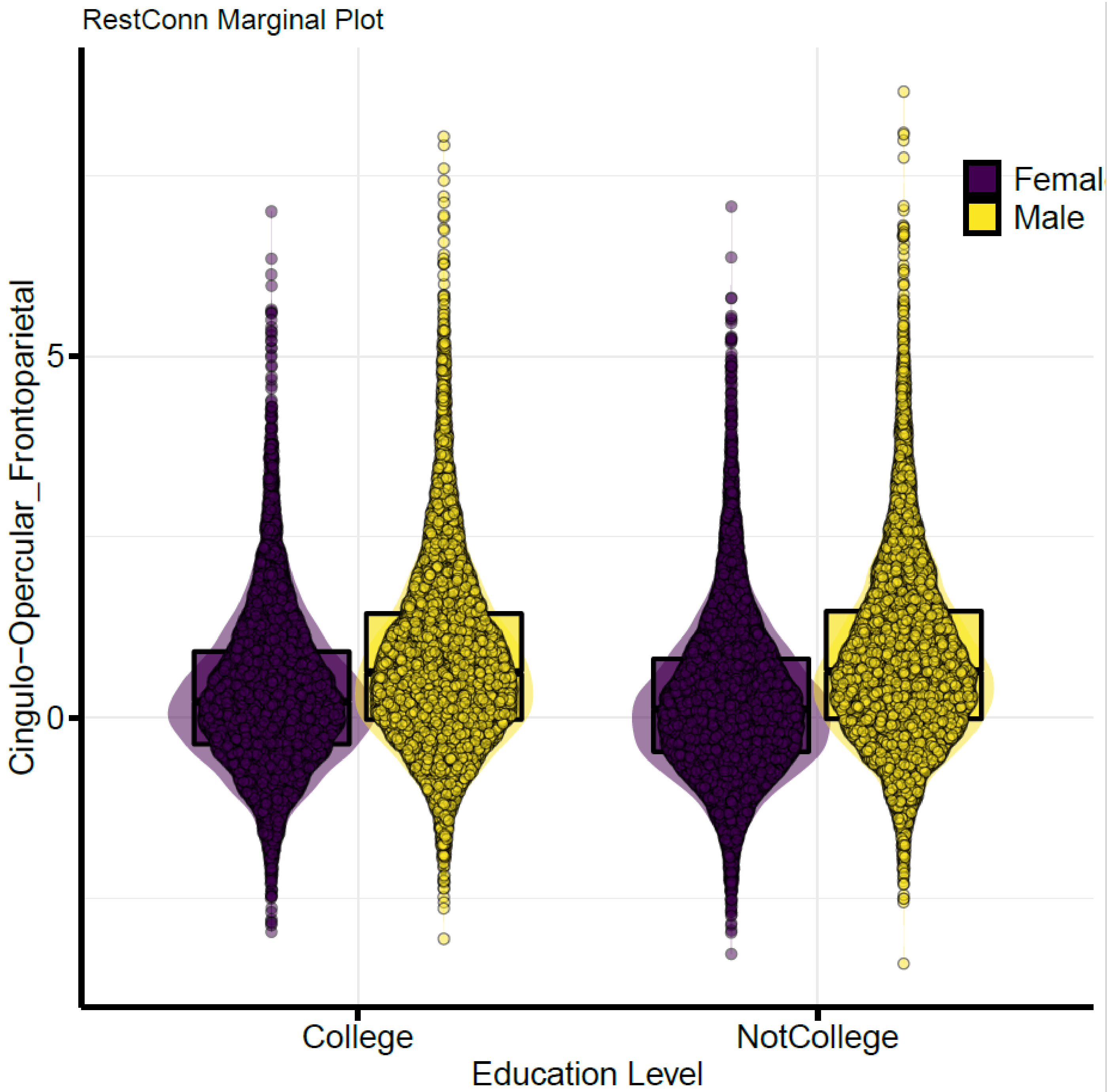

Supplementary Figure S27 The boxplot of CON-FPN between-network functional connectivity (NFC) against different education levels (with or without college degree) grouped by sex. Results are based on the Ji-12 network atlas. CON, cingulo-Opercular network; FPN, frontoparietal network.

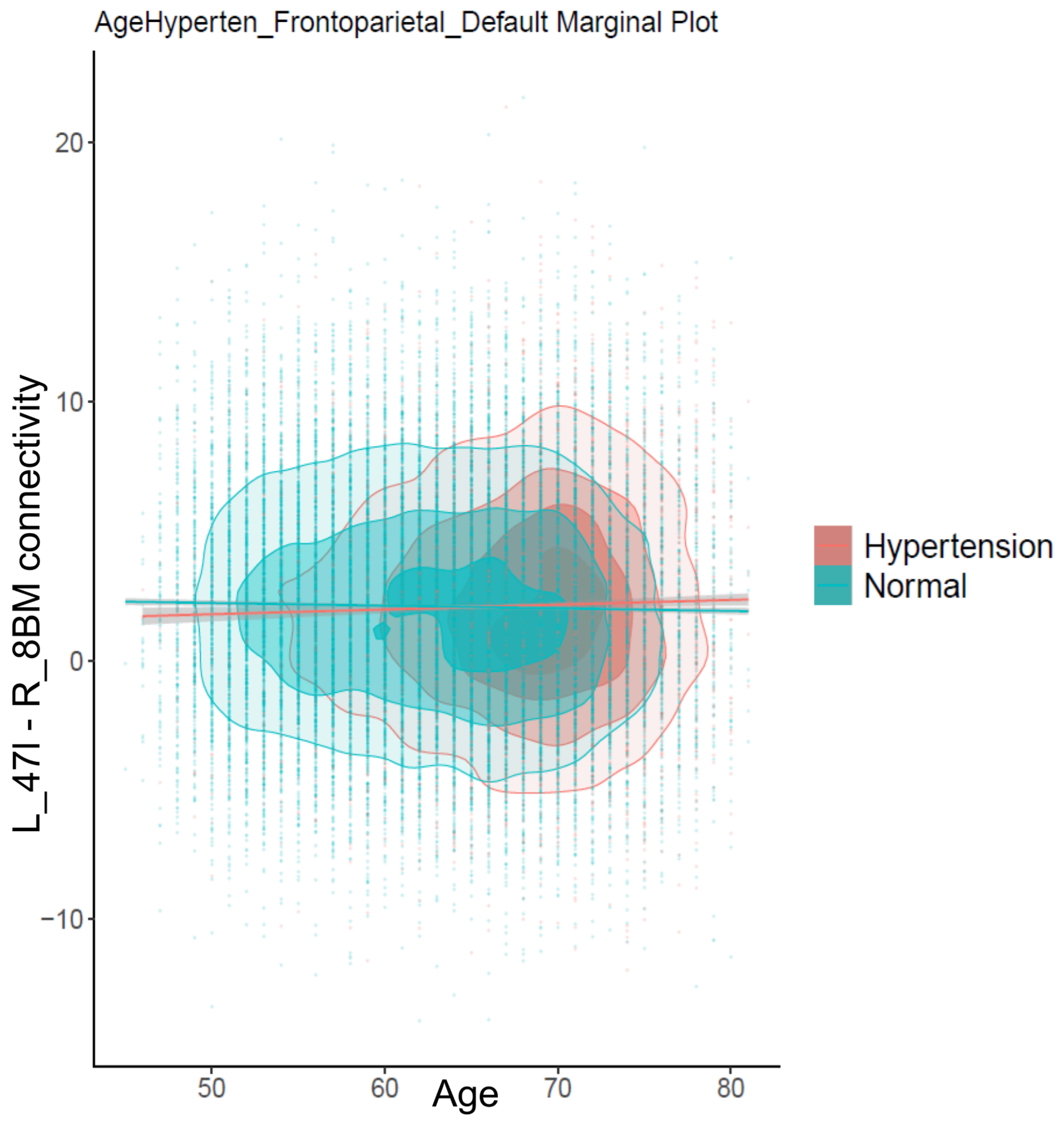

Supplementary Figure S28 The scatterplots of brain region L_47l and R_8BM connectivity against age grouped by hypertension status. Contour plots are used to show the density of two-dimensional distributions; regression lines and 95% confidence intervals were also drawn.

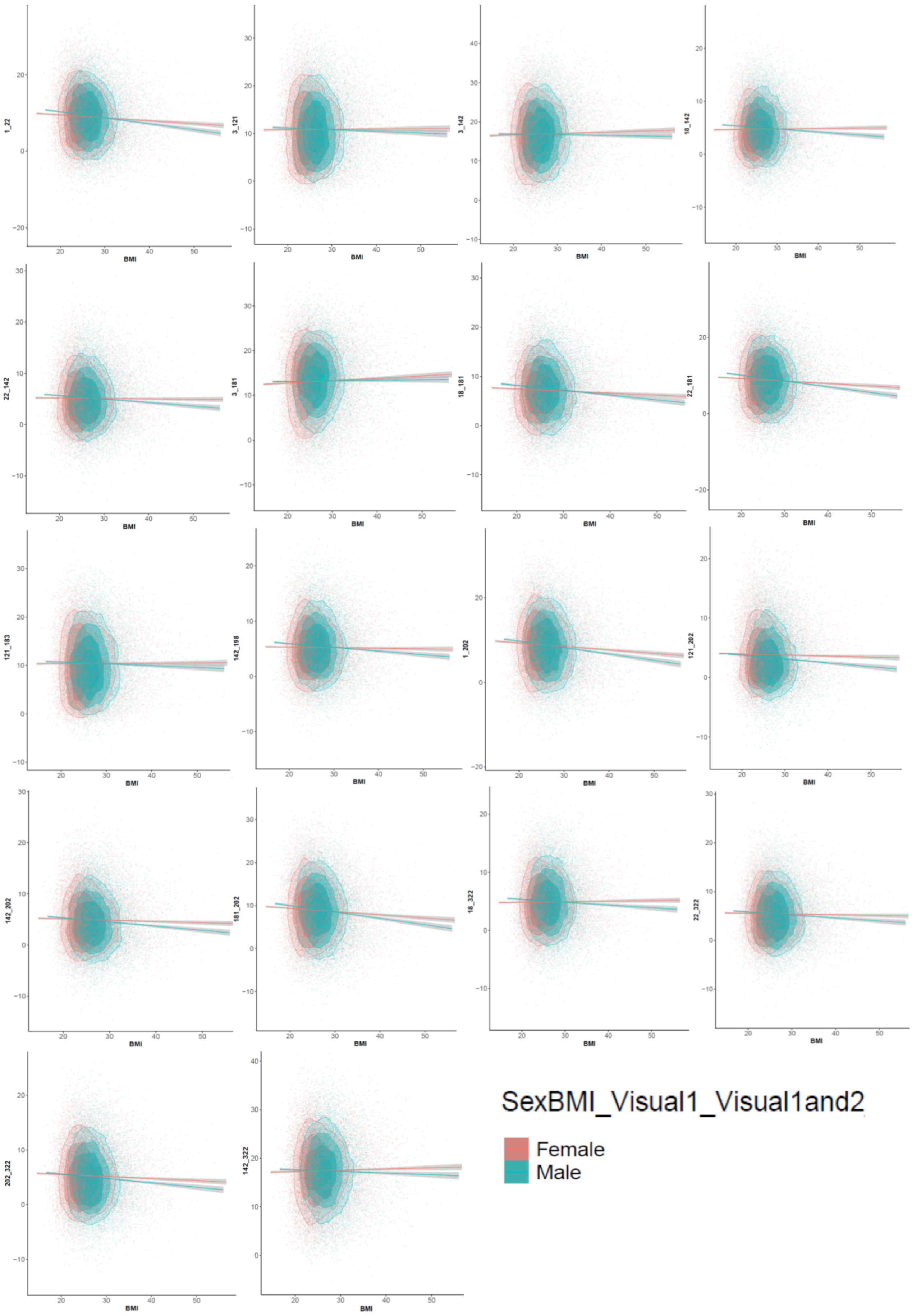

Supplementary Figure S29 The scatterplots of brain functional connectivities between the 18 pairs of brain regions in brain visual regions (Vi1 and Vi2; Ji-12 network atlas) against BMI grouped by sex, respectively. Contour plots are used to show the density of two-dimensional distributions; regression lines and 95% confidence intervals were also drawn. Vi1, visual 1 network; Vi2, visual 2 network.

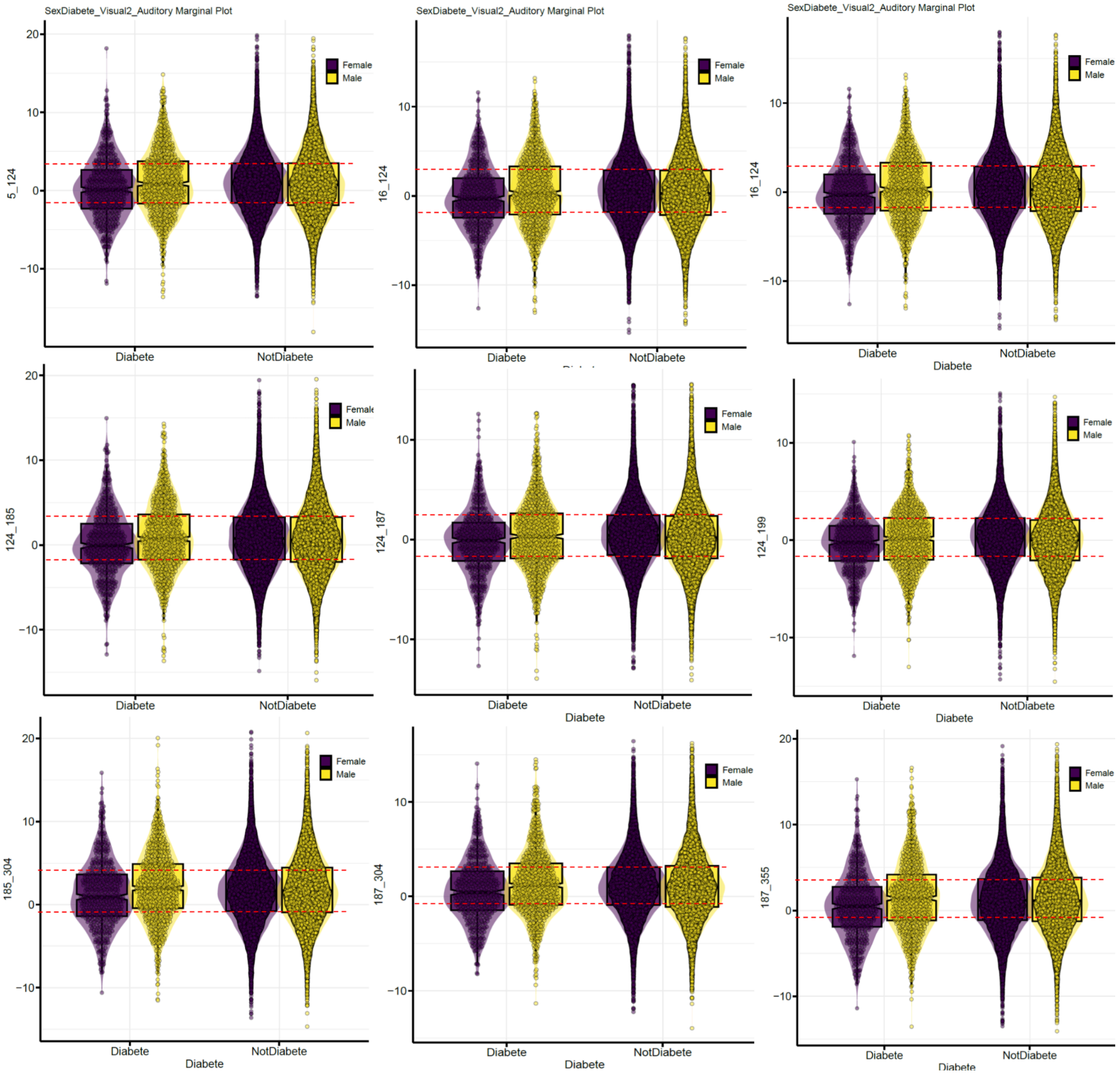

Supplementary Figure S30 The boxplots of the brain functional connectivities between 9 pairs of brain regions from brain Visual 2 and auditory networks for subjects with and without diabetes (Ji-12 network atlas).

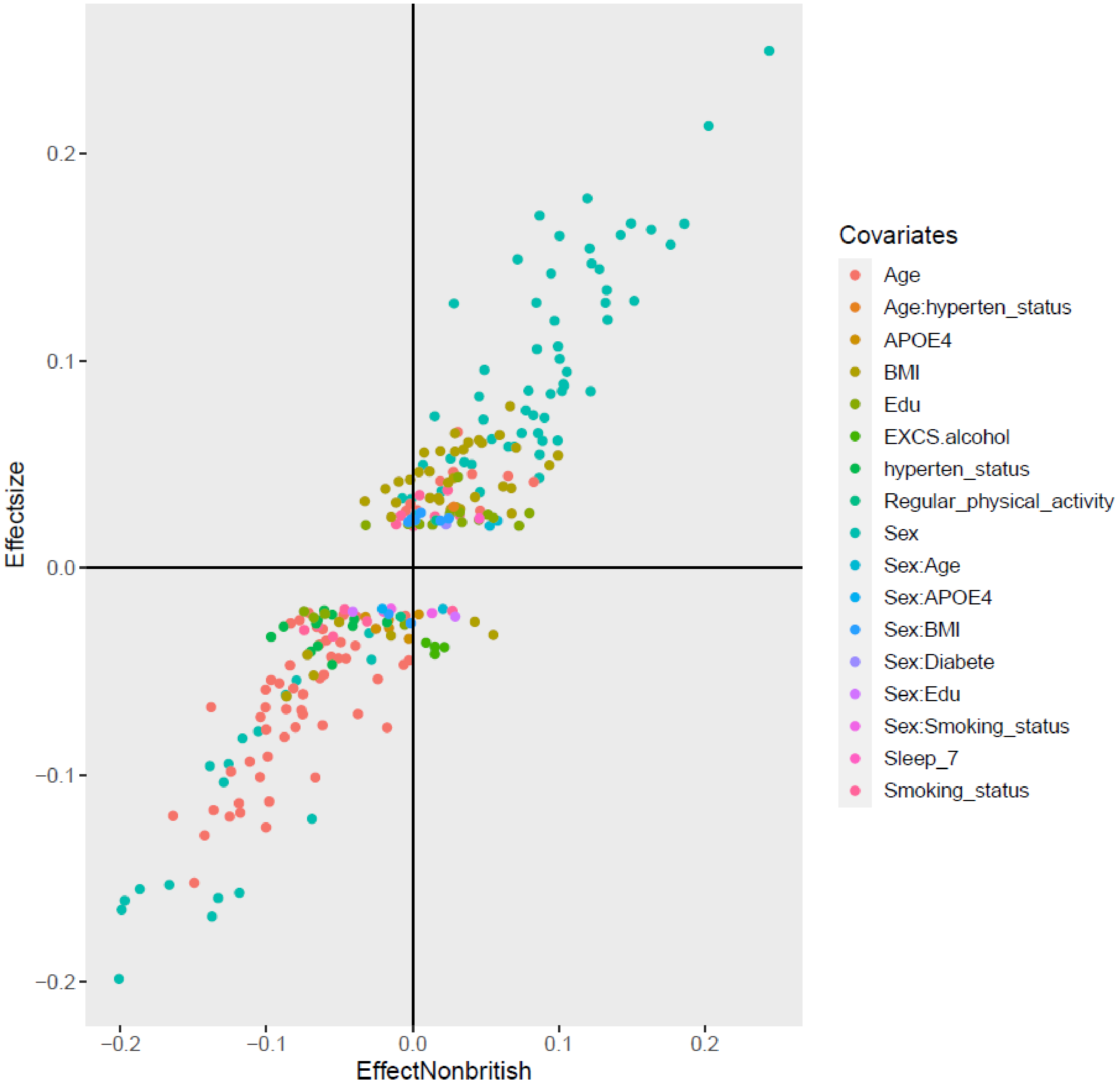

Supplementary Figure S31 Plot of effects on network functional connectivities (NFCs) identified based on the Bonferroni-corrected significance level in the main discovery (n = 33,824) versus their corresponding effect sizes in non-British dataset (n = 2806) based on the Ji-12 network atlas. The identified effect sizes were reproducible, with a Pearson correlation of *0.92* (Spearman correlation = *0.90*).

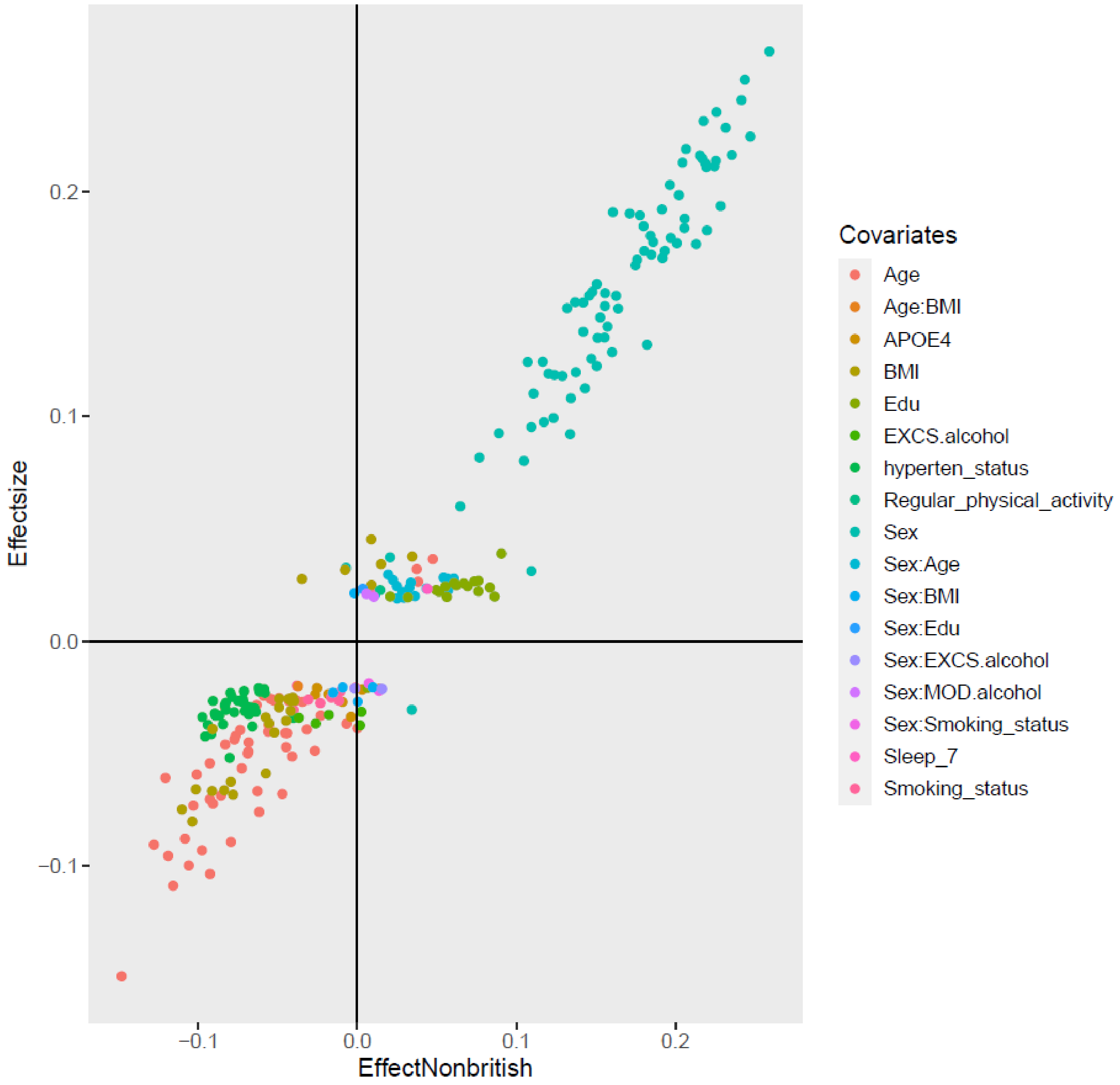

Supplementary Figure S32 Plot of effects on network edge strength (NES) measures identified based on the Bonferroni-corrected significance level in the main discovery (n = 33,824) versus their corresponding effect sizes in non-British dataset (n = 2806) based on the Ji-12 network atlas. The identified effect sizes were reproducible, with a Pearson correlation of *0.97* (Spearman correlation = *0.94*).

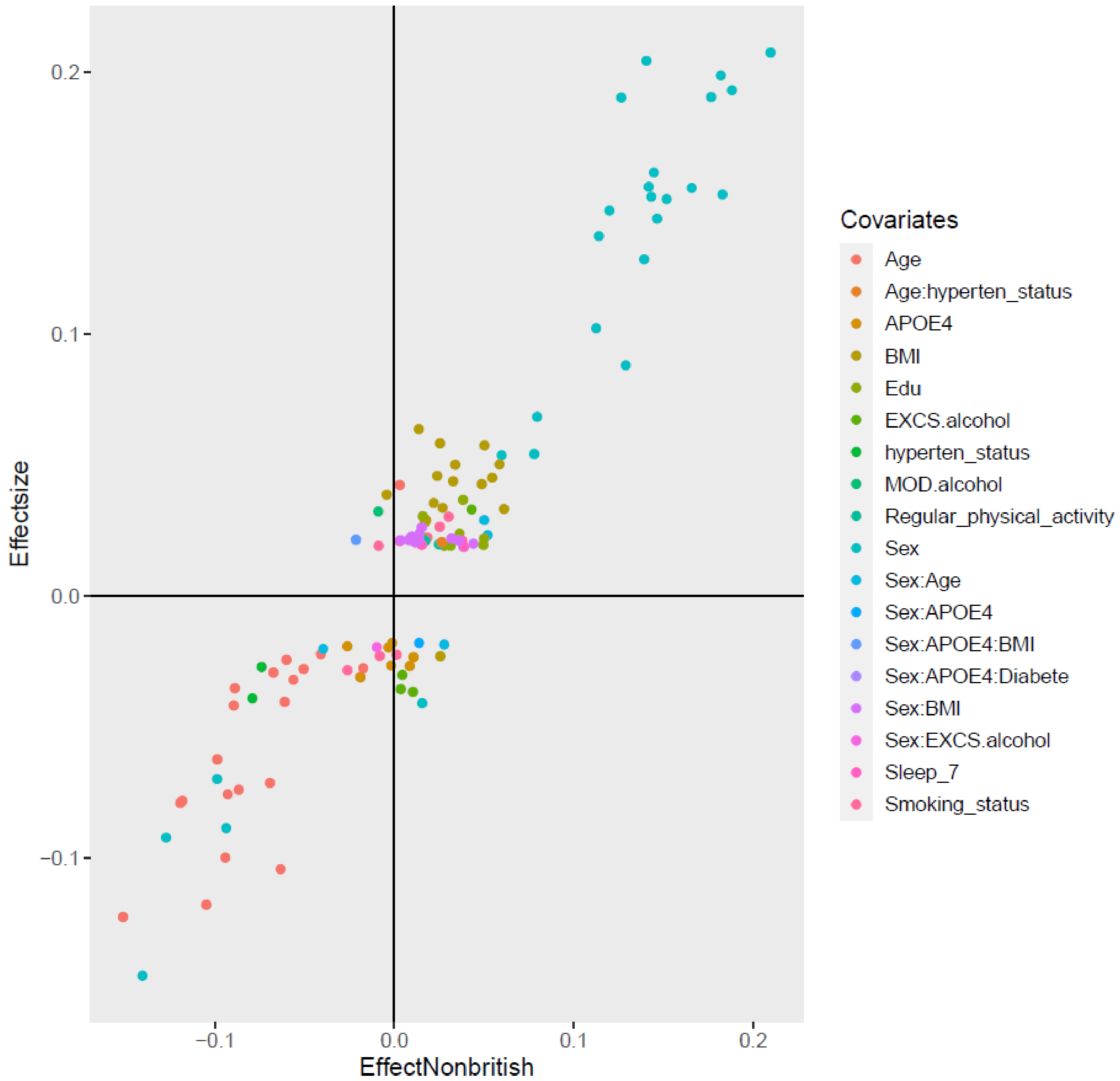

Supplementary Figure S33 Plot of effects on network functional connectivities (NFCs) identified based on the Bonferroni-corrected significance level in the main discovery (n = 33,824) versus their corresponding effect sizes in non-British dataset (n = 2806) based on the Yeo-7 network atlas. The identified effect sizes were reproducible, with a Pearson correlation of *0.94* (Spearman correlation = *0.87*).

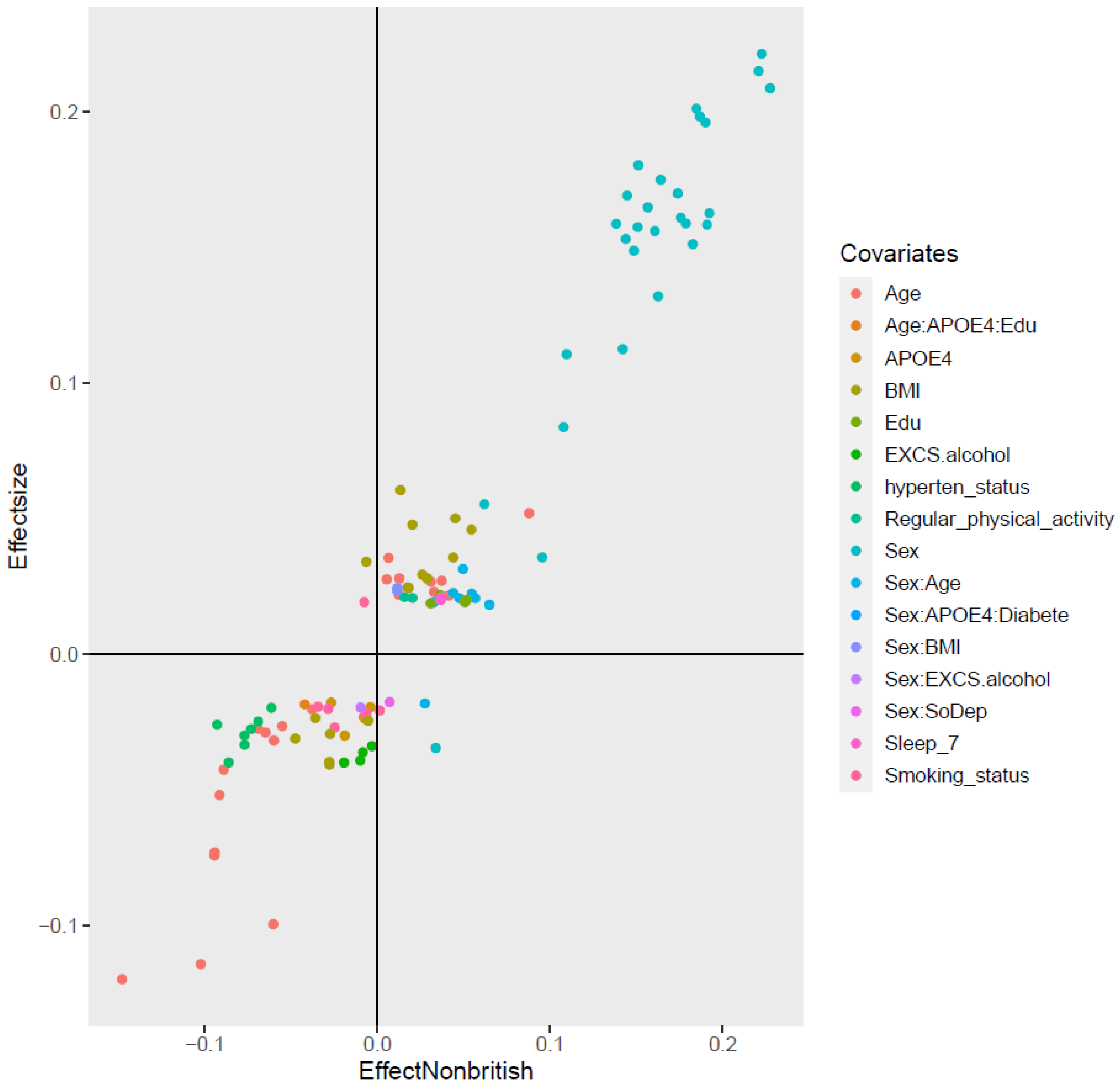

Supplementary Figure S34 Plot of effects on network edge strength (NES) measures identified based on the Bonferroni-corrected significance level in the main discovery (n = 33,824) versus their corresponding effect sizes in non-British dataset (n = 2806) based on the Yeo-7 network atlas. The identified effect sizes were reproducible, with a Pearson correlation of *0.95* (Spearman correlation = *0.89*).

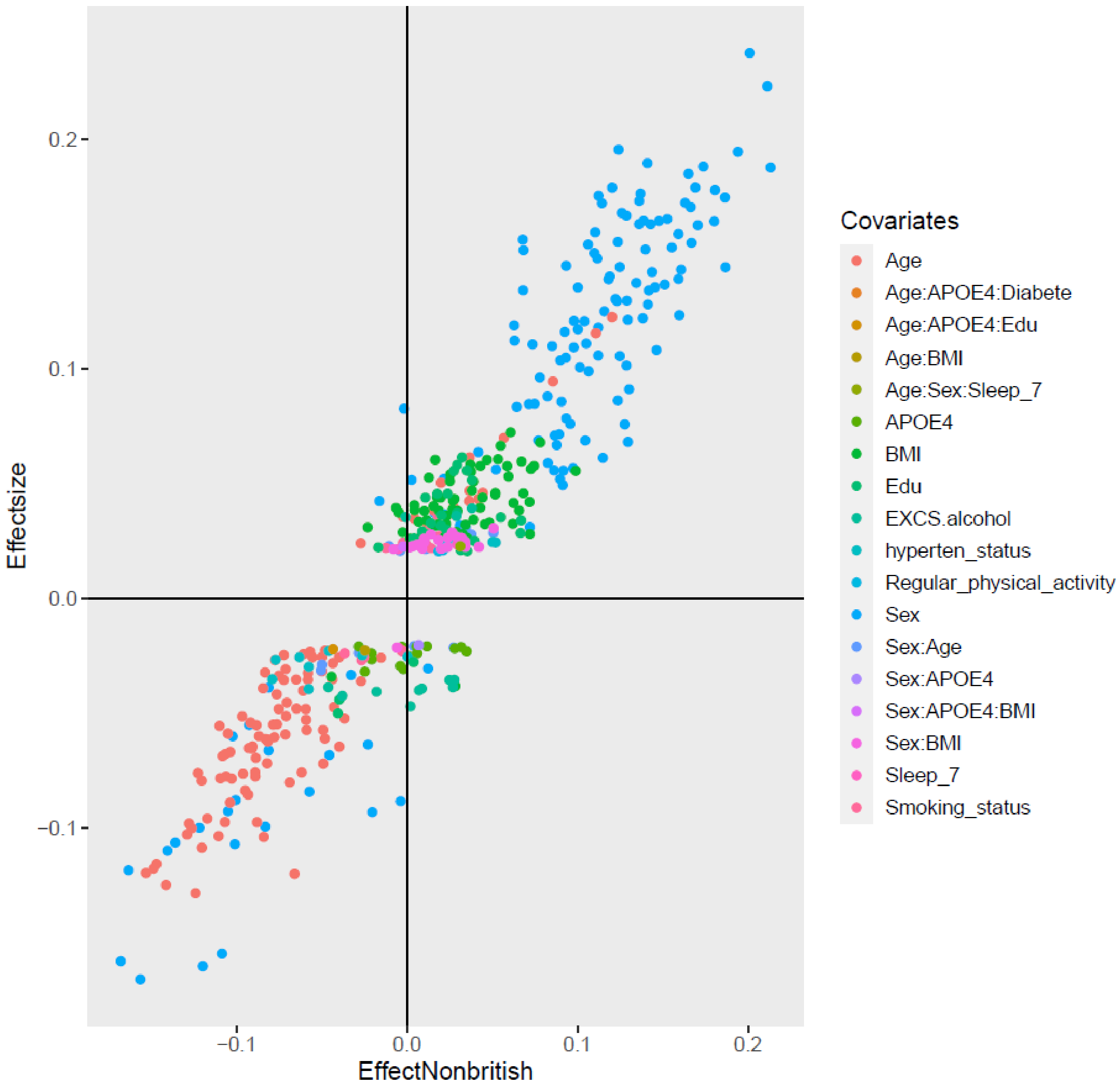

Supplementary Figure S35 Plot of effects on network functional connectivities (NFCs) identified based on the Bonferroni-corrected significance level in the main discovery (n = 33,824) versus their corresponding effect sizes in non-British dataset (n = 2806) based on the Yeo-17 network atlas. The identified effect sizes were reproducible, with a Pearson correlation of *0.94* (Spearman correlation = *0.93*).

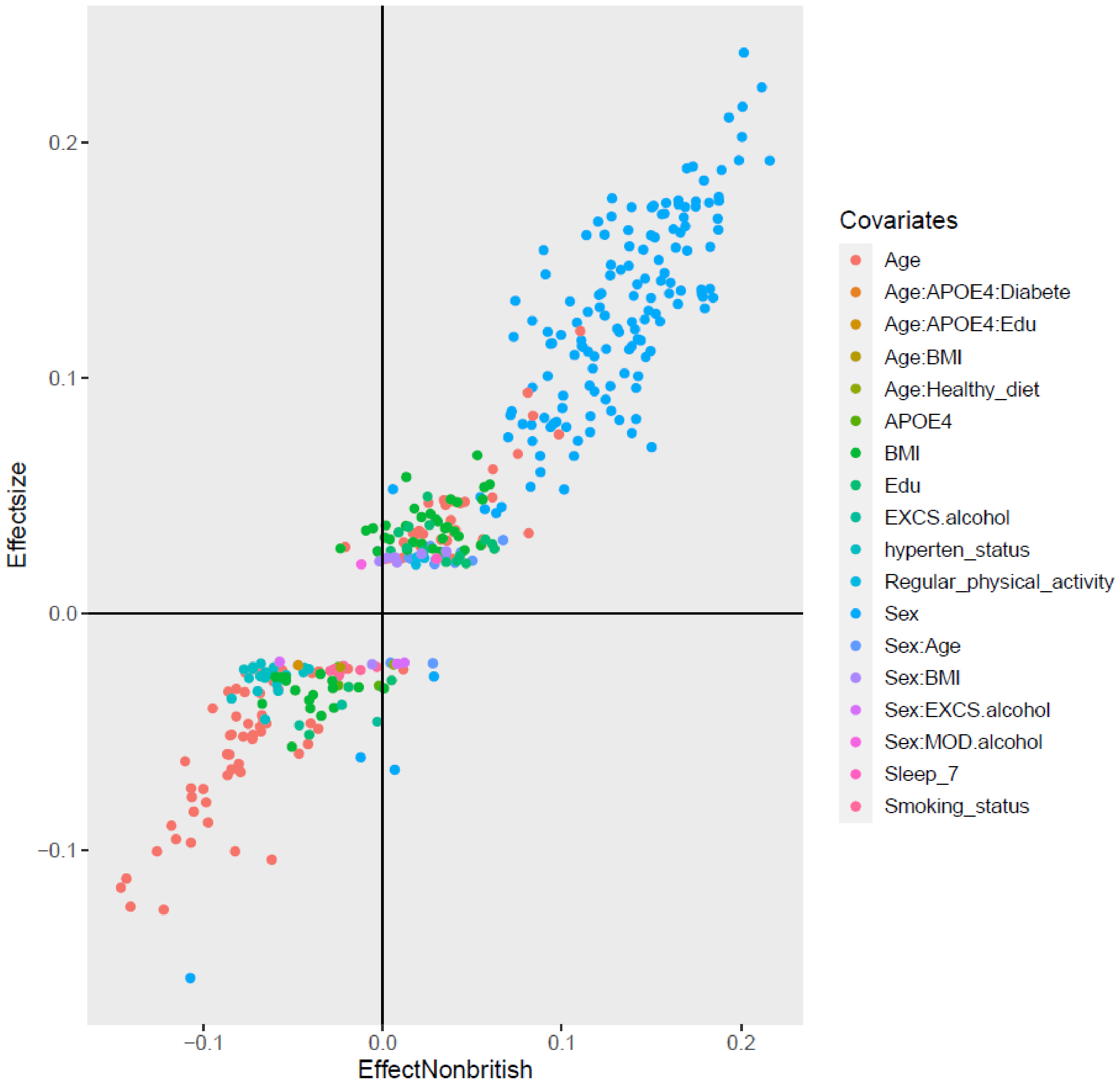

Supplementary Figure S36 Plot of effects on network edge strength (NES) measures identified based on the Bonferroni-corrected significance level in the main discovery (n = 33,824) versus their corresponding effect sizes in non-British dataset (n = 2806) based on the Yeo-17 network atlas. The identified effect sizes were reproducible, with a Pearson correlation of *0.96* (Spearman correlation = *0.95*).

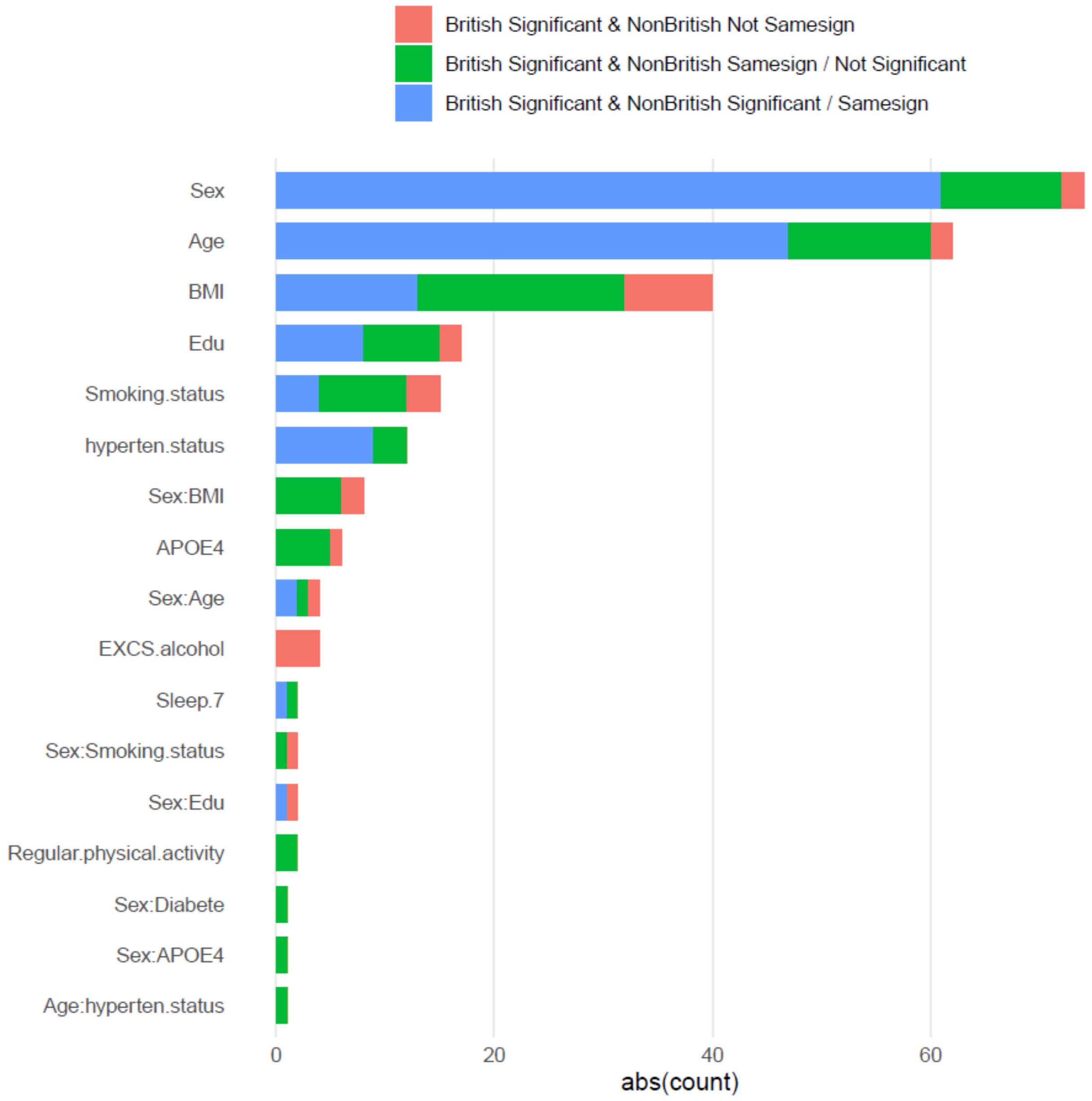

Supplementary Figure S37 Barplots of number of signals based on the Bonferroni significance level in the main discovery (n = 33,824) on network functional connectivities (NFCs) based on the Ji-12 network atlas that have inconsistent directions (red) in non-British dataset (n = 2806), have consistent directions that are significant (blue) or non-significant (green).

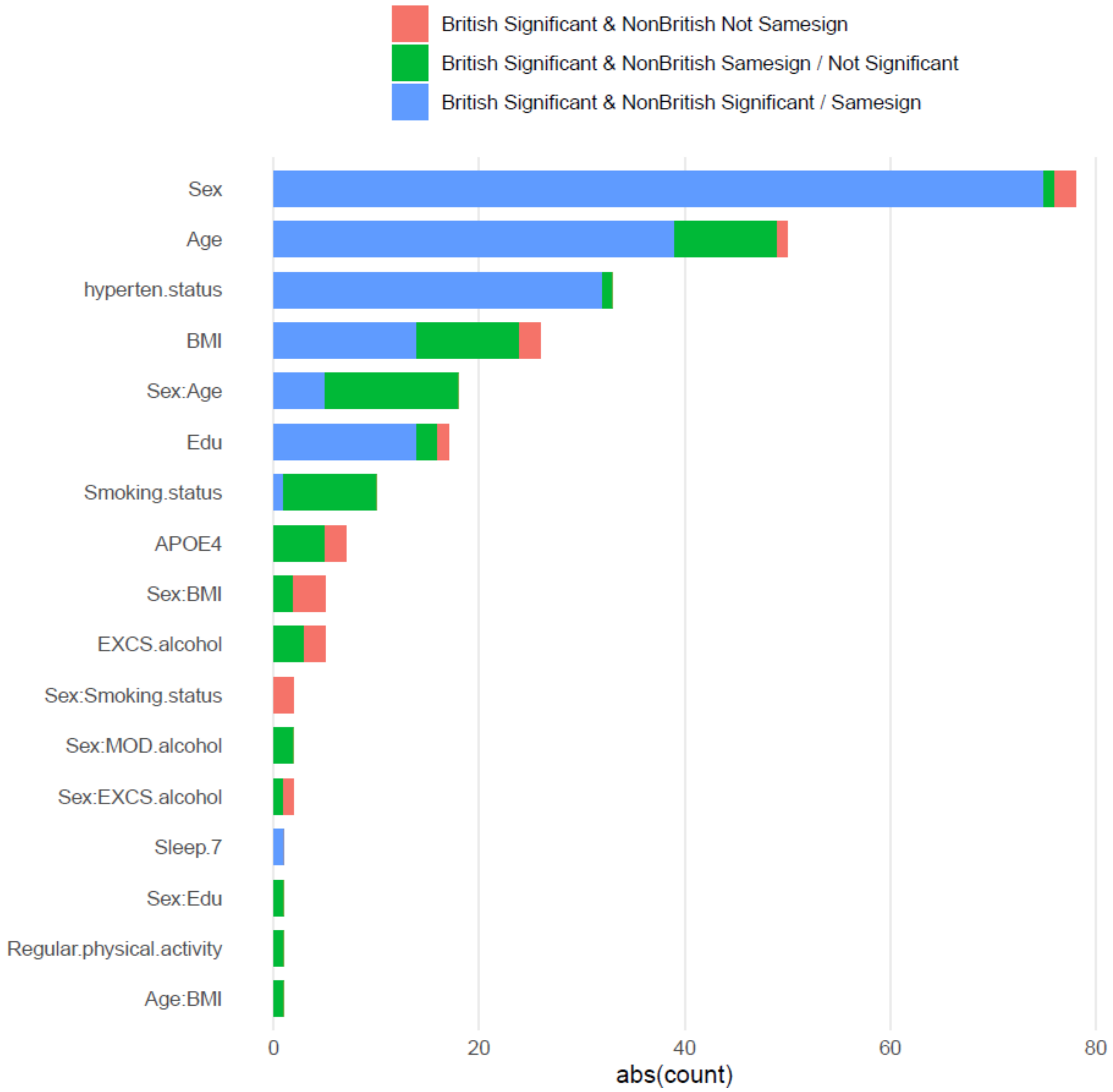

Supplementary Figure S38 Barplots of number of signals based on the Bonferroni significance level in the main discovery (n = 33,824) on network edge strength (NES) measures based on the Ji-12 network atlas that have inconsistent directions (blue) in non-British dataset (n = 2806), have consistent directions that are significant (green) or non-significant (red).

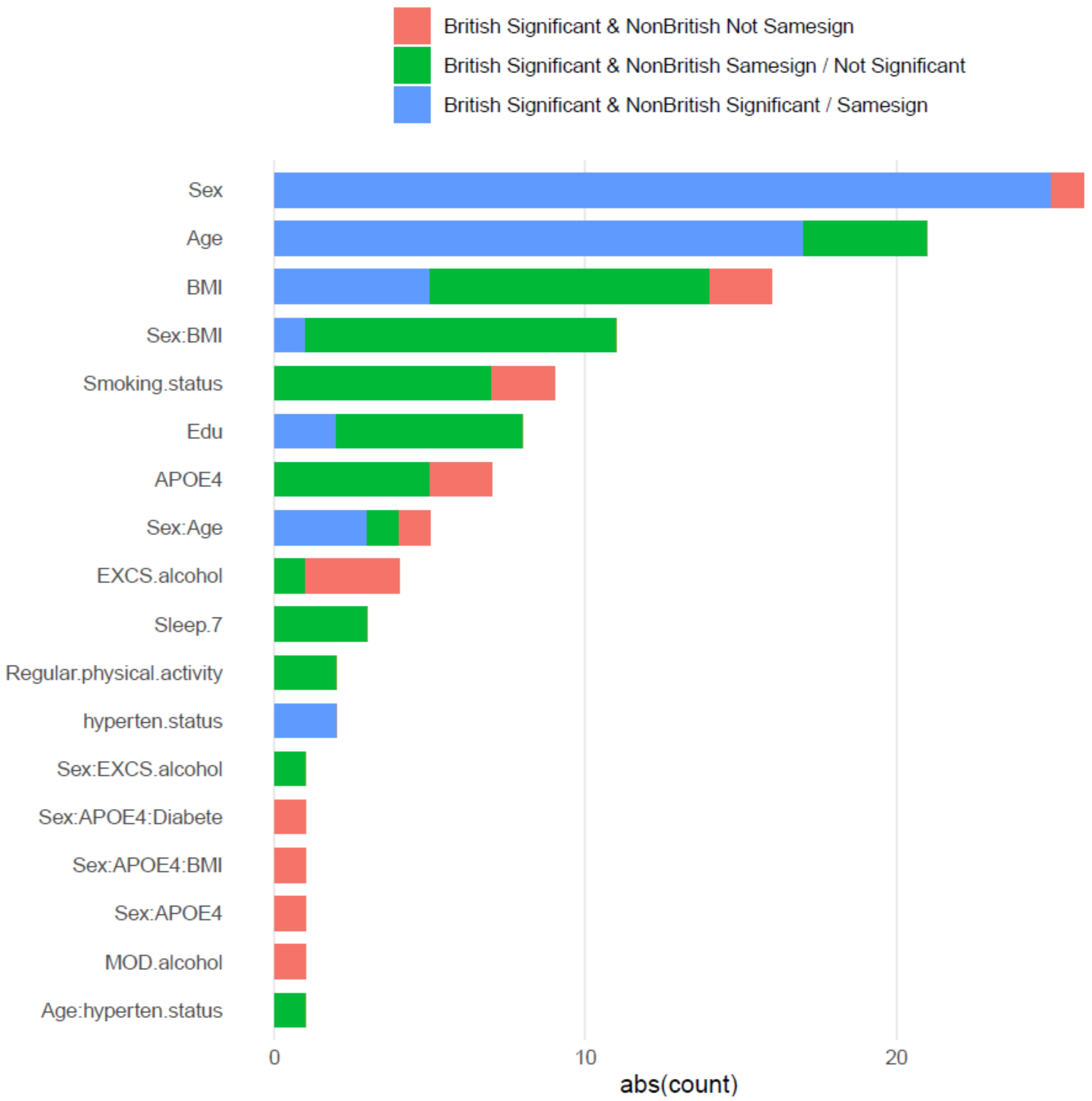

Supplementary Figure S39 Barplots of number of signals based on the Bonferroni significance level in the main discovery (n = 33,824) on network functional connectivities (NFCs) based on the Yeo-7 network atlas that have inconsistent directions (red) in non-British dataset (n = 2806), have consistent directions that are significant (blue) or non-significant (green).

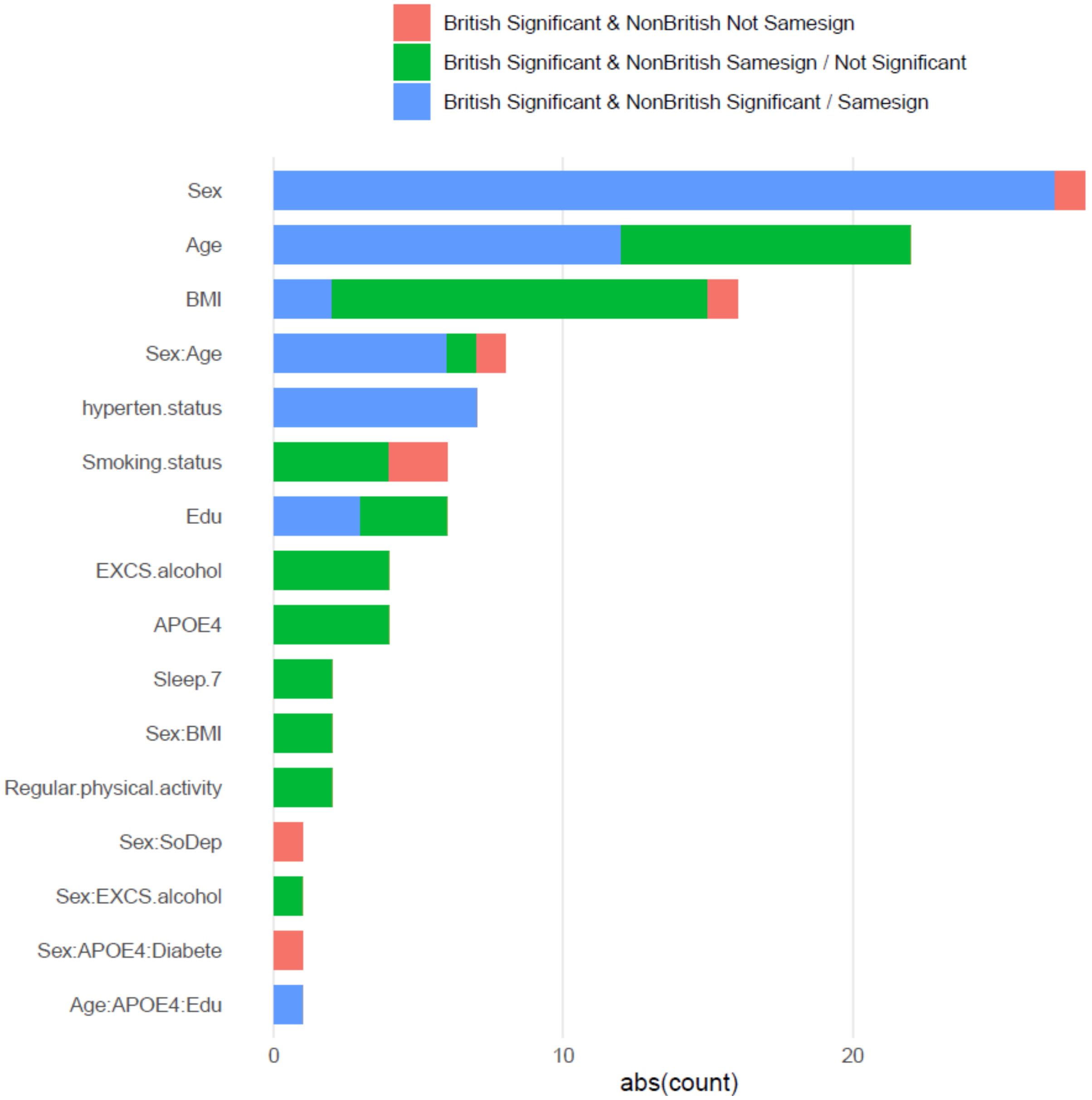

Supplementary Figure S40 Barplots of number of signals based on the Bonferroni significance level in the main discovery (n = 33,824) on network edge strength (NES) measures based on the Yeo-7 network atlas that have inconsistent directions (red) in non-British dataset (n = 2806), have consistent directions that are significant (blue) or non-significant (green).

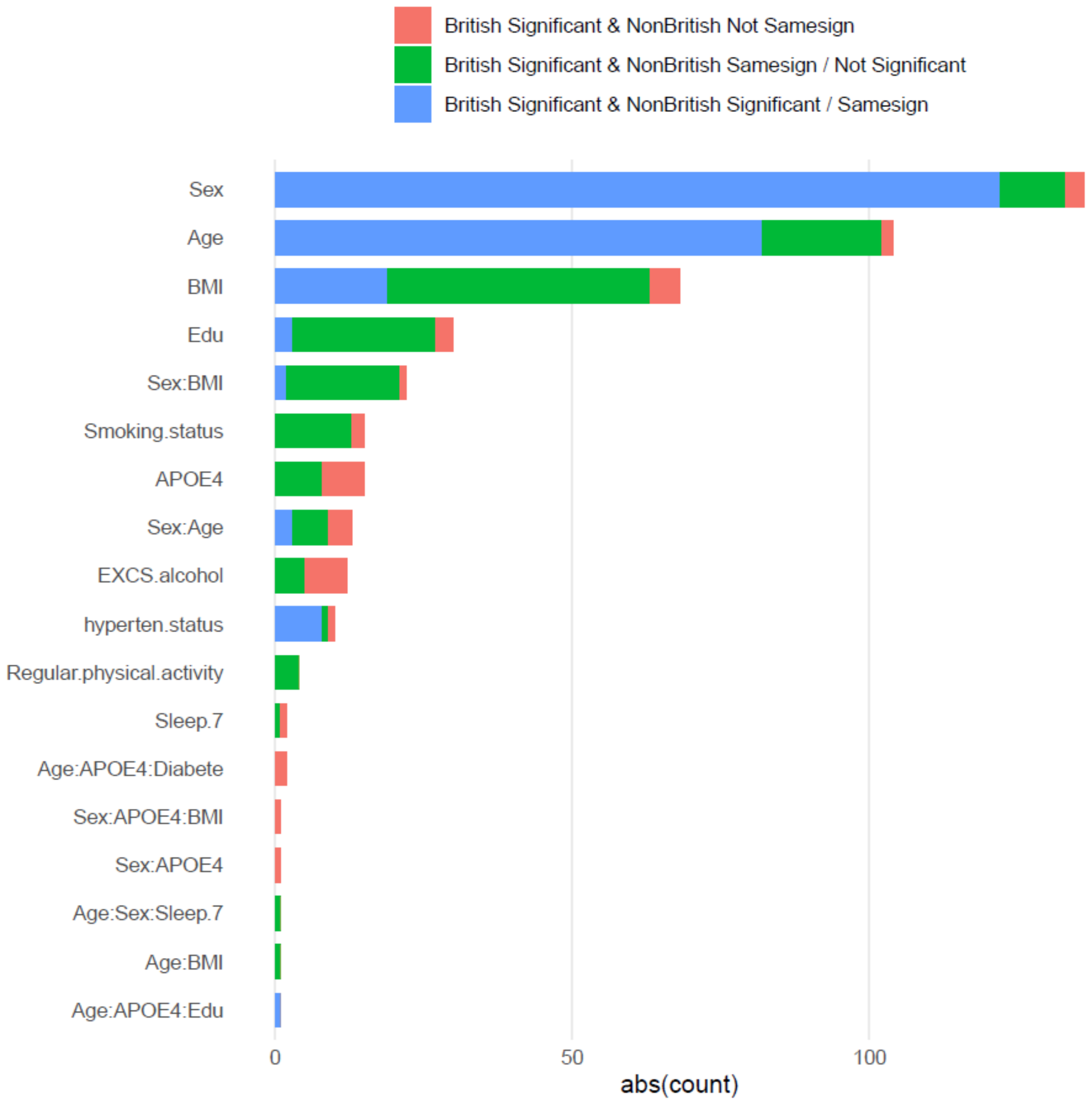

Supplementary Figure S41 Barplots of number of signals based on the Bonferroni significance level in the main discovery (n = 33,824) on network functional connectivities (NFCs) based on the Yeo-17 network atlas that have inconsistent directions (red) in non-British dataset (n = 2806), have consistent directions that are significant (blue) or non-significant (green).

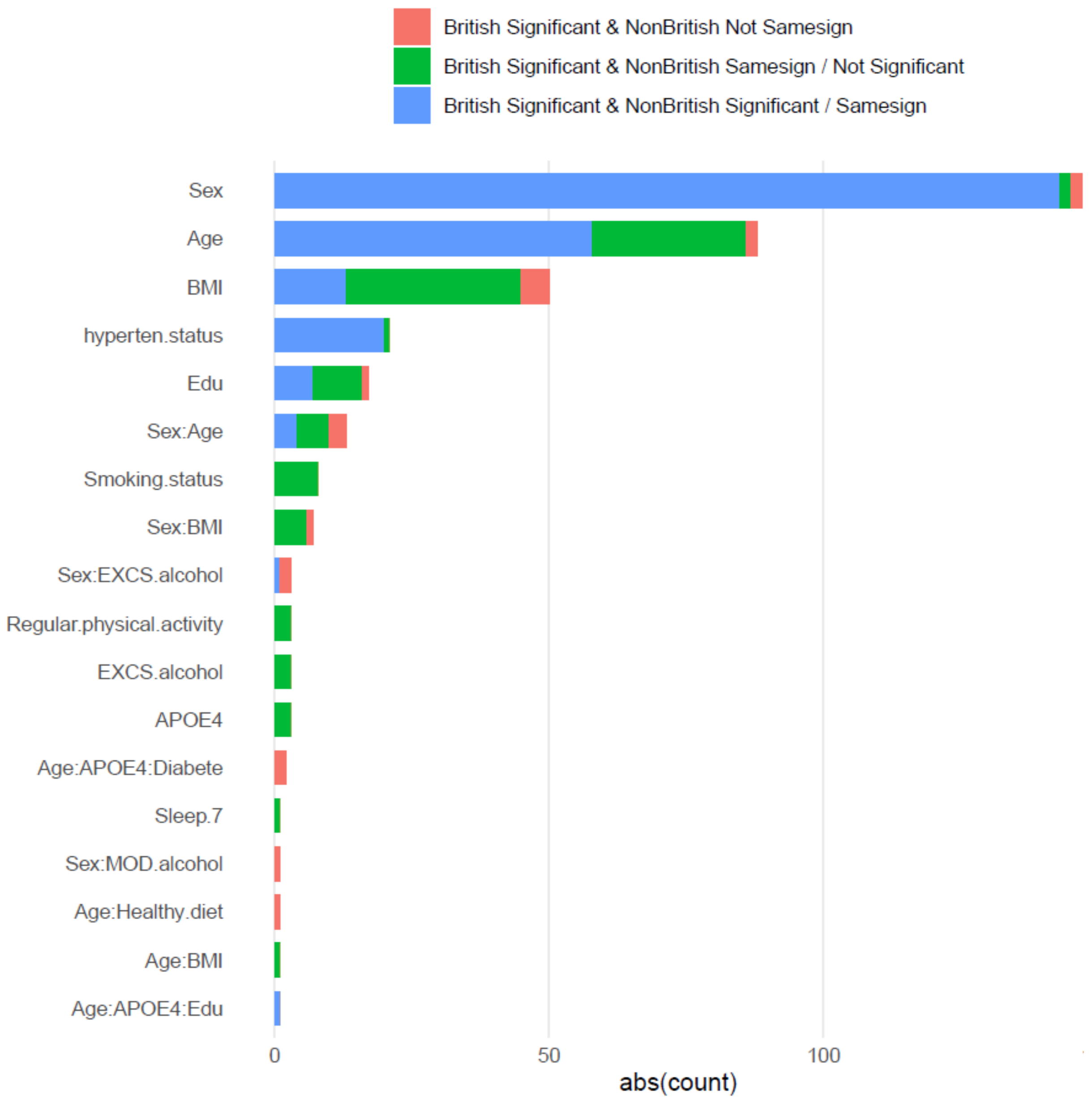

Supplementary Figure S42 Barplots of number of signals based on the Bonferroni significance level in the main discovery (n = 33,824) on network edge strength (NES) measures based on the Yeo-17 network atlas that have inconsistent directions (red) in non-British dataset (n = 2806), have consistent directions that are significant (blue) or non-significant (green).

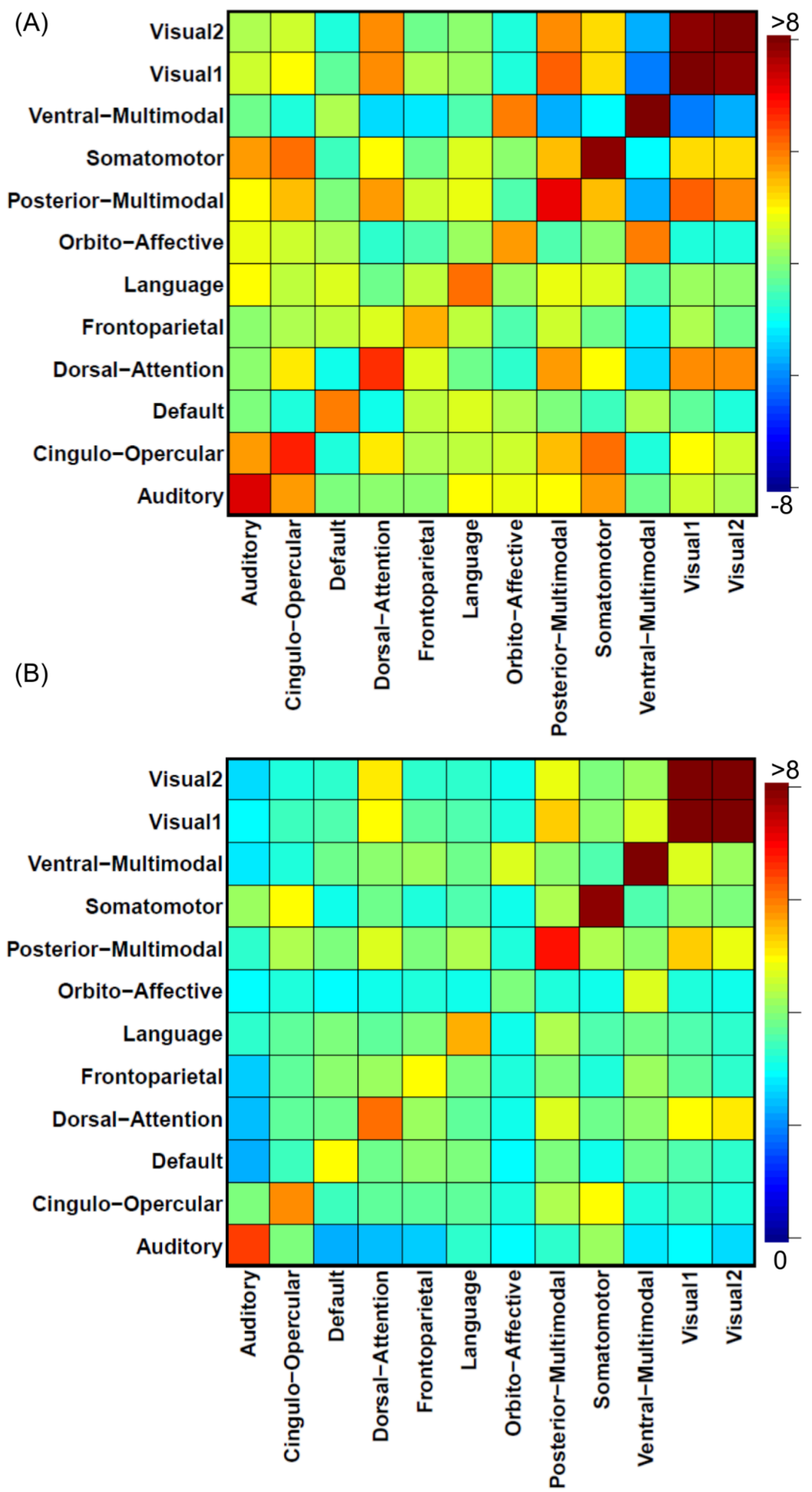

Supplementary Figure S43 Heatmap to illustrate the mean network functional connectivity (NFC) (A) and network edge strength (NES) (B) matrices based on the Ji-12 network atlas across the population (36,630 subjects).

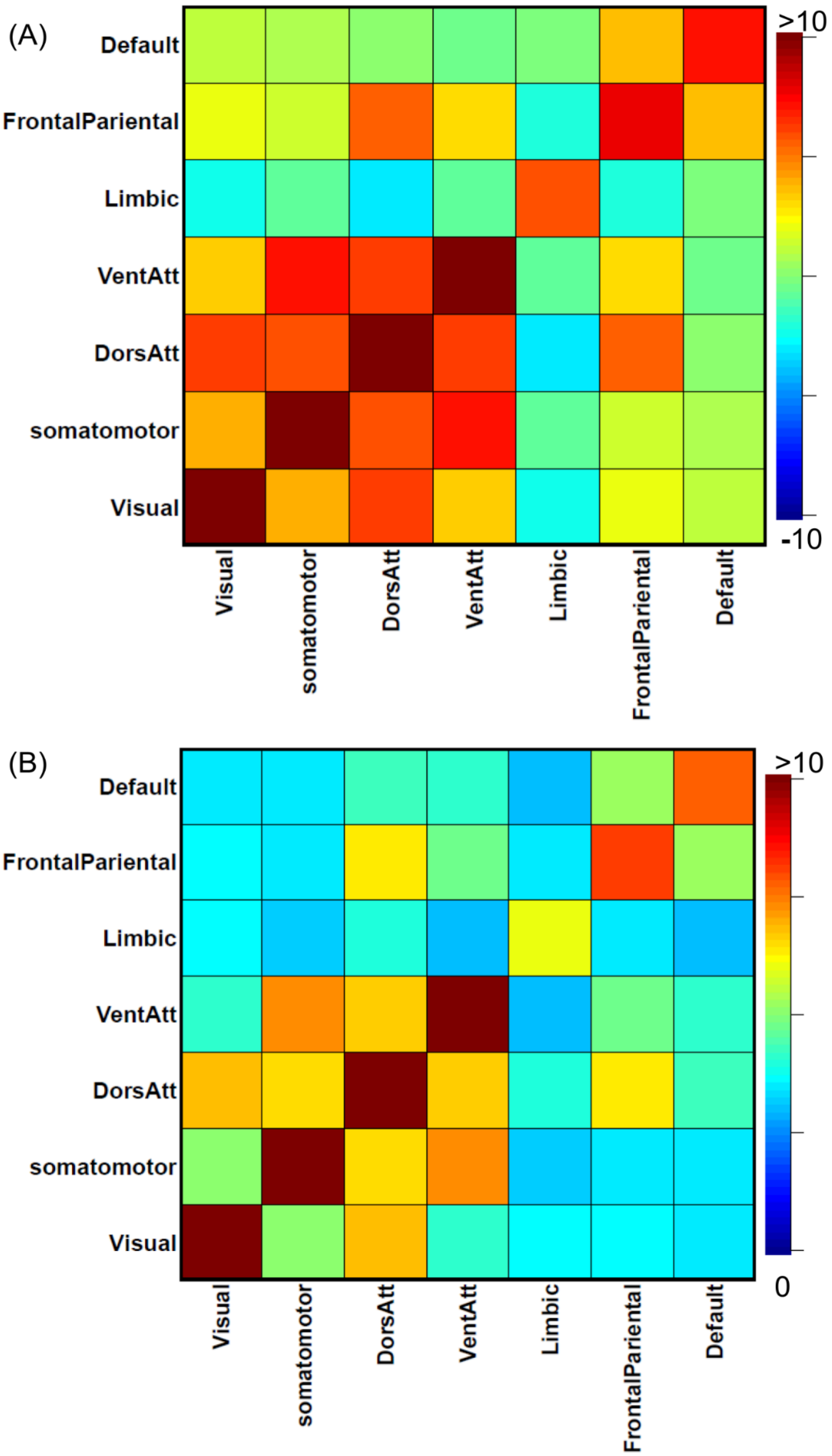

Supplementary Figure S44 Heatmap to illustrate the mean network functional connectivity (NFC) (A) and network edge strength (NES) (B) matrices based on the Yeo-7 network atlas across the population (36,630 subjects).

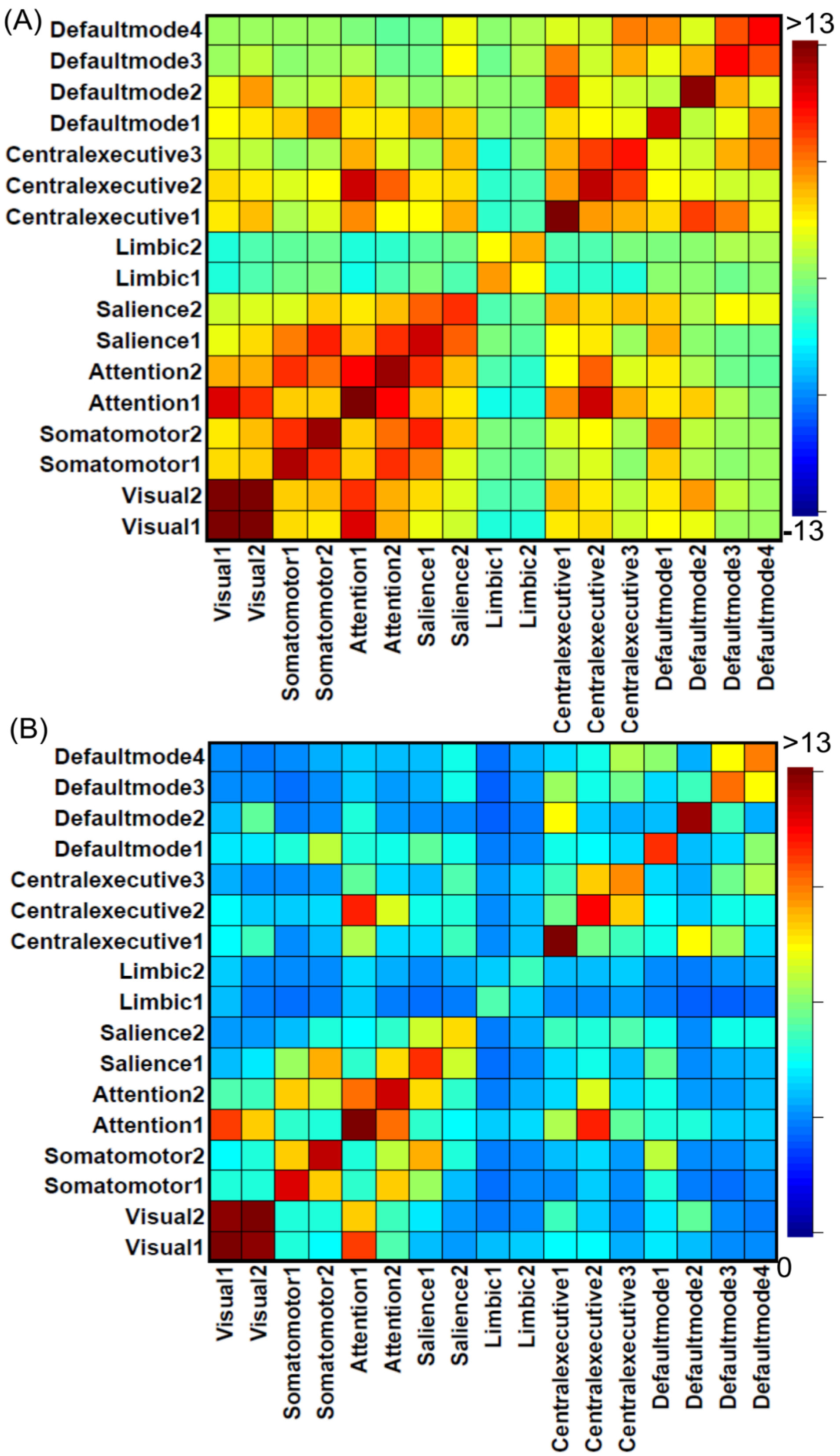

Supplementary Figure S45 Heatmap to illustrate the mean network functional connectivity (NFC) (A) and network edge strength (NES) (B) matrices based on the Yeo-17 network atlas across the population (36,630 subjects).

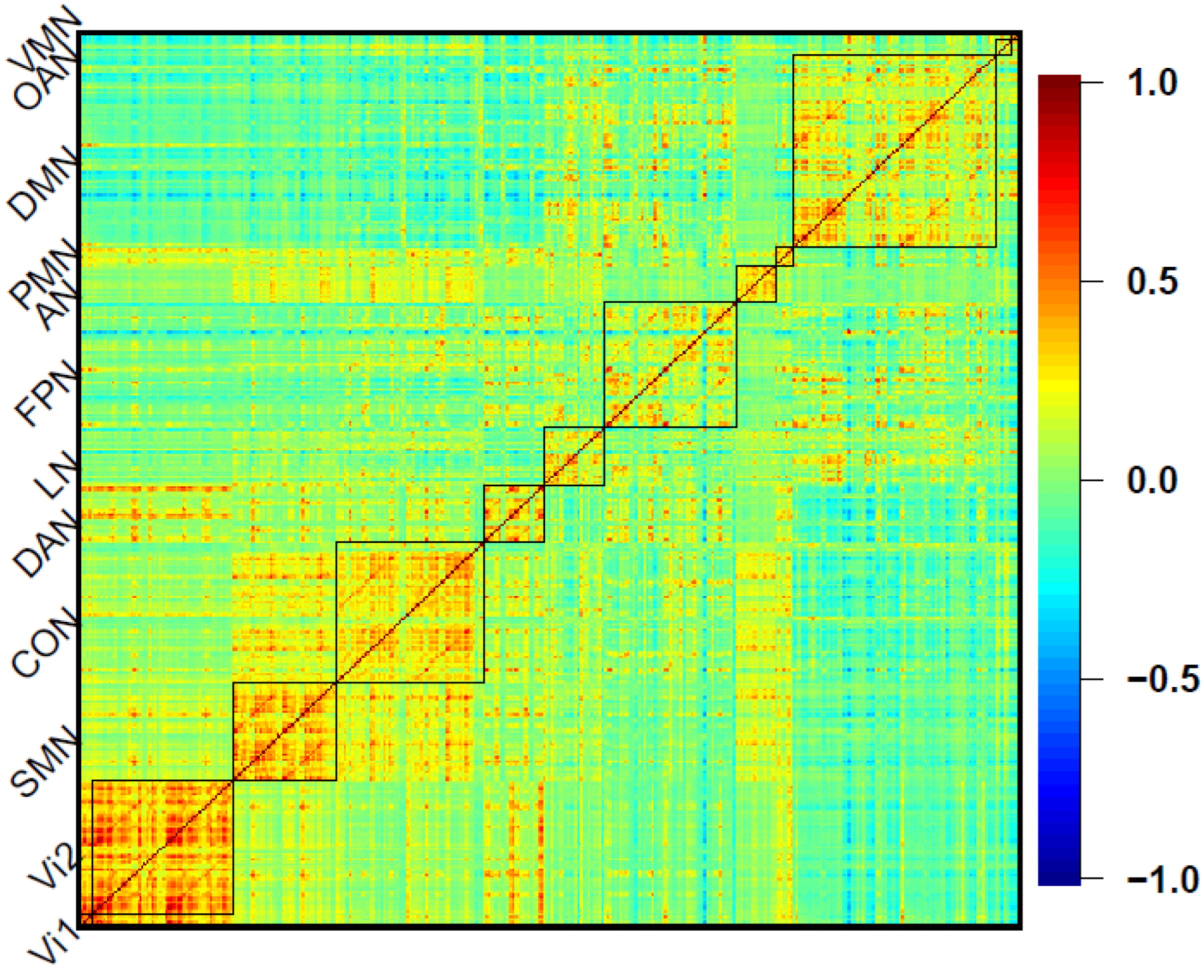

Supplementary Figure S46 The functional connectivity map of the Glasser-360 atlas and Ji-12 network atlas across the population (36,630 subjects). The regions are organized to show region pairs within the same network together.

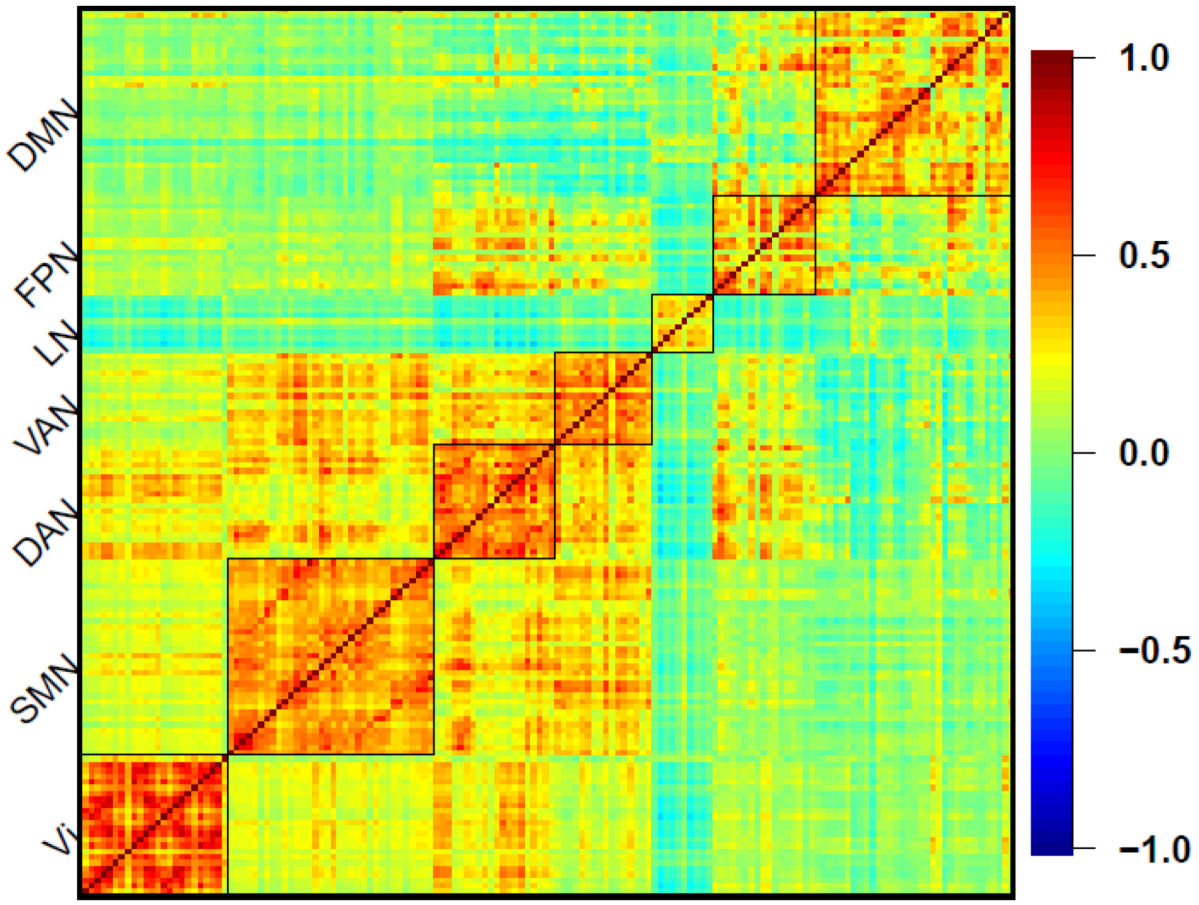

Supplementary Figure S47 The functional connectivity map of the Schaefer-200 atlas and Yeo-7 network atlas across the population (36,630 subjects). The regions are organized to show region pairs within the same network together.

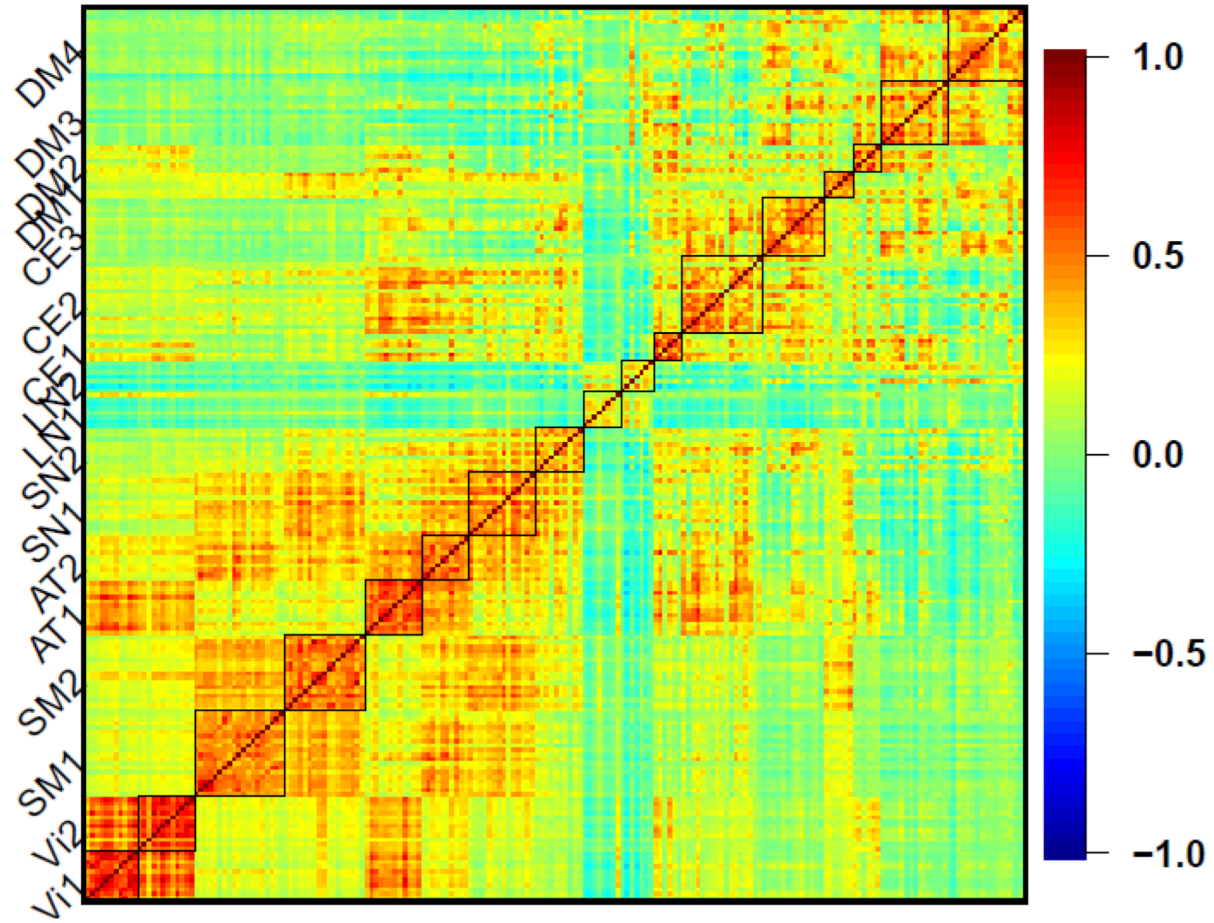

Supplementary Figure S48 The functional connectivity map of the Schaefer-200 atlas and Yeo-17 network atlas across the population (36,630 subjects). The regions are organized to show region pairs within the same network together.

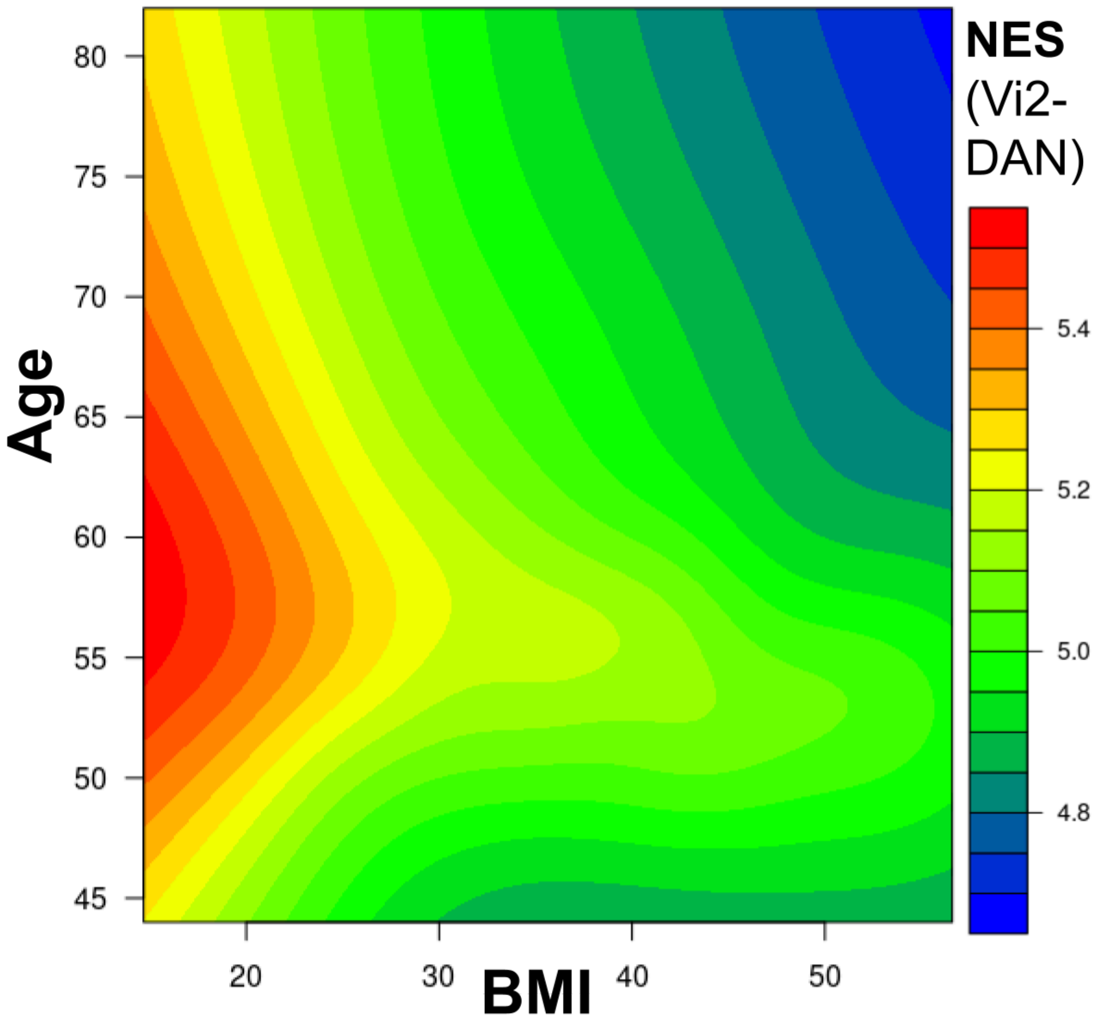

Supplementary Figure S49 The contour plot of the NES measures between visual2 and dorsal attention networks versus Age and BMI across the white British population (n = 33,824), indicating a negative age-BMI interaction effect. The estimated bivariate function of age and BMI is fitted through
